# Polymorph‐Specific Electronic Transduction in WO_3_ during Molecular Sensing

**DOI:** 10.1002/adma.202516840

**Published:** 2026-03-28

**Authors:** Matteo D'Andria, Meng Yin, Stefan Neuhauser, Vlasis G. Mavrantzas, Ying Chen, Ken Suzuki, Andreas T. Güntner

**Affiliations:** ^1^ Human‐Centered Sensing Laboratory Department of Mechanical and Process Engineering ETH Zurich Zurich Switzerland; ^2^ Department of Finemechanics Graduate School of Engineering Tohoku University Sendai Miyagi Japan; ^3^ Macromolecular Engineering Laboratory Department of Mechanical and Process Engineering ETH Zurich Zurich Switzerland; ^4^ Department of Chemical Engineering University of Patras & Institute of Chemical Engineering Sciences (ICE–HT/FORTH) Patras Greece; ^5^ Global Learning Center Tohoku University Sendai Miyagi Japan; ^6^ Green X‐Tech Center Green Goals Initiative Tohoku University Sendai Miyagi Japan

**Keywords:** electronic structure, gas sensors, nanotechnology, semiconductors, surfaces

## Abstract

Polymorphs are distinct structural forms of the same compound and offer unique opportunities to tailor material properties without altering chemical composition. In particular, the polymorphs of WO_3_ have been widely explored for their molecular sensing performance; yet, the mechanistic aspects behind their different chemoresistive properties have remained elusive or poorly understood. Here, we highlight the energetic allocation of transferred charge as a critical aspect for chemoresistive response generation, providing a new perspective beyond more conventional net‐transfer metrics, which are usually deployed to investigate gas–solid interactions. To this, we combined *operando* work function, chemisorption analysis, and in situ spectroscopy with density functional theory calculations on the example of acetone. Both *γ*‐ and *ε*‐WO_3_ exhibit comparable surface‐level activation of acetone, mediated by electron‐deficient, coordinatively unsaturated tungsten sites. However, only *ε*‐WO_3_ stabilizes analyte‐induced electronic states derived from W(5d) orbitals lying just below the conduction band—an energetically favorable region for conductivity modulation under operating conditions. While being associated with marginal work function shifts, these states reflect deeper subsurface electronic rearrangements that may underlie the *ε*‐WO_3_’s superior transduction efficiency despite similar receptor chemistry. Our results offer a new framework for rational transducer development rooted in intrinsic electronic structure.

## Graphical abstract

Metal‐oxide polymorphs with similar surface chemistry can nevertheless exhibit distinct sensing properties. In *γ*‐ and *ε*‐WO_3_, analyte adsorption appears comparable; yet, only *ε*‐WO_3_ induces a pronounced lattice electronic perturbation that accommodates charge in sub‐conduction band minimum states. This polymorph‐specific electronic structure adaptation underpins the stronger chemoresistive response of *ε*‐WO_3_ and illustrates how subtle lattice motifs govern transduction.

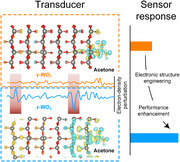

## 1 Introduction

Thermally activated transformations on nanostructured surfaces, exploited in chemoresistive gas sensing [1], heterogeneous catalysis [2] or energy conversion [3], are fundamentally governed by charge transfer and redistribution [4]. These electron‐driven processes underpin all oxidation chemistry [5]—from model reactions such as the total oxidation of CO [6] and larger volatile organic compounds (e.g., alcohols [7], ketones, and aromatics [8]), to more challenging selective oxidations, including the conversion of methanol to formaldehyde [9], and ammonia to nitrogen [10] or nitrous oxide [11]. Furthermore, electron transfer governs the binding strength of reactants [12], the relative stability of intermediates [13], and ultimately the product distribution under reaction conditions [14].

Mechanistic studies usually emphasize as performance descriptors at the gas–solid interface reactant conversion, product selectivity, and changes in oxidation state, but often ignore the role of electron transfer in modifying the electronic structure of semiconductor metal oxides (MO_x_). For supported metal catalysts, electronic descriptors such as *d*‐band center (*ε*_d_) relative to the Fermi level (*E*_F_) have been widely used to rationalize adsorbate energetics [15, 16] and catalytic trends [17]. However, this description is limited in d^0^‐type [18] MO_x_ such as WO_3_ [19], where *ε*_d_ is either ill‐defined or irrelevant, due to the absence of a pseudo‐continuum of occupied *d*‐states near *E*_F_. Furthermore, the formation of localized electronic states [20] induced by charge transfer within the adsorbate‐substrate complex is rarely resolved with respect to intrinsic band structure quantities [21], including the valence and conduction band edges or mid‐gap states.

In heterogeneous catalysis, resolving how transferred electrons are accommodated within the material is often secondary to understanding the dynamics of surface‐bound intermediates and selective bond activation [22]. In contrast, in chemoresistive sensors based on semiconductors, the key observable is the change in electrical resistance [23] of the solid itself, which directly reflects modifications in the electronic structure upon analyte‐surface interaction. In this context, charge transfer not only affects surface chemistry but may also induce the formation of localized states within the band structure, thereby altering conductivity.

Tungsten trioxide (WO_3_) is among the most studied MO_x_ for chemoresistive gas (e.g., acetone [24, 25, 26, 27], NO_2_ [28], isoprene [29], toluene [30]) detection, with numerous reports [31, 32, 33, 34] discussing its thermodynamically stable *γ*‐phase. Also studied is the metastable *ε*‐WO_3_ polymorph [35] that has consistently shown higher responses, for instance to acetone, under comparable conditions [36, 37]. This crystal phase has been implemented in advanced sensing platforms, including clinical tests for non‐invasive metabolic monitoring via skin‐ and breath‐acetone analysis [38, 39, 40] and has been deployed in diverse contexts, such as locating entrapped individuals [41] and supporting patient care in clinical settings [42], with potential for future use in monitoring astronauts’ health during space missions [43]. Already in its original synthesis report as a molecular sensor [44], enhanced performance of *ε*‐WO_3_ was tentatively linked to ferroelectric surface domains, suggesting a coupling between crystal structure and sensing function. Beyond its sensing applications, WO_3_ has attracted considerable interest as a model system for exploring fundamental electronic phenomena in transition‐metal oxides, particularly the formation of electron and hole polarons and their associated charge‐carrier transport behavior [45]. Yet and despite considerable progress, the mechanistic origin of *ε*‐WO_3_’s superior response remains unclear. Conventional surface‐chemical [46] insights—based on adsorbate binding, oxygen activation, or intermediate speciation—have not captured the performance gap, while the impact of polymorphism on electron transfer and localized electronic states remains unexplored.

Here, we investigate how structural polymorphism governs the sensing response of WO_3_ by an in‐depth comparison of its *γ*‐ and *ε*‐phases on the example of acetone. Through a combination of in situ spectroscopy, chemisorption analysis, electronic structure calculations, and simultaneous electrophysical measurements (work function, *φ*, and DC resistance, *R*), we relate analyte interaction to changes in the material's electronic structure. This enables us to move beyond conventional surface affinity considerations (adsorption energies) by assessing not only the extent of charge transfer (Bader charge), but also how and where charge is accommodated within the band structure to constitute a chemoresistive sensing response.

## 2 Results and Discussion

### 2.1 Material Structural Identity of WO_3_ Polymorphs

**FIGURE 1:**
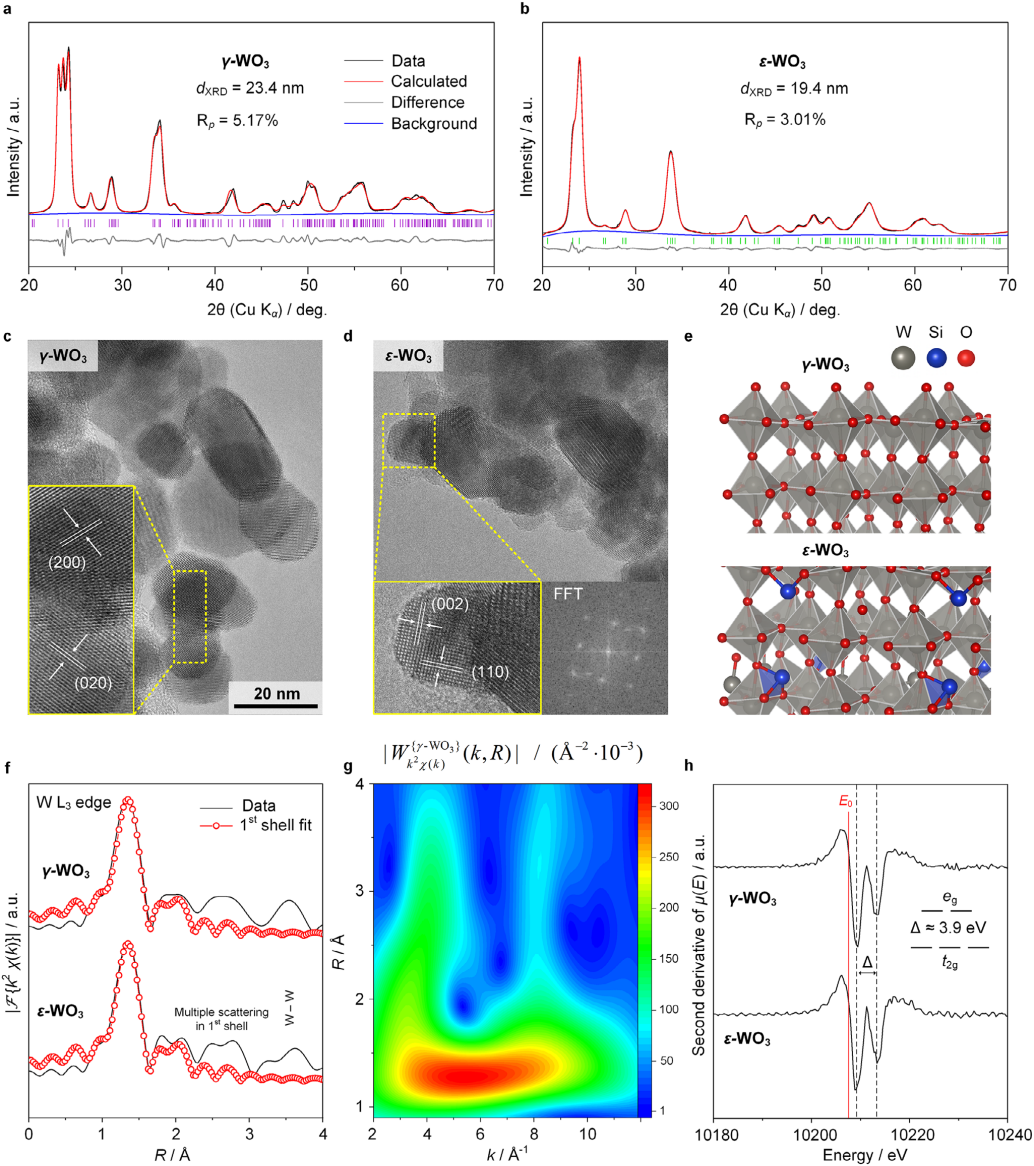
XRD patterns and Rietveld refinements for (a) *γ*‐WO_3_ as well as (b) *ε*‐WO_3_ with 10 mol% Si, with indicated coherent crystallite sizes of WO_3_ polymorphs, fit residuals, and background. High‐resolution TEM images of (c) *γ*‐ and (d) *ε*‐WO_3_ at same scale. The inset in (c) indicates the (200) and (020) planes of *γ*‐WO_3_ corresponding to interlayer spacings (*d*) of 3.7 and 3.8 Å, respectively. The insets in (d) highlight an *ε*‐WO_3_ nanocrystal oriented along [–110] and its respective FFT. (e) DFT‐relaxed structures, showing the coordination of W sites in *γ*‐WO_3_ (top) and *ε*‐WO_3_ with interstitial Si incorporation (bottom). Red, oxygen atom; gray, tungsten atom; blue, silicon atom. (f) W‐L_3_‐edge Fourier transforms of *k* ^2^‐weighted *χ*(*k*) together with first‐shell fits, along with the (g) corresponding wavelet transform (WT, magnitude) for *γ*‐WO_3_. Further WT analysis is reported in Figures S4 and S5. (h) Second derivative of XANES where the edge position (*E*_0_) and crystal field splitting (Δ) are also indicated.

We harnessed non‐equilibrium combustion‐aerosol technology in the form of flame spray pyrolysis (FSP) [47], where rapid formation and quenching of nanoscale oxides kinetically trap *ε*‐WO_3_ [44], as similarly shown also for pseudo‐binary metal oxides [48]. *ε*‐WO_3_ is the low‐temperature (<−50°C) monoclinic phase of WO_3_ [49], and is therefore metastable under ambient or sensing conditions. To ensure stability at operating temperatures, foreign element doping (i.e., Cr [44] or Si [36]) prevents restructuring into the thermodynamically stable *γ*‐polymorph upon annealing. Figure 1a,b shows the X‐ray diffraction (XRD) patterns of WO_3_ powders as synthesized without (a) and with (b) 10 mol% Si, along with structural refinement results. In agreement with the literature [36], phase‐pure *γ*‐ and *ε*‐polymorphs are obtained, as confirmed by statistical fit quality (*R*_p_) and lattice parameters matching reference values (Table S1) with, however, possible *ε*‐remnants in the pure WO_3_ sample (Figure S1).

As a result, defect incorporation stabilizes phase‐pure *ε*‐WO_3_ nanoparticles with slightly suppressed growth and larger surface areas [50] (Figure S2), in agreement with high‐resolution transmission electron microscopy (HR‐TEM) images in Figure 1c,d, both showing faceted nanoparticles and extended fringe patterns. The inset of Figure 1c indicates the *γ*‐WO_3_’s lattice planes (200) and (020) with interlayer spacings (*d*) of 3.7 and 3.8 Å, respectively. Similarly, the inset of Figure 1d highlights a nanocrystal with two sets of perpendicular fringes, as confirmed by its approximately cubic fast Fourier transform (FFT). This is consistent with the (002) and (110) spacings of an *ε*‐WO_3_ crystal oriented along [–110], where the fringe‐specific assignment is tentative due to very similar *d*‐values of ≈3.8 Å that are indistinguishable within the measuring accuracy of the method.

Given the large discrepancy in ionic radii [51] and charge imbalance between W^6+^ and Si^4+^, as well as the differing coordination preference – octahedral [52] (O_h_) WO_6_ vs. tetrahedral [53] (T_d_) SiO_4_ – Si‐related units are likely incorporated interstitially, forming non‐substitutional local environments between WO_6_ octahedra. This is supported by structural relaxation via density functional theory (DFT) calculations as shown in Figure 1e, where both *γ*‐ and *ε*‐WO_3_ frameworks exhibit tilted, corner‐sharing WO_6_. Silicon atoms in the *ε*‐WO_3_ structure are found outside the first tungsten shell, forming an incomplete T_d_, distorted pyramid‐like coordination with a missing bond, similarly obtained at different Si% (Figure S3). Such low‐coordinated SiO_x_ could induce local lattice distortions that kinetically stabilize *ε*‐WO_3_ under microstrained (0.253% deformation, Table S1) conditions.

To probe the tungsten coordination environments, we conducted L_3_‐edge X‐ray absorption fine structure (XAFS) spectroscopy, extracting the Fourier‐transformed (FT) *k* ^2^‐weighted *χ*(*k*) signal, as shown in Figure 1f. Quantitative analysis of the first shell is summarized in Table S2. The two phases exhibit nearly identical W─O bond lengths and mean‐square displacements, with coordination numbers of 5.48 and 5.24 for *γ*‐ and *ε*‐WO_3_, respectively, both lower than expected for ideal O_h_ symmetry, likely due to oxygen vacancies (V_O_) introduced during flame synthesis [54].

The wavelet transform (WT) of *γ*‐WO_3_ (Figure 1g) features a broad *k*‐distribution for 1.0 Å < R < 1.6 Å consistent with local WO_6_ structural heterogeneity [55], captured by modeling distinct crystallographic W‐O environments [56] (Table S2). The WT of *ε*‐WO_3_ (Figure S4) similarly reveals inhomogeneous WO_6_ coordination, accompanied by multiple scattering contributions for *R* > 1.7 Å. Subtracting the *γ*‐WO_3_’s contour map from that of *ε*‐WO_3_ (Figure S5a) highlights additional spectral intensity in the range 1.5 Å < *R* < 2.5 Å and at larger wavenumbers (>9 Å^−1^). These features are consistent with the presence of strained, T_d_‐like SiO_x_ located beyond the first W coordination shell, as indicated by the WT signatures of ad hoc constructed scattering paths [57] including single and multi‐scattering from Si atoms (Figure S5b–d). Second‐derivative W L_3_‐edge XANES analysis in Figure 1h shows identical ligand field‐induced 5d splitting [56] and W^6+^ oxidation states for *γ*‐ and *ε*‐WO_3_ (Figure S6), confirming that Si incorporation leaves the local WO_6_ structure unperturbed.

### 2.2 Electronic Structure of WO_3_ Polymorphs

**FIGURE 2:**
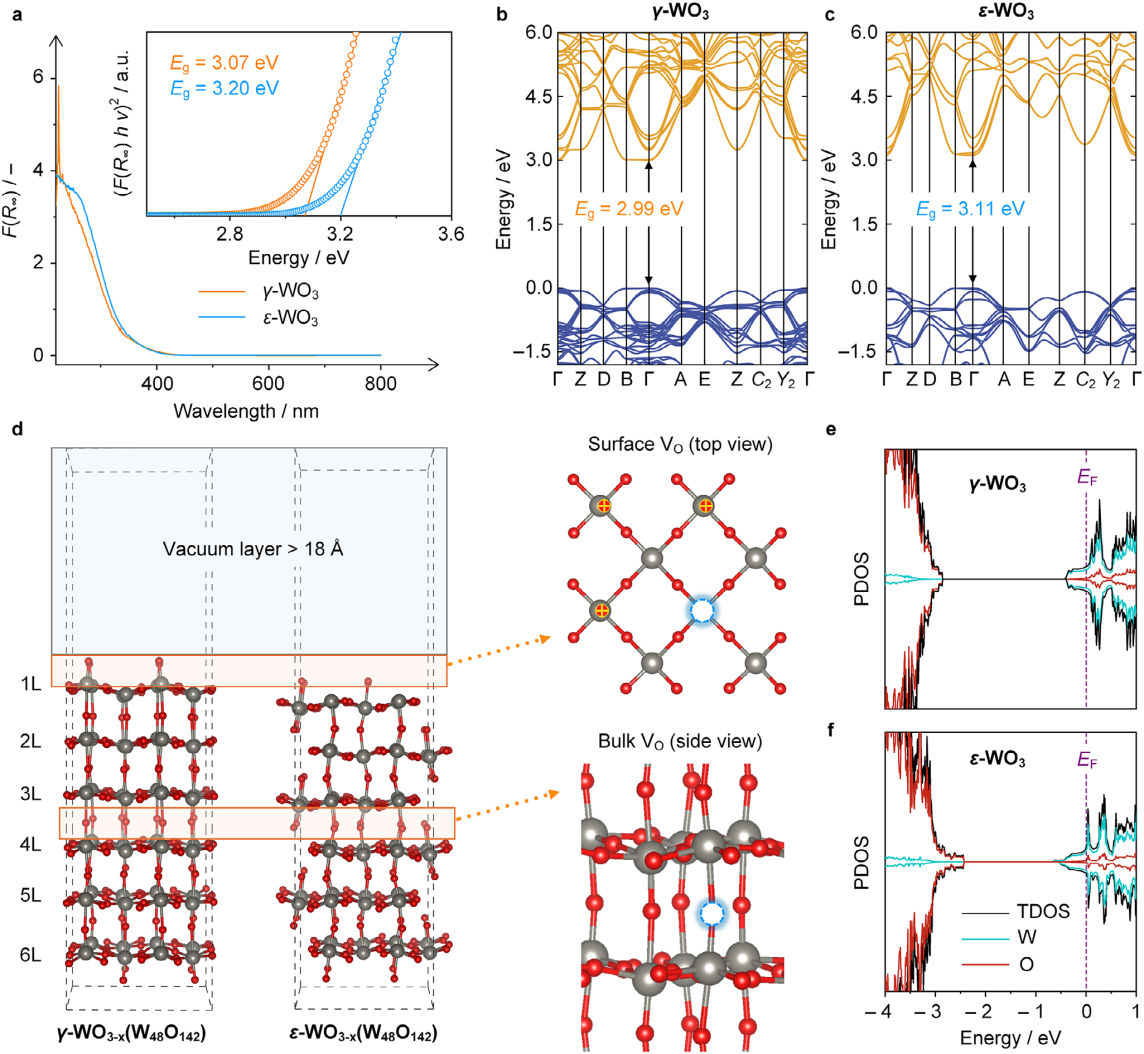
(a) UV–Vis spectra and *E*_g_ determination for *γ*‐WO_3_ and *ε*‐WO_3_. Calculated band structures, with valence band (blue) and conduction band (orange) states, for the unit cells of (b) *γ*‐WO_3_ and (c) *ε*‐WO_3_. (d) Slab models of *γ*‐WO_3_ (001) and *ε*‐WO_3_ (001), with a zoomed‐in view illustrating the introduction of bulk and surface oxygen vacancies (V_O_) in the *γ* polymorph. Red, oxygen atom; gray, tungsten atom. The highlighted (with yellow crosses) oxygen atoms in the top view are surface atoms. (e,f) Projected density‐of‐states (PDOS) graphs for slab models of the two WO_3−x_ phases with bulk and surface V_O_.

A reliable electronic structure model is essential for understanding charge transfer and localized states in WO_3_ polymorphs; we therefore first establish a validated DFT‐based description, grounded in experimental observations. For *ε*‐WO_3_, Si atoms were not included in the model, consistent with its phase purity (Figure 1b) and the unaffected position of t_2g_ and e_g_ states (Figure 1h). UV–Vis diffuse reflectance spectra (DRS) of *γ*‐ and *ε*‐WO_3_ (Figure 2a) display a steep absorption increase near 400 nm, attributed to O(2p) → W(5d) ligand‐to‐metal charge transfer [58], reflecting valence band (VB) to conduction band (CB) excitations. Both polymorphs exhibit sharp optical profiles, consistent with phase‐pure materials, although the presence of weakly optically active mid‐gap defects, such as V_O_‐induced shallow donors [59], cannot be excluded. Tauc analysis (see inset in Figure 2a) yields optical bandgaps (*E*_g_) of 3.07 and 3.20 eV for *γ*‐ and *ε*‐WO_3_, respectively, and the linearity of (*F*(*R*_∞_) · *h*ν)^2^ vs. photon energy confirms the direct [60] VB → CB character, in agreement with literature [61].

Next, the electronic band structures of *γ*‐ and *ε*‐WO_3_ were calculated using DFT. To address the well‐known *E*_g_ underestimation of standard GGA functionals [62], the DFT‐1/2 approach proposed by Ferreira and co‐workers [63, 64] was employed, which has also been used in previous works to describe *γ*‐WO_3_ [26, 30]. This method introduces half‐occupation corrections to effectively capture self‐energy effects without the need for empirical parameters, unlike DFT+U, and has proven reliable for weakly correlated oxides, such as WO_3_ [26, 65]. The DFT‐1/2 scheme considered here enables an improved bandgap description at manageable computational cost for the large supercells employed in our calculations. Hybrid functionals such as HSE06 [66] may further enhance the overall band‐structure accuracy and provide a more rigorous description of polaron formation [67, 68]. However, their application to large slab models (here around 200‐atom large), which also include adsorbates, surface, and subsurface oxygen defects, may be computationally less practical.

The calculated band structures in Figure 2 for (b) *γ*‐ and (c) *ε*‐WO_3_ reveal direct bandgaps located at the Γ‐point for both polymorphs. DFT‐1/2 yields *E*_g_ of 2.99 and 3.11 eV for *γ*‐ and *ε*‐WO_3_, respectively, closely matching the optical gaps extracted from the Tauc analysis (Figure 2a, inset). These results also align well with previously reported experimental [49, 69] values for *γ*‐WO_3_, and show markedly improved accuracy compared to DFT calculations [70, 71] using LDA or GGA‐PBE functionals without U‐type corrections (see Table S3). Note that the modeled structures are also correct crystallographic representations of the two phases, as calculated lattice constants of *γ*‐WO_3_ and *ε*‐WO_3_ (see Table S4) are in excellent agreement with our results (Table S1), as well as other experimental data [49, 69, 72] and previous theoretical studies [66].

Surface slab models of *γ*‐ and *ε*‐WO_3_ were constructed exposing the (001) facets to provide realistic platforms for investigating analyte‐surface interactions [73, 74]. Each slab comprised six atomic layers separated by >18 Å vacuum to eliminate periodic artifacts. To reflect the inherent nonstoichiometry of operating surfaces, as well as to ensure the presence of under‐coordinated sites capable of engaging in adsorption and electron transfer [75], surface and bulk V_O_ were introduced (W_48_O_142_ supercells, hereafter denoted as WO_3−x_), as shown in Figure 2d. This approach is consistent with our W‐L_3_‐XAFS analysis (Figure 1f) and the literature, recognizing that fully stoichiometric surfaces offer limited reactivity toward adsorbates under catalytic or sensing conditions [76]. Introduction of V_O_ results in the appearance of occupied states slightly below the CB minimum (Figure 2e,f) primarily derived from W 5d orbitals. Depending on V_O_ location (bulk or surface), these states exhibit different degrees of spatial localization across the slab, as confirmed by partial charge density mapping (Figure S7). The lower formation energies of surface‐ compared to subsurface‐V_O_ (Table S5) suggest their energetically shallow nature. Such defect‐induced states, acting as electron donors, are expected [46, 77] to play a critical role in modulating charge transfer processes at the surface of WO_3_.

### 2.3 Acetone Adsorption and Surface‐Chemical Interactions

**FIGURE 3:**
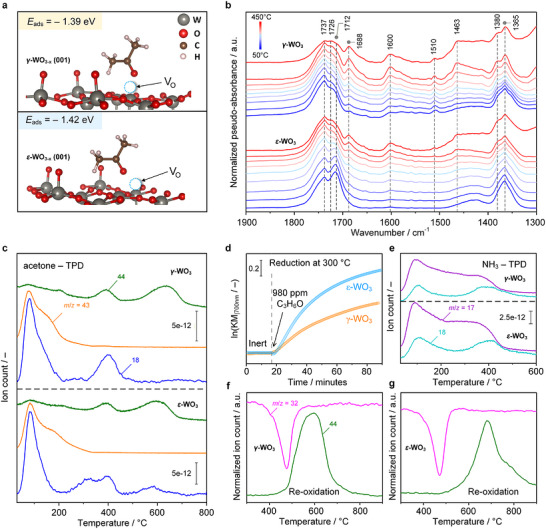
(a) Equilibrium structures of *γ*‐WO_3−x_ (001) and *ε*‐WO_3−x_ (001) with an adsorbed acetone molecule. Shown are the adsorption energies obtained by DFT. (b) In situ DRIFT spectra of adsorbed acetone (2 vol% C_3_H_6_O) between 50 and 450°C in steps of 50°C. Note that these pseudo‐absorbance spectra are normalized for spectral assignments. (c) Profiles of *m*/*z* = 44 (green), 43 (blue), and 18 (orange) during acetone–TPD for *γ*‐WO_3_ and *ε*‐WO_3_. (d) Transient absorbance at 700 nm (*d*‐*d* transition of reduced W^(6−^ *^δ^* ^)+^ species) measured upon exposure to acetone at 300°C. (e) Evolution of *m*/*z* = 17 (purple) and 18 (cyan) curves during NH_3_–TPD. Consumption and formation of O_2_ (*m*/*z* = 32) and CO_2_ (*m*/*z* = 44), respectively, during O_2_–TPO following acetone–TPR (Figure S15) over (f) *γ*‐WO_3_ and (g) *ε*‐WO_3_.

To elucidate analyte‐surface interactions, we combined first‐principles modeling with spectroscopic and thermochemical analysis. Adsorption geometries on *γ*‐ and *ε*‐WO_3−x_ surfaces were determined by DFT relaxation of acetone molecules placed near V_O_ (Figure 3a). In both polymorphs, acetone preferentially coordinates via its carbonyl oxygen to under‐coordinated tungsten centers adjacent to V_O_ sites. Interestingly, the calculated adsorption energies (*E*_ads_) are comparable for both polymorphs (i.e.,−1.39 and −1.42 eV for *γ*‐ and *ε*‐WO_3−x_, respectively). This is consistent with comparable net charge transfer from acetone to the substrate (Δ*Q*), and adsorption‐induced polarization leading to localized electron density on the carbonyl oxygen, as reflected by changes of local Bader charge (Δ*q_i_*, see Table S6). Note that slab‐size analysis in Figure S8 confirms that acetone binding strength is insensitive to surface area, validating the robustness of adsorption energetics.

In situ diffuse reflectance infrared Fourier transform spectroscopy (DRIFTS) during acetone exposure tracks the temperature‐dependent surface chemistry of *γ*‐ and *ε*‐WO_3_ (Figure 3b). To benchmark adsorption modes and isolate lattice O^2−^–enabled chemistry, we performed these measurements under high analyte concentrations (2 vol%). Gas‐phase acetone typically exhibits a single, sharp *ν*(C═O) near 1740 cm^−1^. Upon adsorption onto oxide surfaces, this carbonyl vibration undergoes electronic perturbation, resulting in multiple distinct [46] absorptions at 1712, 1726, and 1737 cm^−1^, reflecting interaction with surface tungsten centers. A weaker component emerging at 1688 cm^−1^ suggests a fraction of more strongly activated acetone species, possibly associated with partial C─O bond weakening. This interpretation is supported by increased fluctuations in the C─O bond length during a 10 ps ab initio molecular dynamics (AIMD) trajectory of acetone adsorbed on *γ*‐WO_3_ (001) at 500 K, as well as representative DRIFTS at lower acetone concentrations and in the (co‐)presence of O_2_ (Supporting Note S1, Figures S9–S11, and Supporting Movie S1).

At temperatures above 300°C, the carbonyl‐associated bands diminish while new features appear at 1600 and 1510 cm^−1^. The 1600 cm^−1^ band is consistent with the *ν*(C═C) of mesityl oxide [78], an intermediate of acetone condensation, while the 1510 cm^−1^ feature is attributed to enolate‐like species [79]. Additional absorptions at 1463 and 1365 cm^−1^ are tentatively assigned to carboxylate‐like [80] *ν*_asym_(COO^−^) and *ν*_sym_(COO^−^), respectively, which—together with a broad *δ*(CH_3_) shoulder near [81] 1380 cm^−1—^are consistent with the incipient formation of formate and/or acetate species [77]. This evolution reflects a stepwise acetone activation path, progressing from molecular adsorption to partial C─C coupling and oxygenate formation. Most importantly, across the temperature range including their typical operating conditions during acetone sensing (i.e., 300–400°C [24, 25, 26, 27, 31, 32, 33, 34]), *γ*‐ and *ε*‐WO_3_ exhibit closely similar surface reactivity, with only minor variations in band intensity and development.

Figure 3c shows thermal desorption profiles of acetone‐exposed *γ*‐ and *ε*‐WO_3_ under He, monitoring *m*/*z* = 43 (acetone), 44 (CO_2_), and 18 (H_2_O). Both polymorphs exhibit a sharp *m*/*z* = 43 peak at about 80°C, attributed to the release of physisorbed acetone. A secondary shoulder follows at ∼150°C and 180°C for *γ*‐ and *ε*‐polymorph, respectively, indicating slightly stronger retention or slower desorption kinetics in the *ε*‐phase. Above 200°C, oxygenated fragments begin to evolve: CO_2_ release above 300°C reflects lattice oxygen involvement in a Mars–van Krevelen (MvK) mechanism, consistent with WO_3_‐based catalysts for acetone sensing [28] as well as the expected role of V_O_ [82]. H_2_O evolution spans a broad range that could originate from dehydrogenation of surface‐bound acetates [83] or, in the case of *ε*‐WO_3_, desorption from Si‐bonded hydroxyls introduced during FSP synthesis. Overall, these trends align with the IR spectra shown in Figure 3b, where oxygenate and condensation products are observed within similar temperature ranges.

Extended surface‐chemical analysis (see also Figures S12–S16 and Supporting Note S2 for detailed discussion) further highlights the similarity of acetone interaction with *γ*‐ and *ε*‐WO_3_. We performed in situ UV–Vis DRS in the co‐presence of O_2_ and C_3_H_6_O, to evaluate the WO_3_ polymorphs in response to analyte dosing and removal to and from an O_2_‐containing background (Figure S12). As in the spectra acquired at room temperature (Figure 2a), absorption at <400 nm is dominated by the O(2p) → W(5d) LMCT whereas changes above ∼600 nm are associated with broader *d*‐*d* transitions of reduced W^(6‐δ)+^ species [84]. These results show that acetone adsorbs onto under‐coordinated tungsten sites, which feature strong Lewis‐acidic character, as identified by thermochemical desorption of basic probing molecules [85], i.e., NH_3_ (Figure 3e) and pyridine (Py, Figure S13). We selected the Kubelka‐Munk function, *F*(*R*_∞_), at 700 nm as a proxy for the population of such reduced tungsten species. The optical signal changes were transiently monitored during reactive gas interaction at 300°C. Interestingly, as represented in Figure 3d, analyte‐induced optical changes are more pronounced for ε‐WO_3_. Together with our combined chemisorption (Figure 3c), DRIFT spectroscopy (Figure 3b), and ab initio insights on adsorption energetics (Figure 3a), the observed UV–Vis–derived kinetics should not be attributed to faster intrinsic redox, but rather associated to a larger amount of acetone‐derived charge accommodated on the surface in frontier (i.e., in the proximity of *E*_F_) W(d) states.

Temperature‐programmed reductions (TPR) under different environments (H_2_, CO, acetone, Figures S14 and S15) evidence a measurable acetone‐induced redox activity around 300–400°C, that is, a notably higher reactivity than for H_2_ and CO (>500°C), possibly rationalizing their characteristically weak responses observed in WO_3_ chemoresistors. Finally, temperature‐programmed (re‐)oxidation (TPO) in O_2_ (Figure 3f,g) indicates the dynamicity and reversibility of the V_O_ population, which is a central aspect of MvK oxidations and is additionally corroborated by temperature‐programmed desorption (TPD) of CO_2_ (Figure S16). Summa summarum, our extensive surface‐chemical characterization indicates the rather identical surface properties of *γ*‐ and *ε*‐polymorphs; next, we assess their chemoresistive behavior.

### 2.4 Transducer Function and Electronic Structure Analysis

**FIGURE 4:**
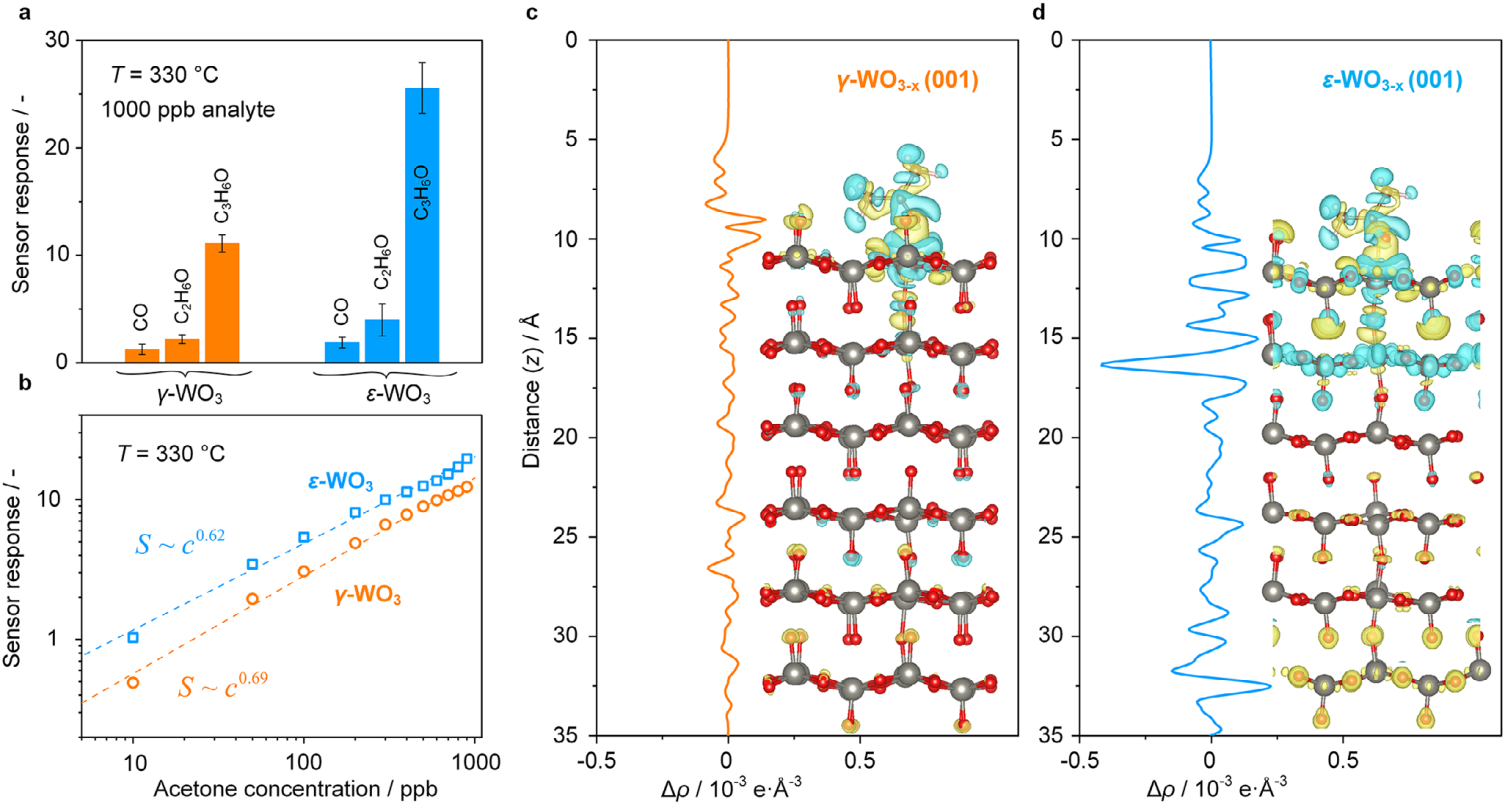
(a) Sensor response of *γ*‐WO_3_ (orange) and *ε*‐WO_3_ (blue) toward 1000 ppb CO, ethanol, and acetone, as well as (b) response vs. concentration characteristics of these for acetone ranging between 10 and 1000 ppb. The column heights and error bars are the average and standard deviation of three identically prepared sensors, operated at 330°C and under dry air conditions. CDD maps as well as plane‐averaged differential density (Δ*ρ*) plot as a function of *z*‐coordinate (i.e., normal to the slab) of acetone adsorbed on (001) surfaces of (c) *γ*‐WO_3−x_ and (d) *ε*‐WO_3−x_.

Despite similar acetone surface interaction (Figure 3), *ε*‐WO_3_ consistently exhibits higher responses to acetone as shown with three independent sensor devices, for instance, at 1000 ppb and 330°C in air (Figure 4a). This enhancement is observed between 10 and 1000 ppb (Figure 4b; Figure S17), where both polymorphs feature linear log‐log response characteristics. The slightly lower power law exponent of *ε*‐WO_3_ suggests differences in the underlying charge transport or modulation mechanism [86], which cannot be rationalized by surface reactivity that governs the receptor (i.e., acetone adsorption, see section 2.3) [27]. but rather indicate that structural polymorphism directly affects the transducer function. Note that approx. 330°C is the optimum operating temperature for both polymorphs (Figure S18), likely arising from a favorable balance of analyte‐driven reduction (V_O_ formation) and re‐oxidation (V_O_ formation), as suggested by our in situ analysis of W─O lattice overtone and combination features (Figure S19 and S20 and Supporting Note S3).

Figures 4c and d indicate the plane‐averaged differential charge density Δ*ρ*(*z*) derived from DFT along the slab depth (*z*) for *γ*‐ and *ε*‐WO_3−x_ (001), respectively, highlighting how electronic rearrangements extend into the material upon acetone adsorption. Both materials exhibit a net electron transfer from acetone to WO_3−x_ following molecular binding—consistent with increased conductivity in n‐type WO_3_. Yet, only *ε*‐WO_3−x_ shows more pronounced subsurface electronic perturbations, several atomic layers beneath the surface, extending into the bulk‐like region of the slab. This suggests that charge redistribution reaches beyond few topmost layers into interior domains where electronic transport occurs. This is further supported by Figures S21 and S22, which compare differential charge densities across both stoichiometric and reduced surfaces. While CO induces only limited perturbations—also in the presence of V_O_ that generally amplifies [87] Δ*ρ* amplitude (e.g., Figure S22a,b vs. Figure S22c,d)—acetone adsorption on *ε*‐WO_3−x_ in Figure 4d exhibits the most pronounced subsurface modulation. This reflects a synergistic interplay of polymorphism, V_O_ population, and analyte‐specific binding that results in the highest chemoresistive response. Note, however, that the Δ*ρ*(*z*) amplitude does not explain, for instance, *γ*‐WO_3_‘s larger response to acetone compared to CO (see Figure 4c; Figure S22c). In fact, in this case, the more conventional surface‐chemical approach is conclusive already, and provides both: (i) improved analyte‐induced lattice reducibility (Figures S14a vs. S15a), as well as (ii) more favorable DFT‐*E*_ads_ of −1.39 eV for acetone, that is markedly lower than −0.63 eV obtained for CO.

The electronic depth‐modulation of *ε*‐WO_3−x_ may be amplified by its incipient ferroelectricity [49], where surface polar distortions propagate electrostatic effects deeper into the lattice—potentially stabilizing lattice‐coupled charge carriers (e.g., polarons [88]) and enhancing subsurface charge accommodation. Such polaronic states also rationalize stronger acetone‐induced optical absorbance changes obtained during UV–Vis DRS (Figure 3d) [68]. Dipole moment calculations (Table S7) show that V_O_’s induce significantly larger polarization in *ε*‐ compared to *γ*‐WO_3−x_, consistent with a step‐like vacuum potential offset across the slab—indicative of an asymmetric electrostatic profile between top and bottom facets (Figure S23). This inherent asymmetric charge distribution may also enhance electrostatic interactions with polar analytes, such as acetone, compared to weakly polar species like CO, favoring stronger alignment and charge transfer across the polar *ε*‐WO_3−x_ surface. As a result, this deep Δ*ρ*(*z*) redistribution could enable carrier percolation through a wider crystallite network, effectively enhancing the transducer response. These polymorph‐specific features are corroborated by charge density difference (CDD) maps, which reveal extended electron accumulation around lattice O^2−^ deeper in the *ε*‐WO_3−x_ slab, accompanied by a corresponding depletion in the topmost atomic planes. Hence, while structural polymorphism does not impact the adsorption strength (*E*_ads_, Figure 3a) or net charge transfer, it determines how deeply the injected charge is accommodated—ultimately modulating conductivity (i.e., chemoresistive response), in agreement with our experimental observation (Figure 4a).

**FIGURE 5:**
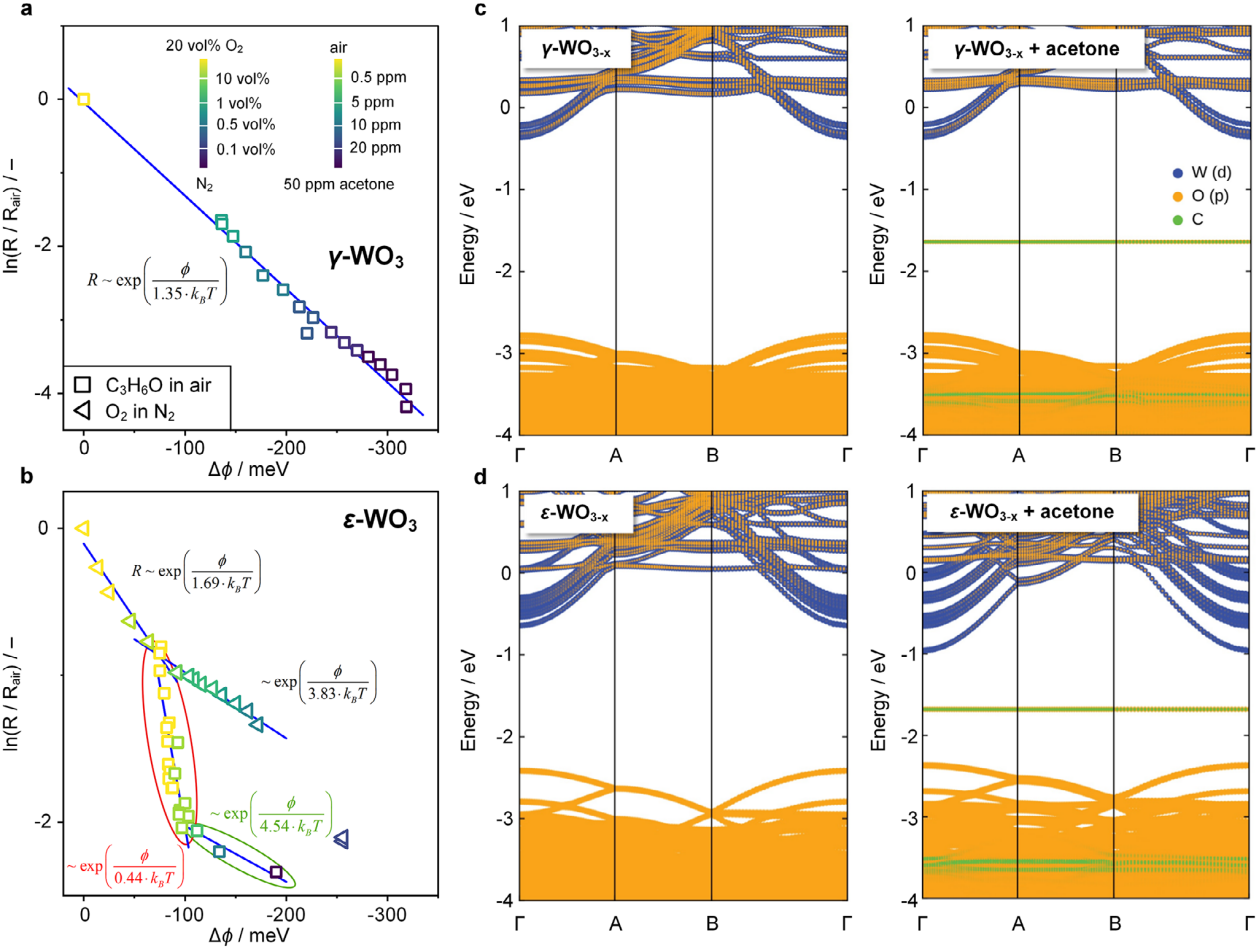
Transducer function and electronic band structure analysis. Operando electrophysical measurements (simultaneous *ϕ* and *R*) over (a) *γ*‐ and (b) *ε*‐WO_3_, under 0–20 vol% O_2_/N_2_ (triangles) and 0–50 ppm C_3_H_6_O/air (squares). Electronic band structure analysis of (c) *γ*‐ and (d) *ε*‐WO_3−x_ (001) before and after adsorption of an acetone molecule.

To further assess the transduction properties of *γ*‐ and *ε*‐WO_3_, we performed simultaneous work function (*φ*) and DC resistance measurements [89]. The results of our *operando φ* analysis (Figures S24–S29) are summarized in Figure 5a,b, showing the resistance vs. Δ*φ* (referenced to its value in dry synthetic air) attained in 0–20 vol% O_2_/N_2_ (triangles) and 0–50 ppm acetone/air (squares) mixtures. Note that the O_2_/N_2_ data for *γ*‐WO_3_ (Figure S24) were not included in Figure 5a as these do not follow a monotonic trend, and the *R* vs. Δ*φ* space is most frequently used [90] to visualize Arrhenius‐like (that is, thermally activated) transducer characteristics. As discussed in the Supporting Note S4 and Figures S30 and S31, such deviation for *γ*‐WO_3_ is attributed to molecularly adsorbed O_2_ species, O_2_(ad) [91, 92, 93, 94, 95, 96], opposed to (iono‐) sorbed O_β_^α−^.

Exposing *γ*‐WO_3_ (Figure 5a) to 0–50 ppm acetone yields *R* vs. *φ* points following an exponential trend with an inverse coefficient of 1.35 in Boltzmann's factor, consistent with electron‐depletion‐controlled transduction, as classically observed in SnO_2_‐based systems [97]. As shown in Figure 5b, *ε*‐WO_3_ exhibits a pronounced *φ* drop (∼80 meV) at 150 ppb acetone. However, at higher acetone concentrations, *φ* shifts become marginal and approach the noise floor, whereas resistance continues to drop—resulting in a steep, scattered regression line which systematically departs from the O_2_/N_2_ transduction characteristic.

In polycrystalline films of n‐type chemoresistive materials, resistance is usually directly modulated by back‐to‐back Schottky barriers formed at the grain boundaries between individual nanoparticles [92]. There, CB electrons are trapped at oxygen‐related surface acceptor states, leading to a less conductive, i.e., electron‐depleted, space charge layer due to the CB energy barrier height (*qV_S_*). Upon accepting electrons from a reducing agent into delocalized CB states above, for instance, the CB minimum (CBM), upward *E*_F_‐shifts (or, equivalently, downward *φ*‐shifts) are reflected in lower *qV_S_*, and, therefore, lower resistances.

Under typical sensing conditions (i.e., high‐oxygen backgrounds), Boltzmann's statistics are valid [89] and the resistance is pinned to *φ* through a simple relation: $R∼\exp\left(\frac{\phi }{m\cdot k_{B}T}\right)$, as observed for widely studied SnO_2_ in Figures S32–S35 (see also Supporting Note S5) and in agreement with the literature [98]. Therein, *m* is a fitting parameter and regarded as a measure of transduction efficiency, explaining [94], for instance, the lower sensitivity of p‐type (theoretical *m*  =  2) compared to n‐type (theoretical *m*  =  1) MO_x_. The value observed for our *ε*‐WO_3_ (Figure 5b, acetone/air mixtures) of 0.44 is far below the range expected for the *E*_F_‐pinned transduction regime, exhibiting a clear divergence in *R*‐*φ* characteristics that, to the best of our knowledge, is observed here for the first time. This is consistent with transduction enhancement from deep‐layer charge accommodation (Figure 4d) rather than classical band bending, and such seamless *φ*‐shifts in *ε*‐WO_3_ may originate from populating localized sub‐CBM states that do not appreciably shift *E*_F_ [99], being energetically decoupled from the CB edge.

To investigate this further, we evaluated the electronic band structures of *γ*‐ and *ε*‐WO_3−x_ before and after analyte introduction by DFT [100]. In *γ*‐WO_3−x_ (Figure 5c), no discernible change is observed in the vicinity of the CBM; the band edges remain unaltered, and no evident additional states emerge. In contrast, *ε*‐WO_3−x_ (Figure 5d) features the appearance of well‐defined electronic states just below the CBM following acetone adsorption. These states, predominantly of W(5d)‐character, are thermally accessible and could be populated under operating conditions [101]—providing an energetically favorable means to conductivity modulation [102]. To clarify their origin, the band structure was projected onto the W(5d) orbitals of all subsurface W atoms, revealing their dominant contribution to the states near the bottom of the CB (Figure S36a,b). Consistently, the spin‐density distribution and the local W─O bond distortions induced by acetone adsorption (Figure S36c) suggest that the excess electron density is distributed across several such subsurface atoms rather than being localized on a single W center. Together with the charge‐density‐difference analysis (Figure 4d), these observations suggest that acetone adsorption induces 2D delocalized electron polarons, predominantly associated with W(5d) orbitals near the CBM, which effectively modulate the conductivity of ε‐WO_3−x_. This behavior parallels similar effects observed on nanometal‐modified TiO_2_ surfaces (e.g., Ag_5_ or Cu_5_) [68, 103]. This orbital‐energy‐resolved distinction aligns with our hypothesis that the energetic allocation of transferred electrons—e.g., their proximity to the CBM—can be critical for chemoresistive response generation and needs to be considered next to conventional “total‐count” metrics such as net charge transfer. As a result, polymorphism in WO_3_ modulates chemoresistive sensing behavior primarily by shaping the density and accessibility of conduction‐relevant states.

## 3 Conclusion

This study delivers a mechanistic framework to investigate polymorph‐specific electronic transduction in semiconductive nanoparticulate films during molecular gas–solid interactions. It is applied to reveal the origin of a long‐standing observation in molecular sensing with WO_3_ polymorphs—namely, the enhanced response of the metastable *ε*‐ over the *γ*‐phase for acetone—whose origin had remained elusive despite widespread technological use. By combining in situ spectroscopy, thermochemical desorption, and *operando* work function measurements with first‐principles electronic structure calculations, we show that *γ*‐ and *ε*‐WO_3_ activate acetone similarly at the surface level via coordinatively unsaturated, electron‐deficient tungsten sites. The stronger chemoresistive response of *ε*‐WO_3_ is associated to stabilized W(5d)‐derived electronic states just below the conduction band minimum upon analyte adsorption. These states are thermally accessible and conduction‐relevant, offering an energetically favorable pathway for resistance modulation under operating conditions.

Together, these findings underscore that structural polymorphism does not dictate analyte affinity in WO_3_, but rather tunes the energetic landscape of electron accommodation, shifting the focus from classical charge‐transfer metrics to orbital‐level‐resolved transduction. This advance paves the way for rational transducer design strategies grounded in electronic structure criteria that is applicable and likely relevant also to other chemoresistive materials, closing an important gap in mechanistic modeling and understanding.

## 4 Methods

### 4.1 Nanoparticle Production

Nanoparticles of *γ*‐WO_3_ and *ε*‐WO_3_ were prepared by FSP, with a reactor design detailed elsewhere [104]. The *ε*‐WO_3_ phase was stabilized by Si addition (10 mol%) [36]. To prepare the precursor, we dissolved ammonium metatungstate hydrate (≥85% WO_3_ gravimetric basis, Sigma Aldrich, Switzerland) and hexamethyldisiloxane (Sigma Aldrich, Switzerland) in a 1:1 (by volume) mixture of ethanol and diethylene glycol monobutyl ether, to achieve a total molarity (W + Si) of 0.2 mol L^−1^. Thereafter, the precursor was fed through a capillary and dispersed by O_2_ (pressure drop of 1.6 bar) to form a fine spray. The precursor and dispersion flow rates were 5 mL min^−1^ and 5 L min^−1^, respectively. The spray was ignited and sustained by a pilot flame of premixed CH_4_ (1.2 L min^−1^, Methane 2.5, PanGas, Switzerland) and O_2_ (3.2 L min^−1^, Pangas, Switzerland). Additionally, 5 L min^−1^ O_2_ sheath flow was supplied to shield the flame and ensure excess oxidant. Gas flows were regulated by mass flow controllers (Bronkhorst, Netherlands), while the precursor solution flow was supplied by a syringe pump. The nanoparticles were deposited onto water‐cooled glass fiber filters (257 mm diameter, GF6, Hahnemühle Fineart, Germany) at a height above the burner of 55 cm aided by a vacuum pump (Seco SV 1025 C, Busch, Switzerland). The particles were carefully removed from the filter with a spatula and the obtained powders were sieved with a 250 µm stainless steel mesh. To fabricate sensors, particles were directly deposited from the aerosol [104] for 4 min at 20 cm height above the burner onto water‐cooled Al_2_O_3_ substrates, provided with interdigitated (*d* = 250 µm) Pt electrodes for resistance readout (electrode type #103, Electronic Design Center, Case Western University, USA). Both as‐produced nanoparticles and sensors were annealed in air at 500°C for 5 h (CWF 1300, Carbolite Gero, Germany).

### 4.2 Material Characterization

XRD patterns were measured with a Bruker D2 phaser (Bruker, USA) diffractometer equipped with a Cu anode, operated at 30 kV and 10 mA. The powder samples were loaded onto low‐background silicon holders and uniformly spread with the aid of a droplet of IPA. XRD patterns were recorded in Bragg‐Brentano geometry at 2θ(Cu Kα) between 20 and 70 degrees, with a step size of 0.020 degrees and a time per step of 15 s. Powder diffractograms were analyzed by Rietveld refinement as implemented in Topas 4.2 (Bruker) software, using the crystallographic information files of *γ*‐WO_3_ (ICSD 80056) and *ε*‐WO_3_ (ICSD 84139). Peak broadening due to crystallite size (*d*_XRD_) and microstrain [105] was modeled by Lorentzian and Gaussian contributions of a pseudo‐Voigt peak shape, respectively, while instrumental broadening was accounted for through the fundamental parameter approach [106].

N_2_‐physisorption isotherms (at 77 K) of powders (0.150 g) were recorded on a Tristar II Plus (Micromeritics, Germany). The specific surface area (SSA) was determined according to Brunauer‐Emmett‐Teller (BET) theory, at relative pressures between 0 and 0.4. Prior to measurement, the samples were degassed for 1.5 h at 120°C under N_2_ to remove water adsorbates.

UV–Vis DRS was carried out with a Cary 5000 UV–Vis‐NIR spectrophotometer (Agilent, USA). The powders were mixed with BaSO_4_ to achieve ∼10 wt%, and loaded in a high‐temperature cell (CaF_2_ windows) mounted in a Praying Mantis diffuse reflectance accessory (both Harrick Sci., USA). For in situ UV–Vis DRS transient analysis, the diffuse reflectance, (*F*(*R*_∞_)), of the sample was recorded at a fixed wavelength of 700 nm, using a spectral band width of 2 nm and an averaging time of 4 seconds. The temperature was set by a controller and measured by a K‐type thermocouple. Initially, powders were pre‐treated in dry synthetic air at 450°C for 90 min, and cooled down under N_2_ to 300°C. Therein, 980 ppm acetone (1000 ppm C_3_H_6_O in Ar, Pangas) was introduced for 60 min. Additional experiments in the (co‐)presence of acetone and O_2_ were performed. Acetone was admixed to a mildly oxidizing environment (0.4 vol% O_2_/N_2_) up to 980 ppm. The total flow through the UV–Vis cell was kept at 100 mL min^−1^.

The (optical) bandgap (*E*_g_) was estimated by acquiring diffuse reflectance spectra at room temperature between 200 and 800 nm, using Tauc's method [107, 108], i.e., Equation (1):

(1) $${\left(\alpha \cdot h\nu \right)}^{1/\gamma }=B\cdot \left(h\nu -E_{g}\right)$$

where γ  =  1/2 for direct electronic transitions and the absorption coefficient (*α*) is estimated with the Kubelka‐Munk (KM) function defined as in Equation (2):

(2) $$F\left(R_{\infty }\right)=\frac{{\left(1-R_{\infty }\right)}^{2}}{2R_{\infty }}$$

DRIFT spectroscopy was performed on a Vertex 70v spectrometer (Bruker, USA) equipped with a liquid‐nitrogen‐cooled mercury cadmium telluride (MCT) detector. Spectral acquisition was carried out in diffuse reflectance mode between 1000 and 4000 cm^−1^ at 2 cm^−1^ resolution and averaging 300 scans per spectrum. FT‐IR grade KBr (Sigma Aldrich Switzerland) was used as a background. Powder samples of γ‐ and ε‐WO_3_ were grinded together with KBr to achieve 10 wt.%, and loaded in a high‐temperature reaction cell (KBr windows) within a Praying Mantis diffuse reflectance accessory (Harrick Sci.). Therein, C_3_H_6_O (2 vol% in Ar, Pangas) was supplied by a rotameter with a needle valve (30 mL min^−1^) at temperatures between 50 C and 450°C. To follow the evolution of vibrational signatures, reflectance spectra were converted to pseudo‐absorbance spectra following literature recommendations [109] as in Equation (3):

(3) $$\mathrm{Pseudo}\mathrm{Absorbance}\left(R_{\infty }\right)=\log\left(\frac{1}{R_{\infty }}\right)$$

Temperature‐programmed experiments (heating rate of 10 K min^−1^) were carried out with an Autochem III chemisorption analyzer (Micromeritics, Germany) equipped with a vapor generator, a thermal conductivity detector (TCD), and connected to an *online* quadrupole mass spectrometer (Omnistar, Pfeiffer, Switzerland). About 70 mg of sample were loaded into a U‐shaped quartz reactor and pretreated in He at 300°C. TPR under CO (10 mol% in He), C_3_H_6_O (1000 ppm in Ar), and H_2_ (5 vol% in Ar, all Pangas) were recorded between 30 C and 1000°C, and were followed by TPO under 5 vol% O_2_ in He (Pangas). TPD runs were performed in He after adsorbing O_2_, CO_2_ (10 vol% in Ar), C_3_H_6_O, and Py, the latter generated as a vapor at a reflux temperature of 45°C.

For HR‐TEM, the material was dispersed in ethanol and a few drops of the suspension were deposited onto a perforated carbon foil supported on a copper grid. After solvent evaporation, the grid was mounted on the single tilt holder of the microscope. TEM investigations were performed on a JEOL JEM F300 (GrandARM) with a cold field emission gun operated at 300 kV.

XAFS of the W L_3_‐edge (10.207 keV) was carried out at the MAX IV (Lund, Sweden), using a Si(111) double crystal monochromator and ion chambers to record *I*_0_ and *I*_t_ signals. Samples were directly pressed into 13‐mm‐diameter pellets after mixing with boron nitride to optimize the transmission measurement. The data were processed using the Demeter software package [110] (including Athena and Artemis). Athena was used for unit‐*E*_0_‐normalization and background removal to extract the *k* ^2^‐weighted *χ*(*k*) signal. Artemis was used to fit the EXAFS data in real space between 2.5 Å^−1^ < *k* < 13.0 Å^−1^ and 1.0 Å < *R* < 2.1 Å with the multiple *k*_w_ method [111]. The amplitude reduction factor *S*_0_^2^ from the EXAFS analysis of a W foil was 0.868, which was used as a fixed parameter for EXAFS fitting. The coordination numbers and bond lengths were calculated based on the reported structures from the Inorganic Crystal Structure Database (ICSD) as indicated in the text.

### 4.3 Chemoresistive Characterization

The sensors were mounted onto MACOR holders and placed in a PTFE‐made chamber [112]. The sensing film was heated by applying a constant voltage to a meander‐shaped Pt heater in the back of the substrate. The temperature was determined with a multimeter (2700, Keithley, USA) by using the same Pt heater as the resistance temperature detector. The chamber was connected to a gas mixing set‐up. Hydrocarbon‐free synthetic air (Pangas, C_n_H_m_, and NO_x_ < 100 ppb) was used as a carrier gas and the analytes from certified gas standards were admixed by mass flow controllers (Bronkhorst, Netherlands) to obtain the desired gas mixture composition. The calibrated and certified gas standards (all Pangas, dry synthetic air as carrier) used were C_2_H_6_O (15 ppm), CO (506 ppm), and C_3_H_6_O (18 and 150 ppm), while the total flow was set constant to 300 mL min^−1^. The DC resistance of the sensing film was measured continuously between the interdigitated Pt electrodes with a Keithley 2700 multimeter and a Keithley DMM7510 picoammeter for *γ*‐ and *ε*‐WO_3_, respectively. The chemoresistive response (*S*) was defined in Equation (4) as the normalized resistance variation:

(4) $$S=\frac{R_{a}-R_{g}}{R_{g}}$$

where *R*_a_ and *R*_g_ are the resistances of the sensing film under clean air and gas exposure, respectively.

### 4.4 Operando Work Function Analysis

*Operando* work function measurements were performed by the Kelvin oscillator method [113] (single point KP020, KP Technology, UK). The sensors were mounted onto the same MACOR holders and placed in an aluminum chamber, with a 4‐mm hole drilling to allow the Au tip (2‐mm diameter) of the Kelvin probe to approach the sample at working distances of about 0.5 mm, which was enabled by the gradient function of the control software. The ohmic resistance was simultaneously monitored by applying a probing voltage between 0.5 and 10 V and measuring the induced current (2400, Keithley). Prior to that, IV sweeps were recorded at operational conditions to ensure ohmic behavior. The aluminum chamber, a lead of the Pt heater, as well as a lead of the chemoresistive film were connected and equipotential with the Kelvin probe. To control the sample gas environment, the accessory was installed in a glovebox, gases were supplied by the same certified standards described above, with the addition of pure N_2_ (purity 5.0, Pangas) and H_2_ (47.9 ppm in dry synthetic air, Pangas), and mixed with mass flow controllers (Brooks Instrument, USA). The total flow was kept at 300 mL min^−1^.

### 4.5 DFT Calculations

DFT calculations were carried out with the Vienna Ab initio Simulation Package (VASP) [114]. To account for the interaction between ion cores and valence electrons, the projector augmented wave (PAW) method was employed [115]. Electron exchange‐correlation interactions were computed using the generalized gradient approximation (GGA) with the Perdew‐Burke‐Ernzerhof (PBE) functional [116], along with the Grimme D3 dispersion correction [117]. The cutoff energy for the plane wave expansion was set to 450 eV after convergence testing. For geometry optimization, the conjugate gradient algorithm was used to relax atomic positions until the total force on each ion was smaller than 0.02 eV Å^−1^, while the convergence criterion for the electronic self‐consistency cycle was set to 10^−6^ eV. The electronic properties of the relaxed structure were calculated using the DFT‐½ method, adding to only oxygen atoms as in the previous literature [26]. To facilitate a more accurate comparison between WO_3_ polymorphs, the *k*‐point mesh density was kept consistent rather than maintaining an identical number of *k*‐points, owing to differences in unit cell dimensions. Consequently, a Monkhorst‐Pack grid of 7 × 7 × 6 was utilized for the *γ*‐phase, while a denser grid of 9 × 10 × 7 was employed for the *ε*‐phase, ensuring uniform sampling of the Brillouin zone.

Based on the relaxed bulk structures, slab models of *γ*‐ and *ε*‐WO_3_ surfaces were extracted by cleaving along the (001) direction [73, 74]. For *γ*‐WO_3_, a c(2 × 2) reconstruction of the (001) surface was adopted, which is the most commonly used model in *γ*‐WO_3_ simulations [118]. These slab models consist of six atomic layers, containing 48 W atoms and 144 O atoms, with the top and bottom layers containing each half of the oxygen atoms. All six atomic layers were fully relaxed during structural optimization to effectively eliminate mid‐gap states, thereby avoiding the need for hydrogen termination on the bottom layer and preventing artifacts caused by geometry distortions from fixed bottom layers [19]. To ensure comparability between phases, a similar 2 × 2 supercell model with six layers was constructed for *ε*‐WO_3_ (001), comprising also 48 W atoms and 144 O atoms. Besides, a vacuum thickness of ∼18 Å was introduced to prevent inter‐slab interactions given that the calculations were performed under periodic boundary conditions. Spin polarization and dipole moment corrections were consistently applied throughout the calculations. Bulk and surface V_O_’s were introduced as described in the text (section 2.2), and their formation energies in both polymorphs were computed by Equation (5):

(5) $$E_{formation}\left(V_{O}\right)=E_{defective}-E_{pristine}+\frac{1}{2}E_{O_{2}}$$

Further, the adsorption energy per molecule was calculated through the following equation:

(6) $$E_{ads}=E_{substrate+adsorbate}-\left(E_{substrate}+E_{adsorbate}\right)$$

AIMD was performed using the canonical (NVT) ensemble with the Nosé‐Hoover thermostat (*T* = 500 K). Acetone dynamics on the surface of WO_3−x_ were simulated for 10 ps with a time step of 1 fs.

### 4.6 Statistical Analysis

Measurements done under the same conditions were reported as mean ± standard deviation. The response vs. concentration scatter data were fitted with a straight line on a log‐log scale using Origin (OriginLab, USA). Similarly, linear fits on lin‐log space were obtained from *operando* work function R vs. ϕ plots to obtain transduction efficiency, and from Tauc plots to estimate optical *E*_g_. To evaluate the agreement between the data and the fitted result, the coefficient of determination (*R* ^2^) was calculated. Crystal phase quantification was carried out with Rietveld's method using the software Topas 4.2 (Bruker). The quality of the X‐ray diffraction structural refinements was assessed from the weighted residual (*R*_wp_) factor, which was kept below 7%. The fit quality of W‐L_3_ EXAFS is determined from its residual *R*_p_.

## Author Contributions

M.D. and A.T.G. conceived the research and designed the experiments. M.D., M.Y., and A.T.G. coordinated the study. M.Y., V.G.M., Y.C., and K.S. developed the computational framework. M.D. and S.N. were primarily responsible for experimental data collection and analysis. M.Y. analyzed the structures obtained from DFT calculations. A.T.G. supervised the project and was responsible for funding acquisition. M.D. and M.Y. prepared the figures and wrote the original draft. All authors supported the revision of the manuscript and gave final approval.

## Funding

This study was financially supported by the Innosuisse (Innovation project 109.063 IP‐LS), Swiss State Secretariat for Education, Research, and Innovation (SERI) under contract number MB22.00041 (ERC‐STG‐21 “HEALTHSENSE”), the Swiss National Science Foundation (BRIDGE Discovery grant #218650) and JSPS KAKENHI (Grant number 23KJ0196).

## Conflicts of Interest

The authors declare no conflicts of interest.

## Supporting information




**Supporting File**: adma72927‐sup‐0001‐SuppMat.docx.

**Supporting File**: adma72927‐sup‐0001‐MovieS1.mp4.

## Data Availability

The data that supports the findings of this study is available from the authors upon request.
