# Supplementary material for: Polymorph‐Specific Electronic Transduction in WO3 during Molecular Sensing
Source: Adv Mater. 2026 Mar 28;38(43):e16840. doi: 10.1002/adma.202516840 (PMC13432034; doi:10.1002/adma.202516840)
Supplement: Supplementary file 1 — Supporting File: adma72927‐sup‐0001‐SuppMat.docx. [file ADMA-38-e16840-s002.docx]

# Supporting Information

Polymorph-Specific Electronic Transduction in WO_3_ during Molecular Sensing

*Matteo D’Andria^†, 1^, Meng Yin^†, 2^, Stefan Neuhauser^1^, Vlasis G. Mavrantzas^3,4^, Ying Chen^5^, Ken Suzuki^6^, and Andreas T. Güntner^1,^**

M. D’Andria, S. Neuhauser, A.T. Güntner

Human-centered Sensing Laboratory, Department of Mechanical and Process Engineering, ETH Zurich, CH-8092 Zurich, Switzerland
E-mail: andregue@ethz.ch

M. Yin

Department of Finemechanics, Graduate School of Engineering, Tohoku University, Sendai, Miyagi 9808579, Japan

V.G. Mavrantzas

Macromolecular Engineering Laboratory, Department of Mechanical and Process Engineering, ETH Zurich, CH-8092 Zurich, Switzerland

Department of Chemical Engineering, University of Patras & Institute of Chemical Engineering Sciences (ICE-HT/FORTH), Patras, GR 26504, Greece

Y. Chen

Global Learning Center, Tohoku University, Sendai, Miyagi 9808576, Japan

K. Suzuki

Green X-tech Center, Green Goals Initiative, Tohoku University, Sendai, Miyagi 9808579, Japan

† These authors contributed equally to this work as co-first authors.

## Supplementary Note S1

To complement the DRIFTS analysis in Figure 3b, we conducted AIMD simulations of acetone adsorbed near a surface V_O_ on *γ*-WO_3-x_ (001) at 500 K (Figure S9 and Figure S10, Supplementary Movie 1) and further DRIFTS under more representative conditions (Figure S11). The trajectory confirms that acetone remains stably bound over 10 ps, supporting the thermal robustness of the adsorption complex under sensing-relevant conditions. The C=O bond length $\left( d_{C-O} \right)$ fluctuates dynamically as shown in Figure S10, with transient elongations beyond 1.30 Å (Figure S10), indicative of vibrational softening and C=O activation $\left( d_{C-O}^{initial}=1.246 Å \right)$. Beyond C=O dynamics, AIMD also reveals pronounced torsional motion of the methyl groups, particularly in the form of hindered rotations and out-of-plane bending, likely contributing to the broader spectral *δ*(CH_3_)-shoulder near 1380 cm^-1^.

Additional DRIFTS measurements are shown in Figure S11 for (a) γ- and (b) ε-WO_3_ in the (co-)presence of O_2_ (0.8 vol% and 10 vol%) and lower acetone concentrations (<1000 ppm) during sequential treatments at different temperatures and gas compositions. Adsorbed carbonyl-related peaks are observed in the ~1700 cm^-1^ region, while features around 1600 and 1400 cm^-1^ are present that are assigned to oxygenate/enolate and carboxylate species, respectively. Under these conditions, the intensities of broad bands between 2100–2000 and 1900–1800 cm^-1^ are modulated under reducing/oxidizing atmospheres. These (gray-shaded) are attributed to overtone transitions lattice W–O^46^ that highlights the relevance of lattice-oxygen–enabled (MvK) chemistry for WO_3_ sensors. Importantly, increasing the temperature from 300 °C to 400 °C (Figure S11a) leads to the disappearance of carbonyl contributions, consistent with the C=O softening observed by AIMD and in Figure 3b.

## Supplementary Note S2

To assess lattice reducibility under acetone exposure, we monitored the evolution of KM function at 700 nm, attributed to the *d*-*d* transitions^[84]^ of reduced W^(6-^*^δ^*^)+^ species. Both polymorphs exhibit progressive increase in signal intensity, reflecting electron transfer to coordinatively unsaturated tungsten, in agreement with the Bader charge redistribution. Therein, ε-WO_3_ features larger increase in KM than the other polymorph (Figure 3d), suggesting a stronger modulation of reduced W(d) states. Faster UV-Vis–derived transients in ε-WO_3_ are attributed to subsurface W(d) polaron-like states formed upon acetone–surface interaction, as discussed in section 2.4. Note that these apparent kinetics are obtained under 980 ppm C_3_H_6_O (see *Methods*) and in the absence of O_2_ (i.e., inert background) according to the literature.”

NH_3_-TPD profiles (Figure 3e) indicate the presence of such Lewis-acidic W sites via *m*/*z* = 17 desorption that are uncorrelated with and exceeding signal intensity of *m*/*z* = 18,^[85]^ excluding simple H_2_O evolution. Py chemisorption (Figure S13) further confirms the availability of these Lewis sites, with both polymorphs retaining Py up to 350 °C. Thereby, *ε*-WO_3_ exhibits a slightly higher desorption onset, indicating comparable but marginally stronger acid-base interactions.

Following acetone-reduction, O_2_-TPO (Figure 3f,g) reveals a significant O_2_-uptake between 400 – 550 °C for both polymorphs. Subsequent CO_2_ evolution at higher temperatures reflects the oxidative removal of residual carbon species, indicating that acetone-derived intermediates remained on the surface and required replenished V_O_ to be fully reacted. Under CO- and H_2_-TPR conditions (Figure S14), V_O_ formation after lattice oxygen consumption occurs at rather high temperatures (> 500 °C). In contrast, C_3_H_6_O-TPR (Figure S15) features measurable redox activity near 400 °C, accompanied by the formation of surface-bound intermediates (e.g., enolates, formates, condensation products) that may undergo deep dehydrogenation at higher temperatures, as evidenced by the pronounced H_2_ release (*m*/*z* = 2) above 600 °C. This lower reducibility aligns with the characteristically weak CO and H_2_ responses observed^[46]^ in WO_3_-based chemoresistors. Finally, *m*/*z* = 44 evolution during CO_2_-TPD (Figure S16) shows lower intensity and a distinct thermal onset compared to Figure 3c, indicating^[77]^ that acetone more readily mobilizes lattice oxygen — in agreement with the MvK-type behavior evidenced across Figure 3c–g.

## Supplementary Note S3

To rationalize the optimum temperature of 330 °C for both WO_3_ polymorphs, we performed additional DRIFT spectroscopy under oxygen-containing backgrounds. We observe that the carbonyl-associated band(s) in the ~1700 cm^-1^ region progressively diminish with increasing temperature, with the most pronounced attenuation occurring up to approx. 300 °C, after which the carbonyl contribution vanishes (Figure S19), or stops changing significantly (Figure 3b, higher acetone concentrations), a trend that is consistent for both polymorphs. Therefore, we identify the progressive loss of ν(C=O) intensity as a spectroscopic signature that indicates acetone transition from early-activation states at lower temperature to more strongly activated oxygenate chemistry at higher temperature, and propose that this is the origin of optimum-shaped response vs. temperature behavior.

Further, to explore whether lattice redox follows a similarly non-monotonic temperature dependence, we evaluated the W–O lattice overtone/combination region^[46]^ using the pseudo-absorbance spectra from Figure S20 referenced to the acetone-free baseline (at 50 °C):

$$\text{Relative pseudo-absorbance }=\log\left(\frac{R_{\text{reference}}}{R}\right)$$

Therein, the W–O^(1)^ relative intensity exhibits an inverted bell shape, consistent with an operating regime where analyte-driven reduction (V_O_ formation) and re-oxidation (V_O_ consumption) are favorably balanced. These observations support that the sensor optimum around 330 °C corresponds to a “sweet spot” where acetone activation is sufficiently advanced – i.e., ν(C=O) is strongly attenuated – while conductivity modulations are not yet suppressed by excessively fast oxidative turnover at higher temperatures.

## Supplementary Note S4

The absence of monotonic increases in both *φ* and *R* with rising O_2_ concentration (Figure S24) is attributed to the dominant role of molecularly adsorbed O_2_, i.e., O_2_(ad), on the surface of *γ*-WO_3_. By contrast, *ε*-WO_3_ displays a concurrent rise in both electrophysical parameters (Figure S27), yielding a piecewise linear transduction behavior (on a semi-log scale, Figure 5b) consistent with *operando* *φ* measurements. In fact, such O_2_(ad) species are more abundant on *γ*- rather than on *ε*-WO_3_ as supported by stronger binding (lower *E*_ads_(O_2_), Figure S30) and more pronounced low-temperature O_2_ desorption^[91]^ (Figure S31). Unlike strongly (iono-)sorbed^[92]^ O_β_^α–^ that are also present on *γ*-WO_3_ (monotonic *R*-increase in Figure S24), O_2_(ad) do not withdraw CB electrons and therefore do not contribute to build up a delocalized space-charge width.^[93]^ Instead, they can form localized dipoles that alter *φ* via electron affinity (*χ*) shifts,^[93-95]^ partially counteracting—i.e., δ^–^ charge closer to the surface^[96]^—the upwards O_β_^α–^-related band bending (*qV*_s_).

## Supplementary Note S5

The SnO_2_ precursor was prepared by dissolving tin(II) 2-ethylhexanoate (90 wt% in 2-ethylhexanoic acid, Sigma Aldrich) in xylene (ACS Reagent, Sigma Aldrich) to obtain a Sn molarity of 0.2 mol L^-1^. The precursor was fed to a spray flame with the same process parameters as for *γ*- and *ε*-WO_3_, and SnO_2_ sensors were similarly obtained by direct deposition (4 minutes) from the aerosol onto identical sensor substrates positioned at 20 cm above the nozzle. After annealing in air (5 hours at 500 °C), they were mounted onto MACOR^®^ holders and measured in our *operando* work function (*φ*) setup (see Methods).

Figures S26 and S27 show simultaneous *φ* and resistance traces of SnO_2_ operated at 400 °C during exposure to 8 – 175 ppm CO and 0.5 – 16.5 ppm H_2_ in dry air. In both cases, the measured resistance falls between its values in synthetic air and N_2_, consistent with operation under an electron-depletion regime,^[97]^ as expected for SnO_2_ in high-oxygen (~ 20 vol%) backgrounds. As shown in Figure S35, the *R* vs. *φ* data for H_2_/air (green) and CO/air (red) collapse onto a single regression line with an inverse coefficient of 1.19, in excellent agreement with the literature.^[98]^ Notably, this value is close to that obtained under O_2_/N_2_ at 300 °C (inverse coefficient of 1.27, Figure S34), validating our *operando* methodology. At 400 °C, however, O_2_/N_2_ exposures may have led to electron affinity (*χ*) shifts (thus, they are not presented), similar to those discussed for *γ*-WO_3_ (see Supplementary Note S4 and Figure S24), reflecting temperature-dependent surface dipole contributions.^[93]^

## Supplementary Movie 1

The AIMD run at 500 K is provided as an *mp4 Video file.

**
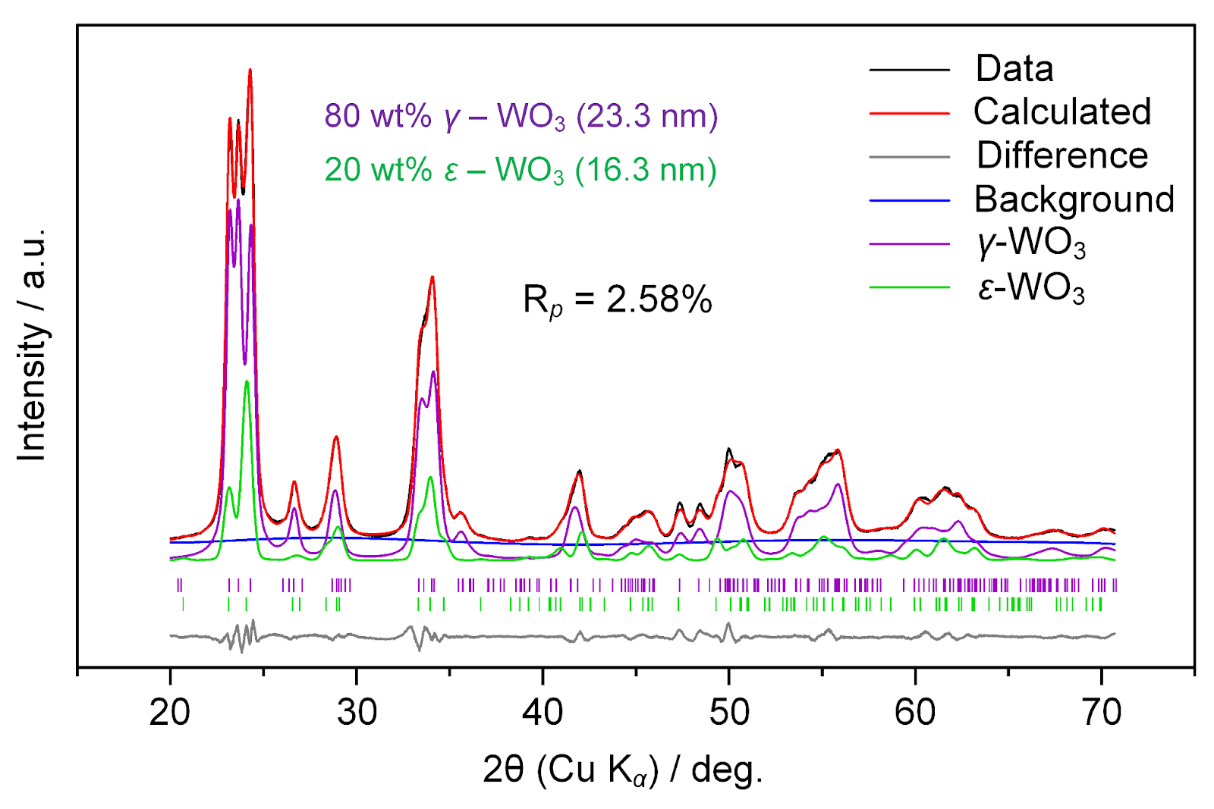
**

**Figure S1:** XRD refinement of pure WO_3_ nanocrystals accounting for the presence of *ε*-WO_3_.

**
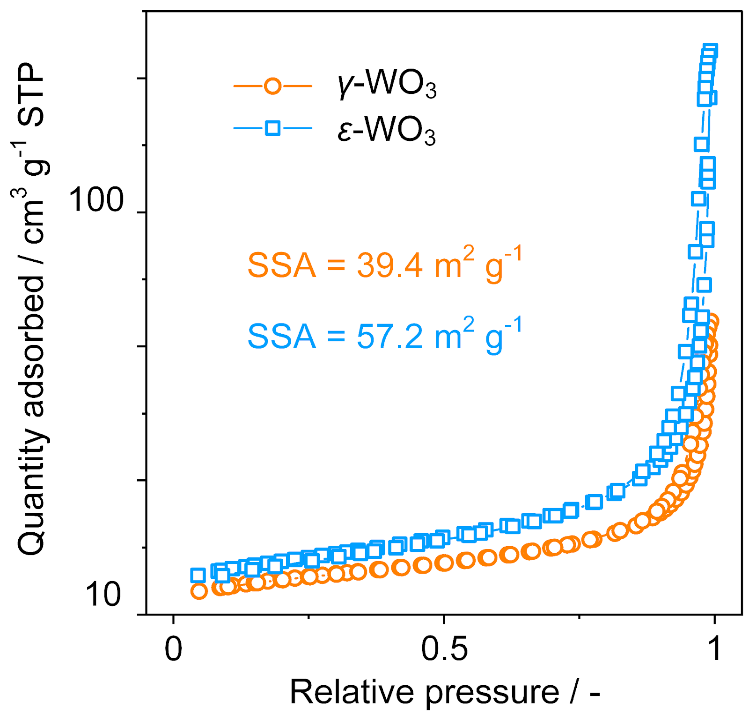
**

**Figure S2:** N_2_-adsorption isotherm of *γ*-WO_3_ and *ε*-WO_3_.


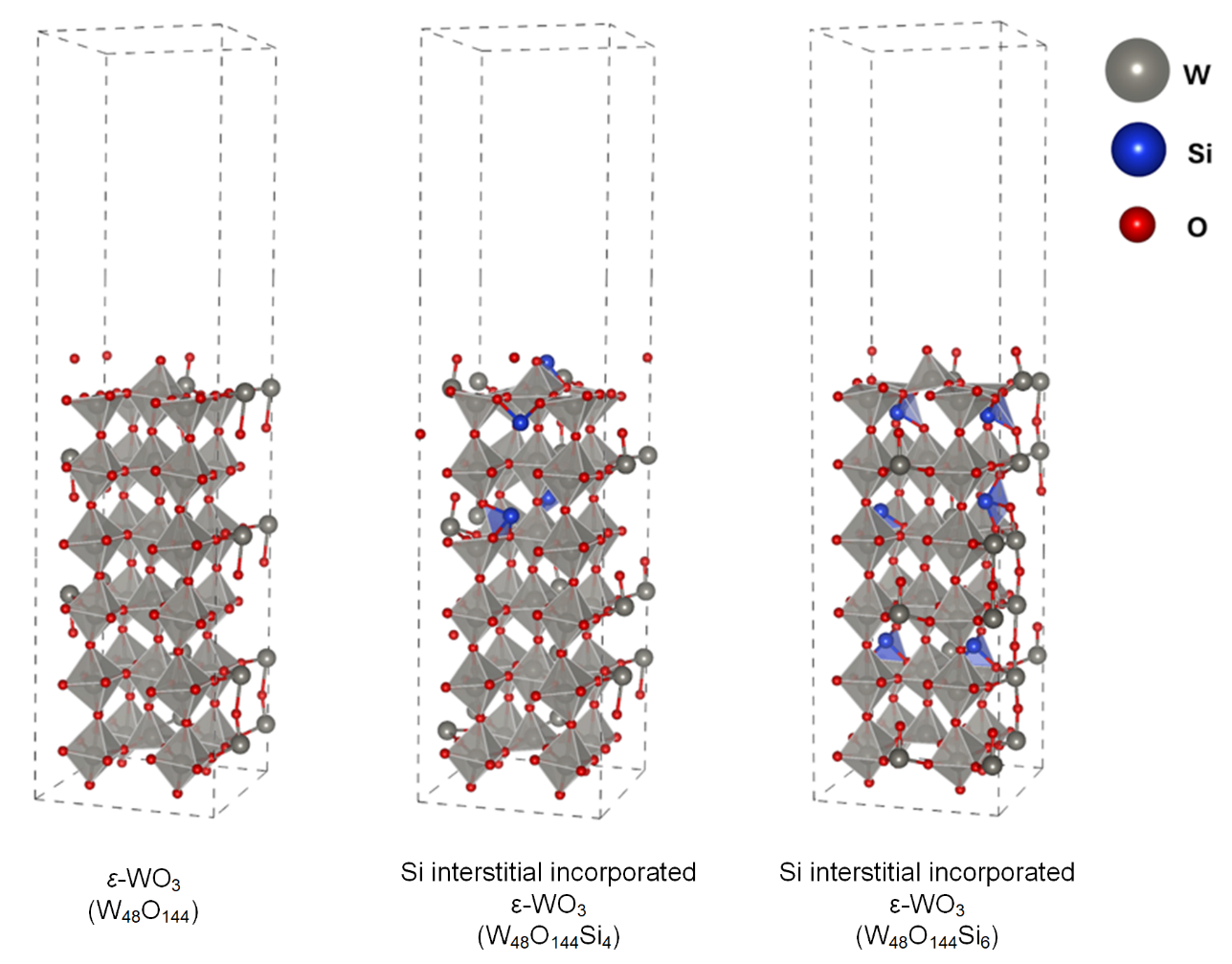


**Figure S3:** DFT calculated pure and interstitial Si-incorporated *ε*-WO_3_ (001) at varying concentrations.

**
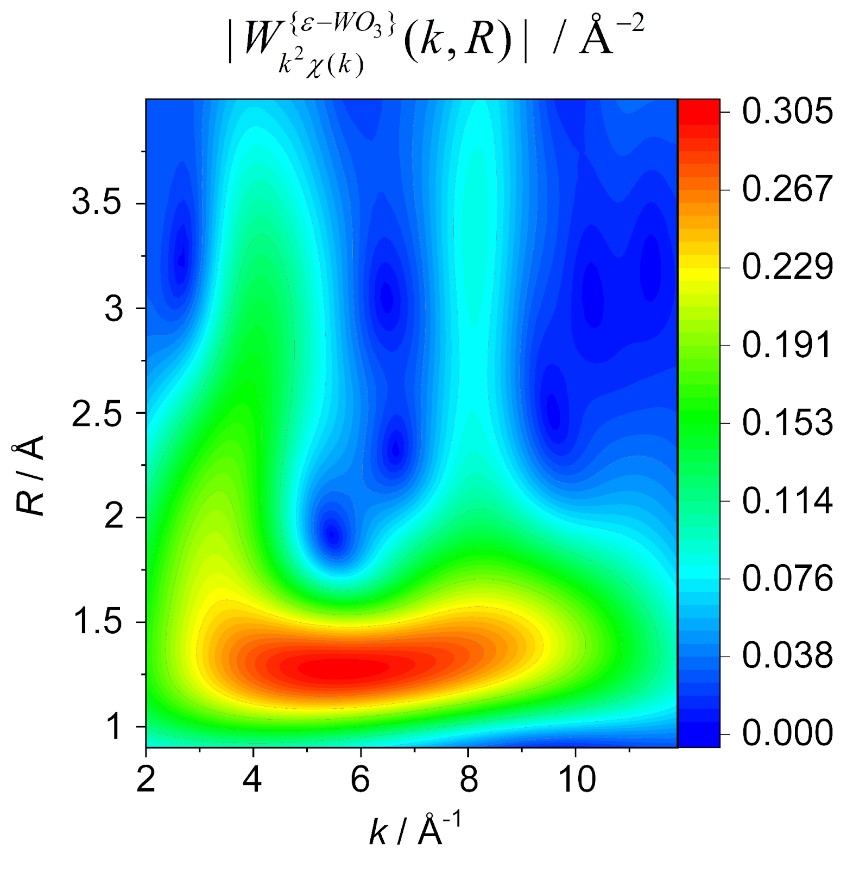
**

**Figure S4:** Magnitude of wavelet transform of $k^{2}\chi\left( k \right)$ at the W-L_3_ edge for *ε*-WO_3_.

**
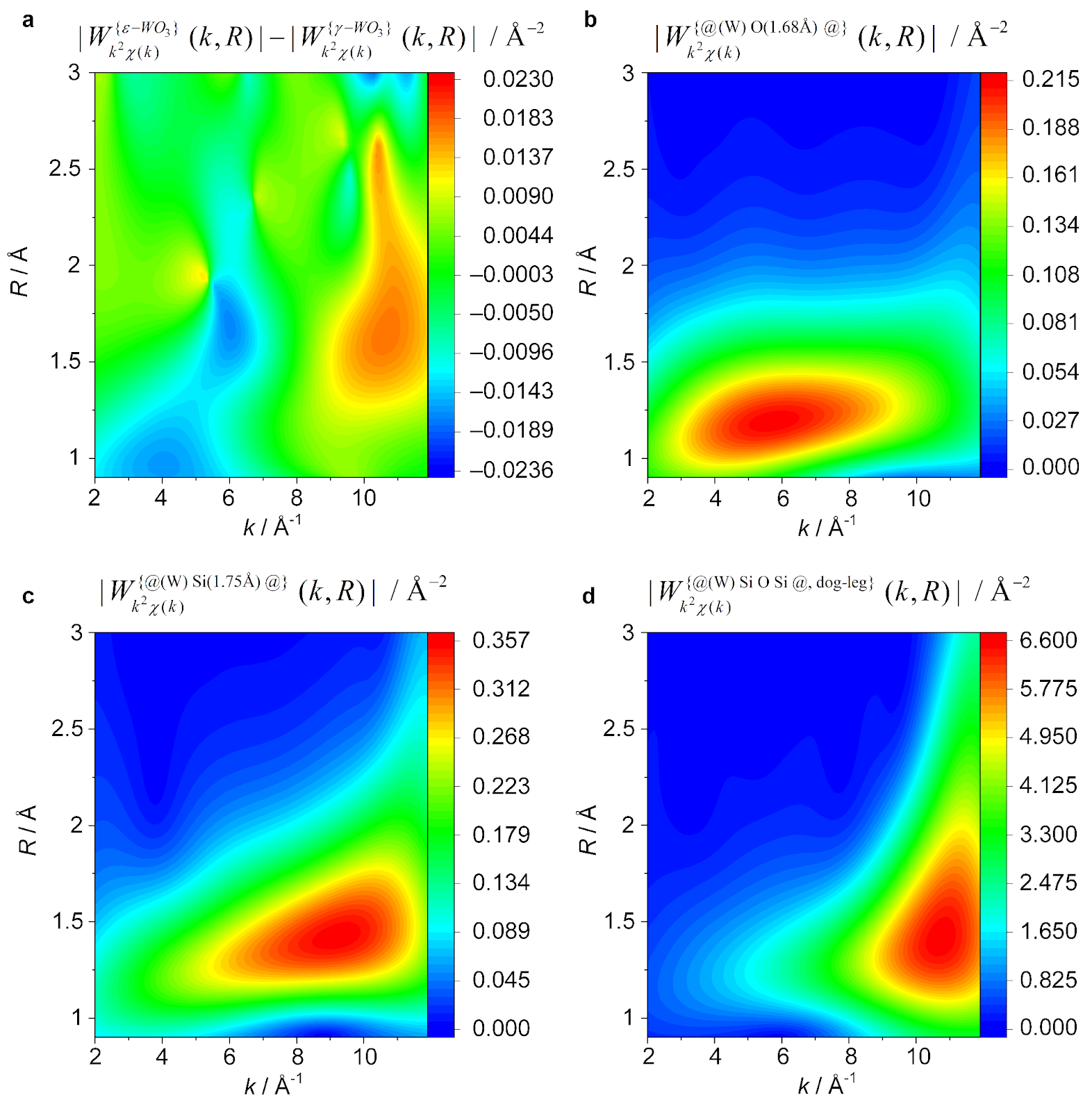
**

**Figure S5:** (a) Wavelet-transform difference map between *ε*-WO_3_ and *γ*-WO_3_ samples. Theoretical magnitudes of the wavelet transform of $k^{2}\chi(k)$ from specific scattering paths: (b) single W – O scattering, (c) single W – Si scattering as well as (d) multiple W – Si – O – Si, “dogleg”-like, scattering. All wavelet transforms were computed for *k* and *R* between 2 – 12 Å^-1^ and 0.9 – 4 Å, respectively, with the same Morlet’s wavelet parameters (*η* = 5 and *σ* = 1).


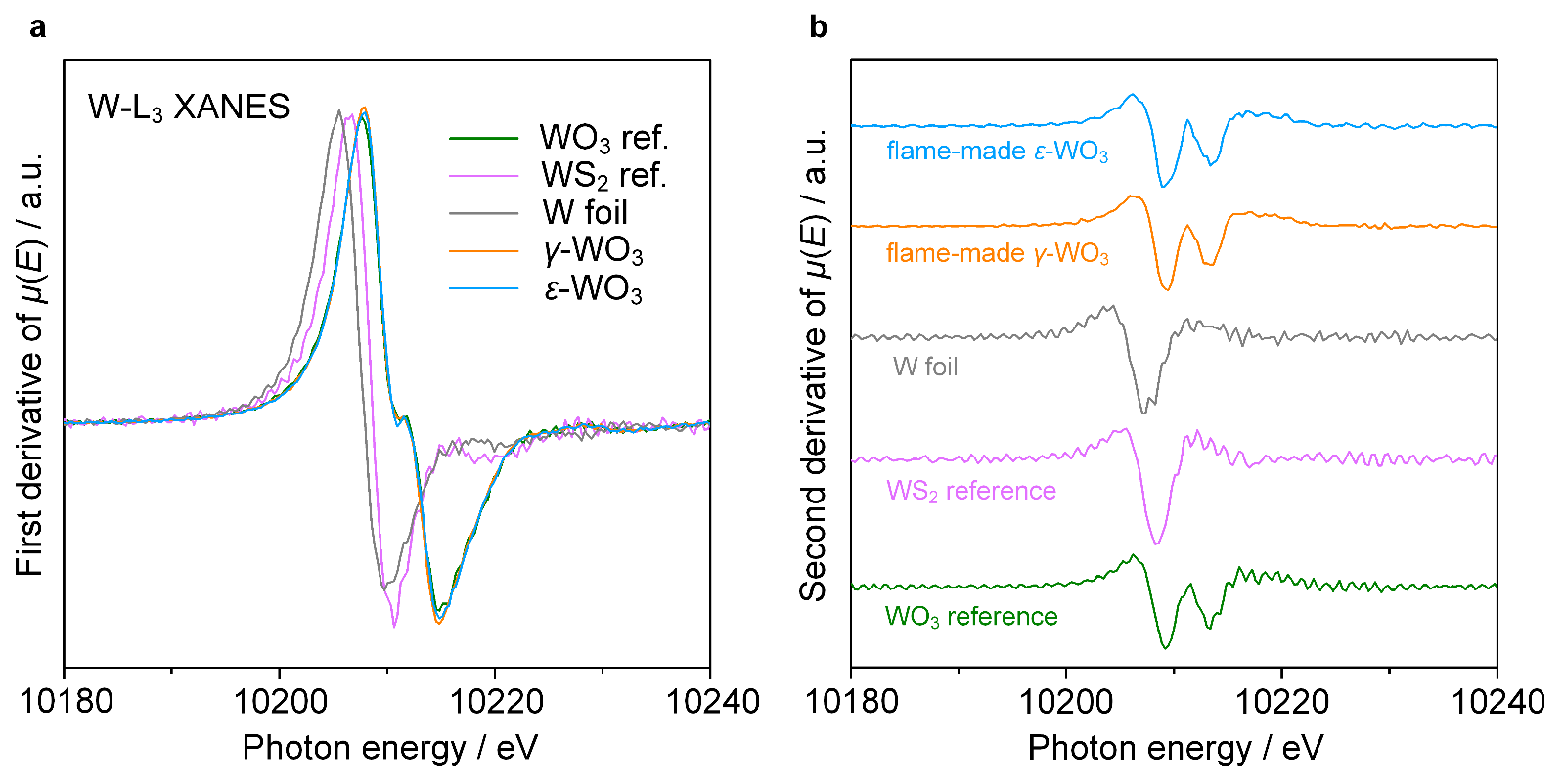


**Figure S6:** (a) First derivative of the W-L_3_ XANES for *γ*-WO_3_, *ε*-WO_3_, as well as for the reference WO_3_, WS_2_, and the W foil.


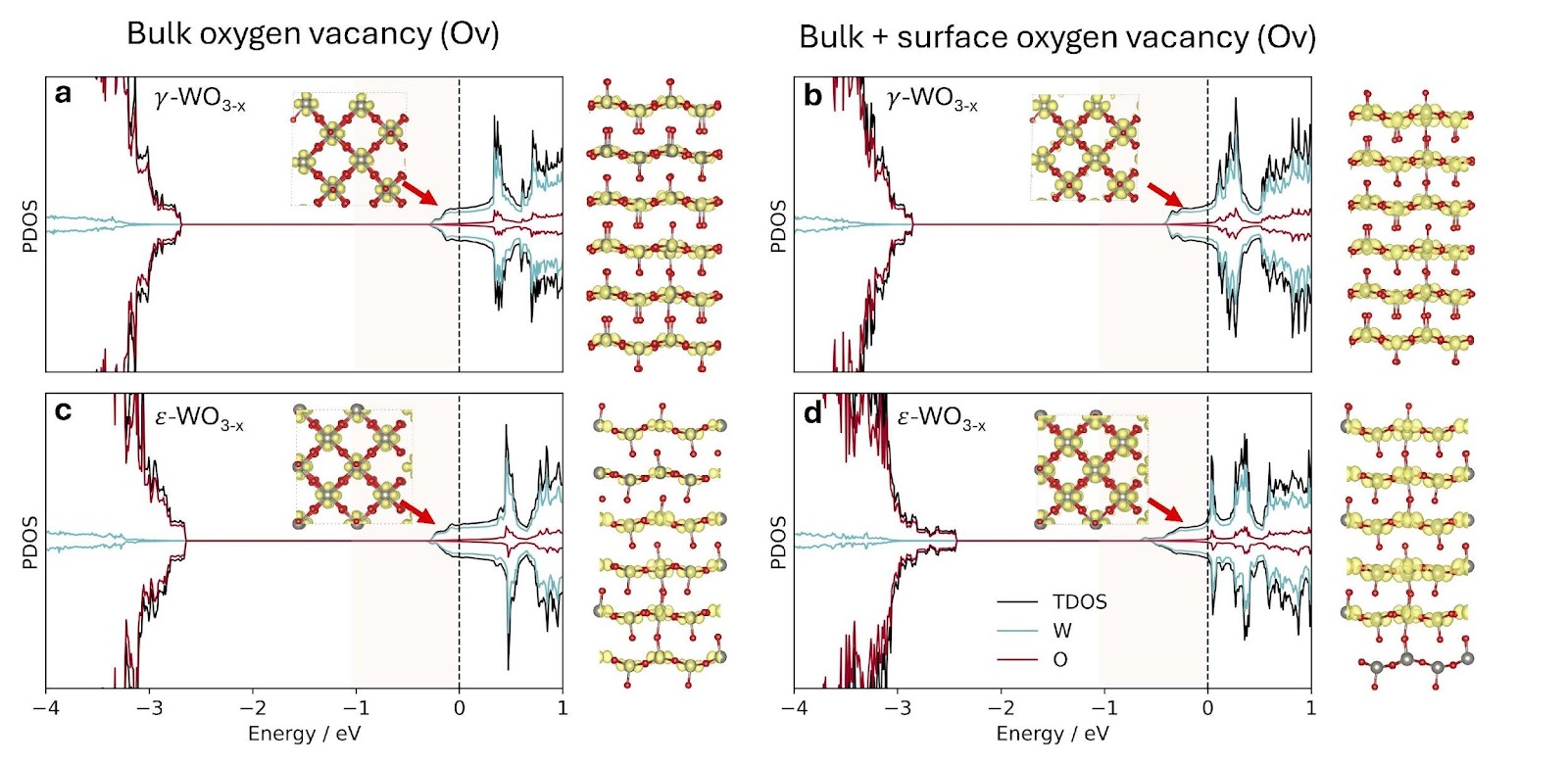


**Figure S7:** Projected density of states (PDOS) and partial charge density: the inset and right plots in each figure illustrate the electron density within 1 eV below *E*_F_, with an isosurface level of $0.001 e\cdotÅ^{-3}$.


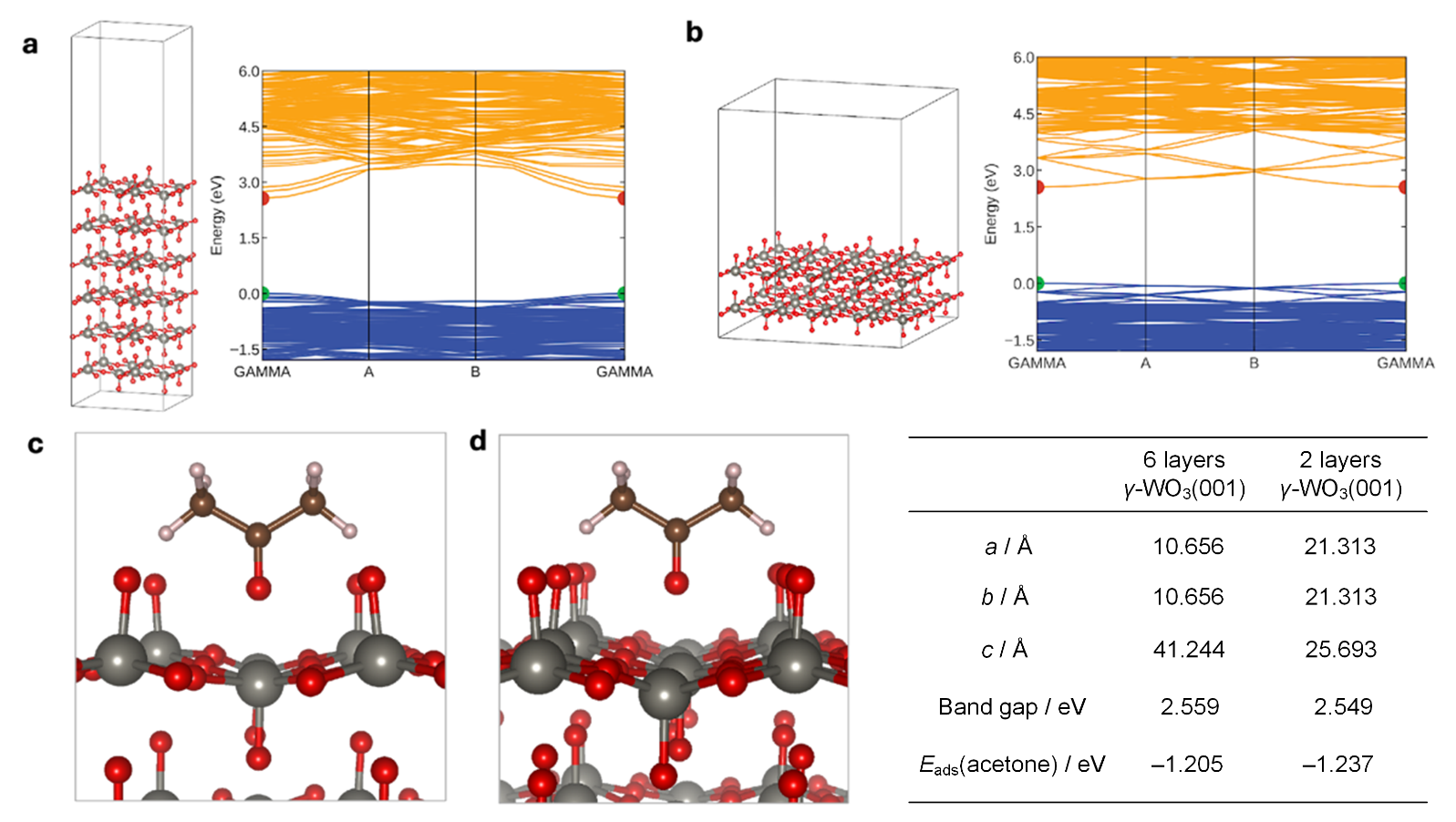


**Figure S8:** Size effects on WO_3_’s electronic properties and acetone adsorption: (a) 6-layer *γ*-WO_3_ (001) and its band structure. (b) 2-layer *γ*-WO_3_ (001) and its band structure. (c-d) Optimal acetone adsorption structures on 6- and 2-layer *γ*-WO_3_ (001). The accompanying table summarizes calculation details, including lattice constants, band gaps, and acetone adsorption energies (*E*_ads_).


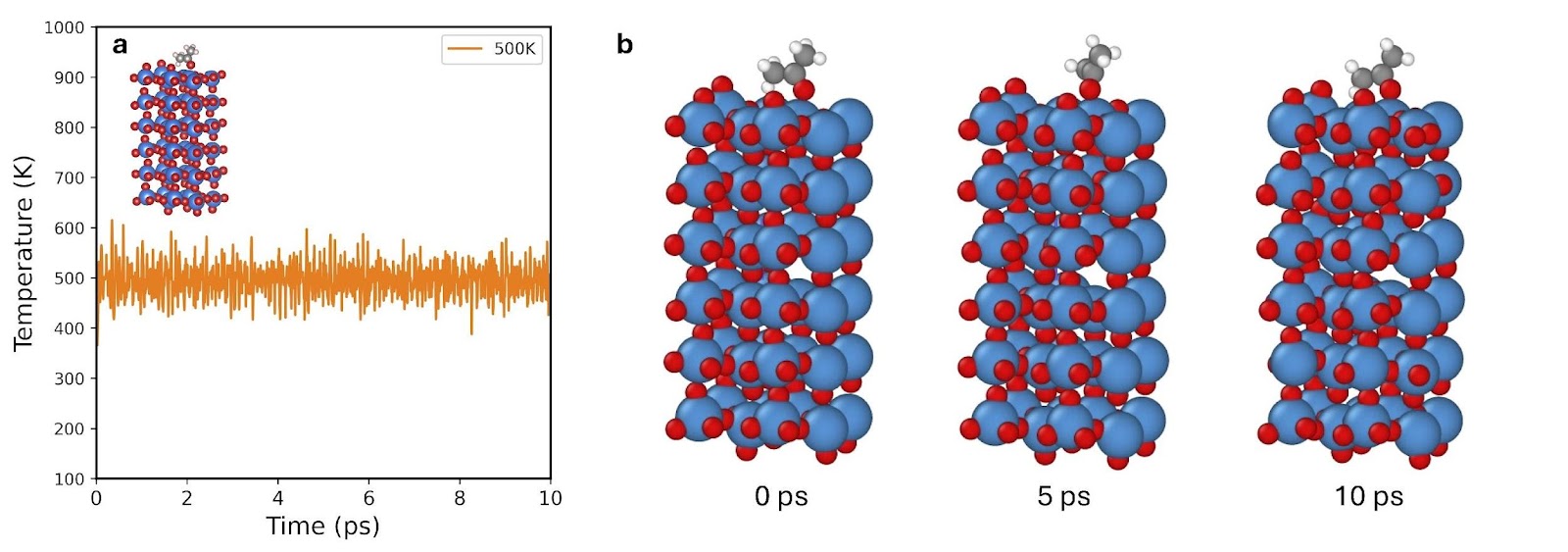


**Figure S9:** AIMD simulation of *γ*-WO_3_ (001) + acetone at 500 K: (a) Temperature stabilization over 10 ps. (b) Snapshots of the acetone-adsorbed structure at 0, 5, and 10 ps.


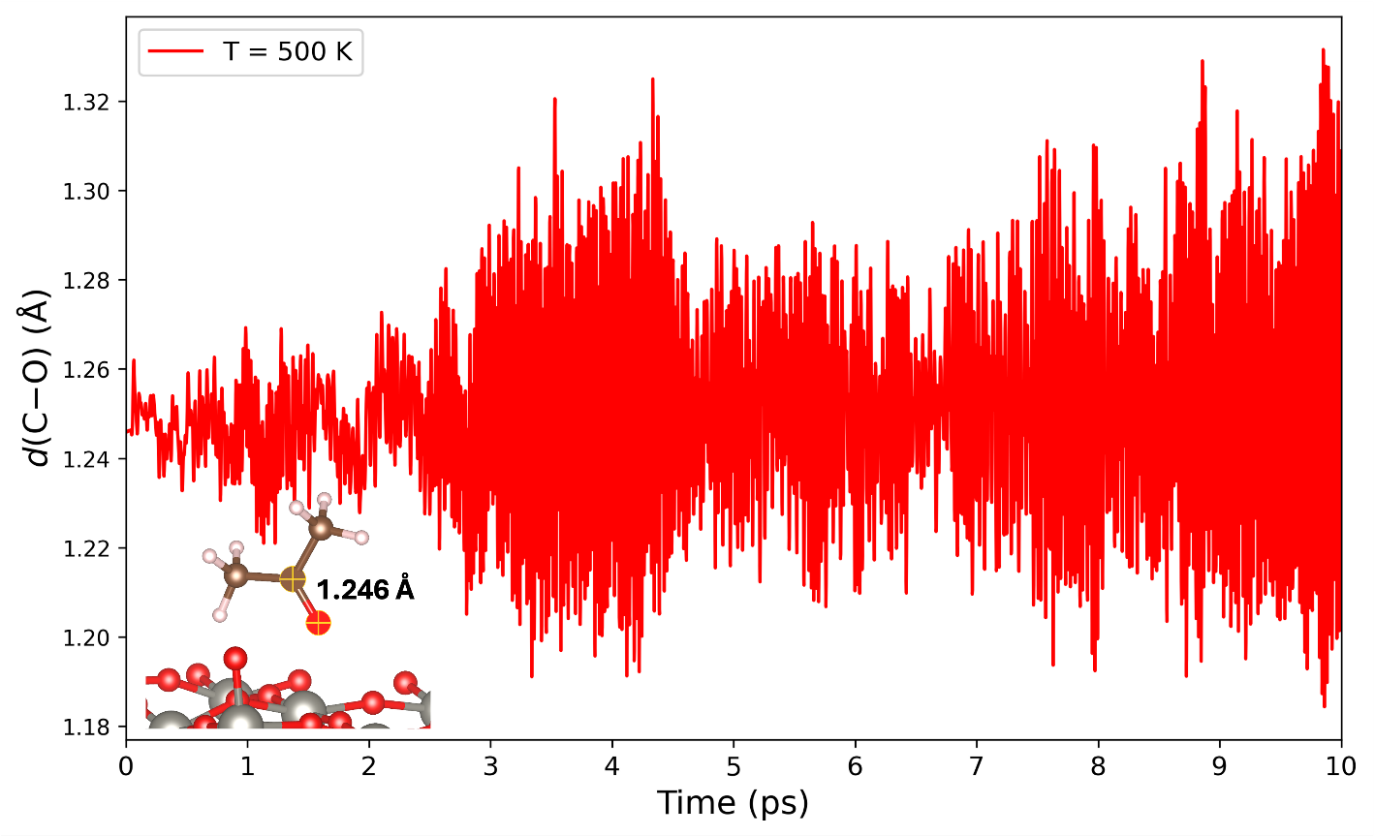


**Figure S10:** Time-trace of C=O bond length during AIMD of acetone adsorbed on *γ*-WO_3-x_ (001).


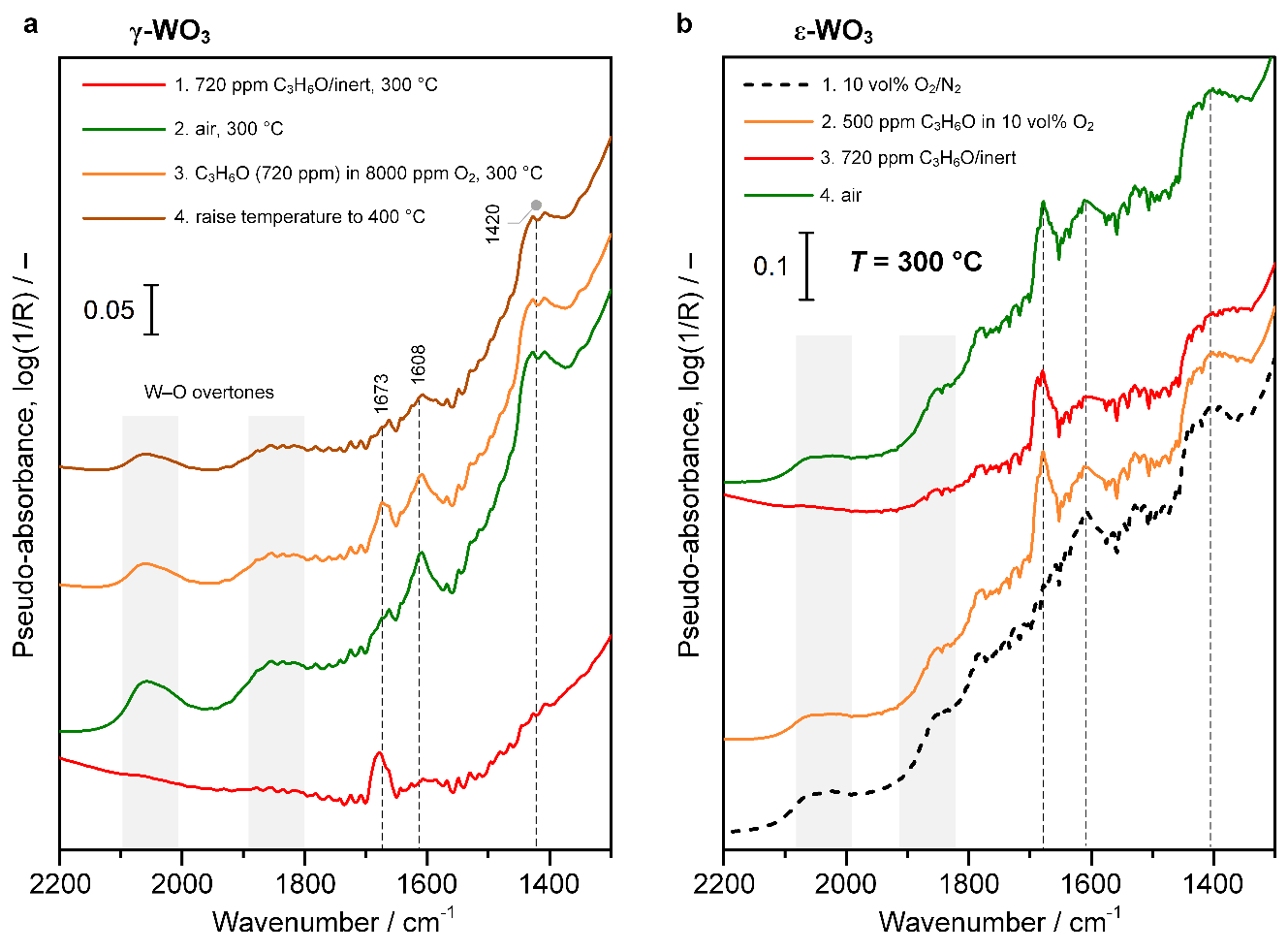


Figure S11: In situ DRIFT spectra of (a) γ- and (b) ε-WO_3_ under reactive exposure to lower acetone concentrations in different O_2_-containing (inert, 0.8 vol% and 10 vol%) backgrounds. Spectra are recorded sequentially according to the figure legends. Note the different sequences in (a) vs. (b).


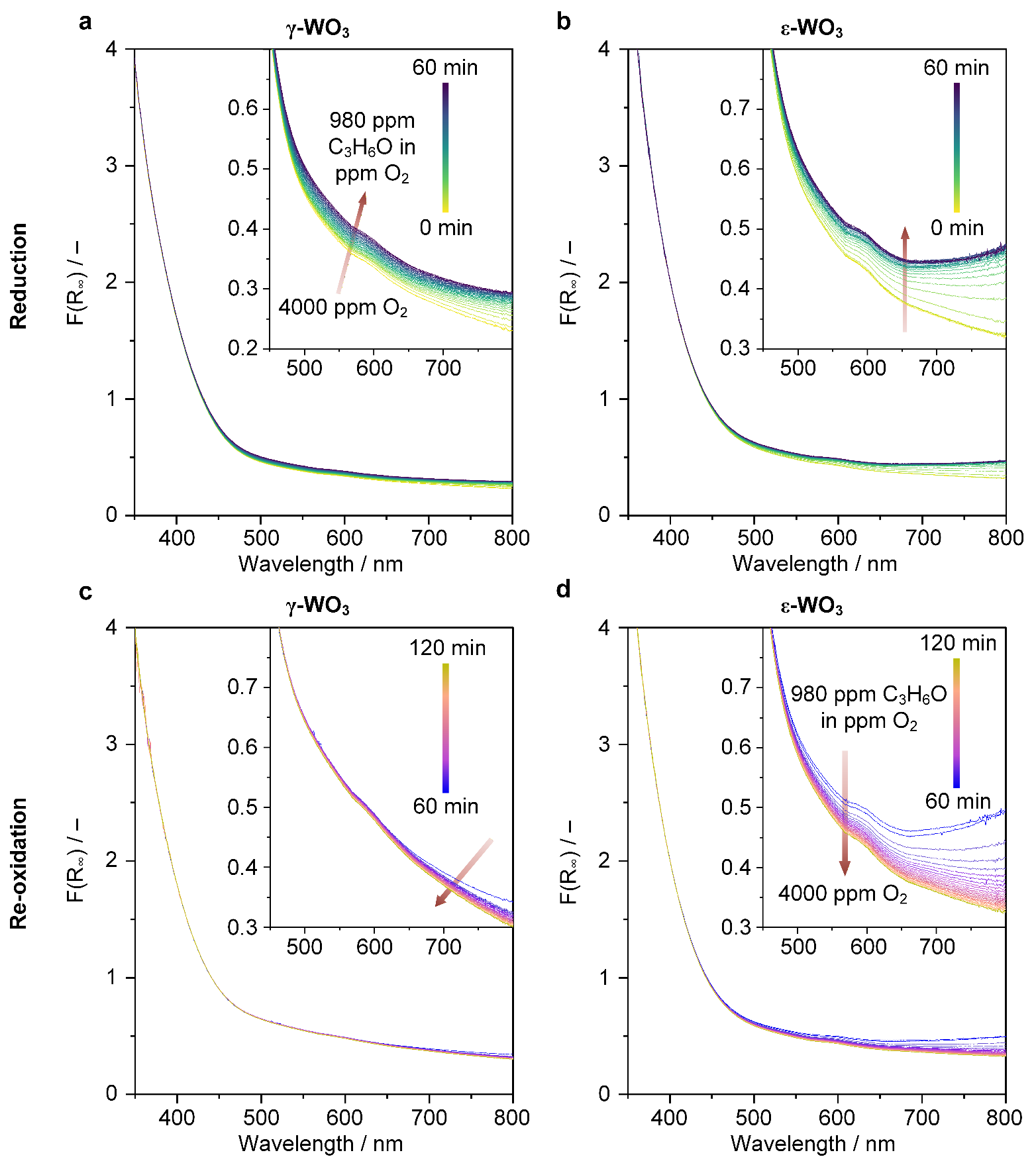


Figure S12: In situ UV-Vis DRS upon (a,d) admixing and (c,d) removing 980 ppm C_3_H_6_O to/from a background of 4000 ppm O_2_/N_2_.


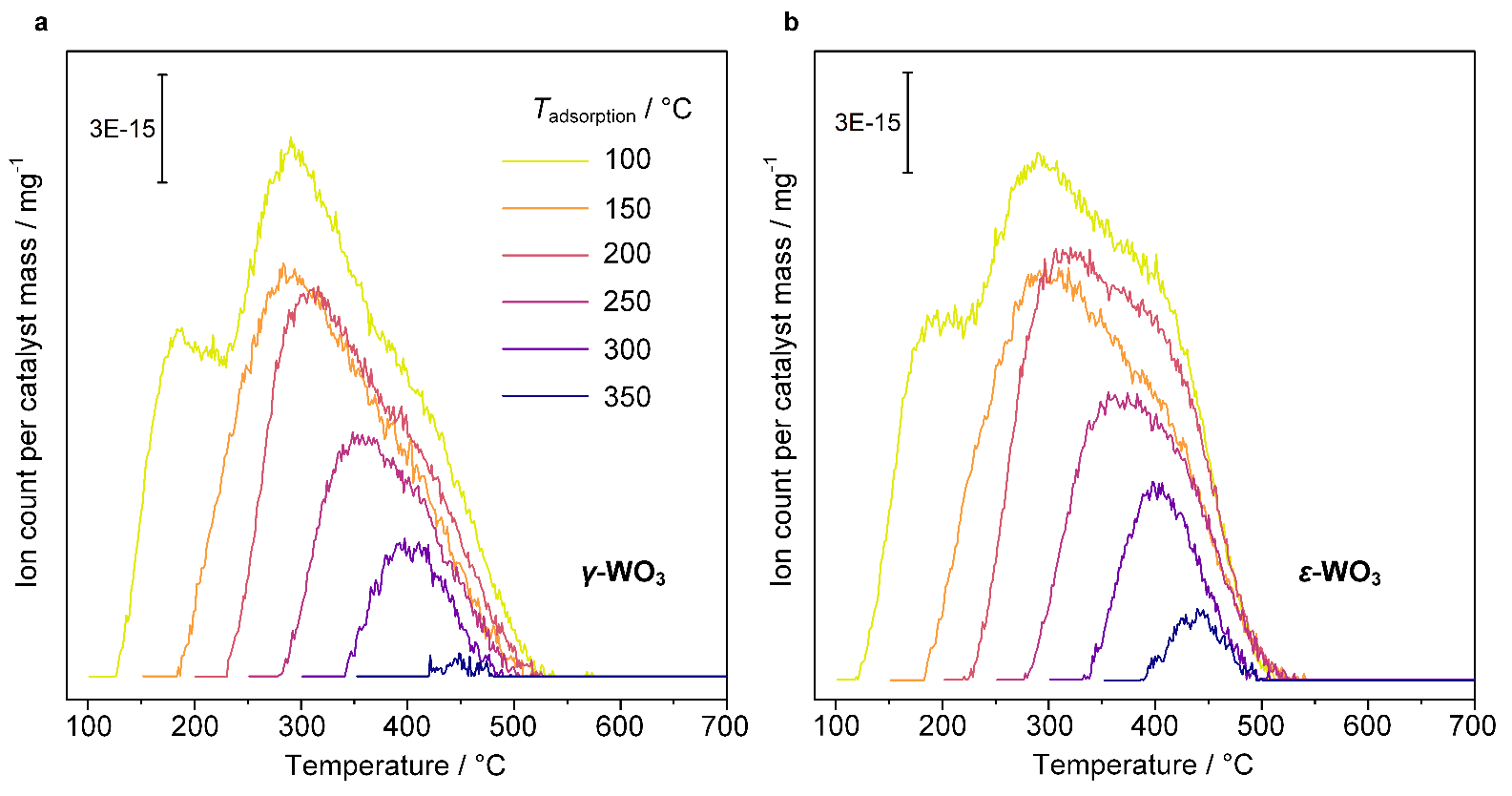


**Figure S13:** Pyridine (Py) evolution from (a) *γ*-WO_3_ and (b) *ε*-WO_3_ monitored at *m*/*z* = 79 during Py-TPD, following Py-adsorption at different temperatures between 100 – 350 °C.

**
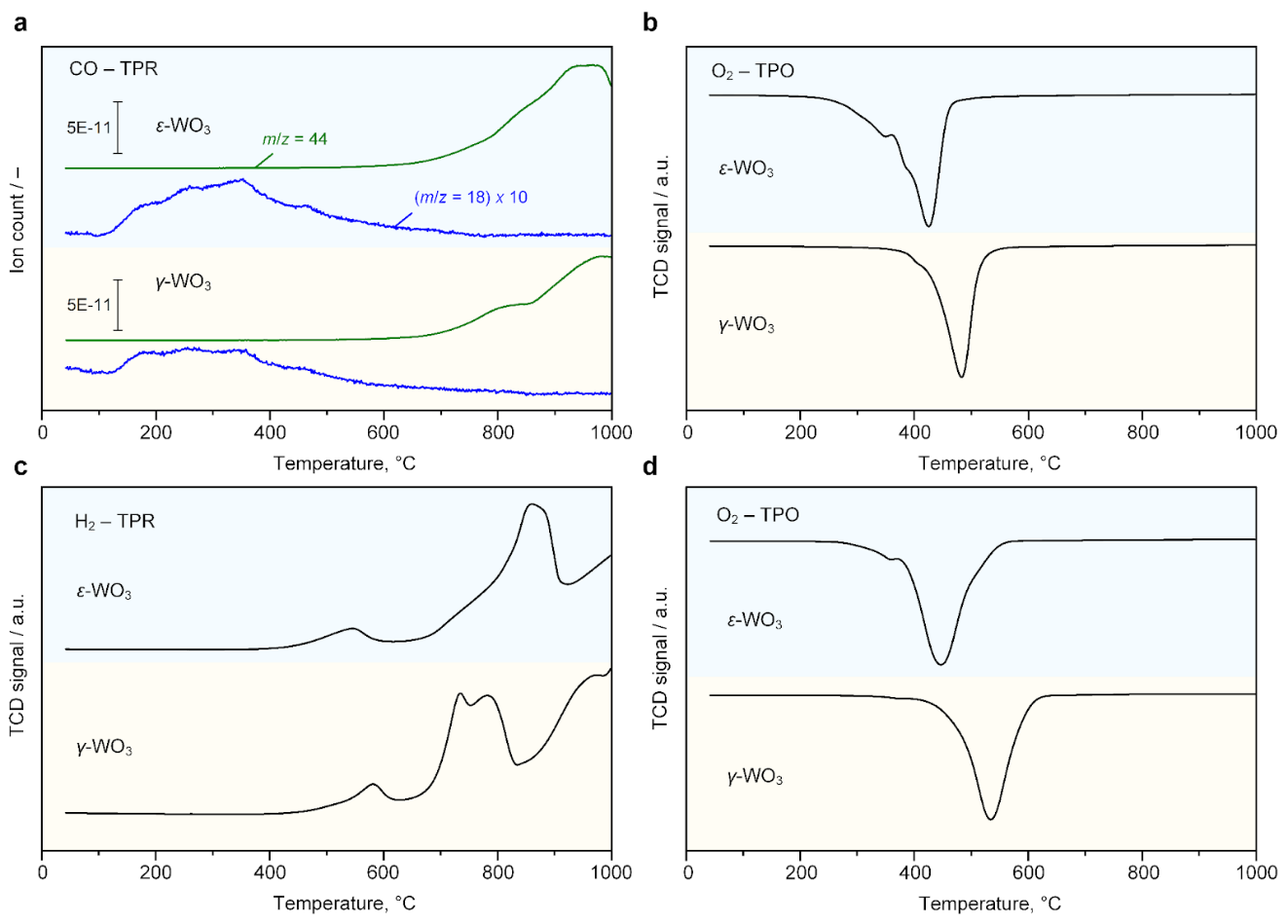
**

**Figure S14:** (a) CO-TPR followed by (b) O_2_-TPO as well as (c) H_2_-TPR followed by (d) O_2_-TPO. Note that, in (a), the *m*/*z* = 18 trace is scaled (*x*10) for both polymorphs.

**
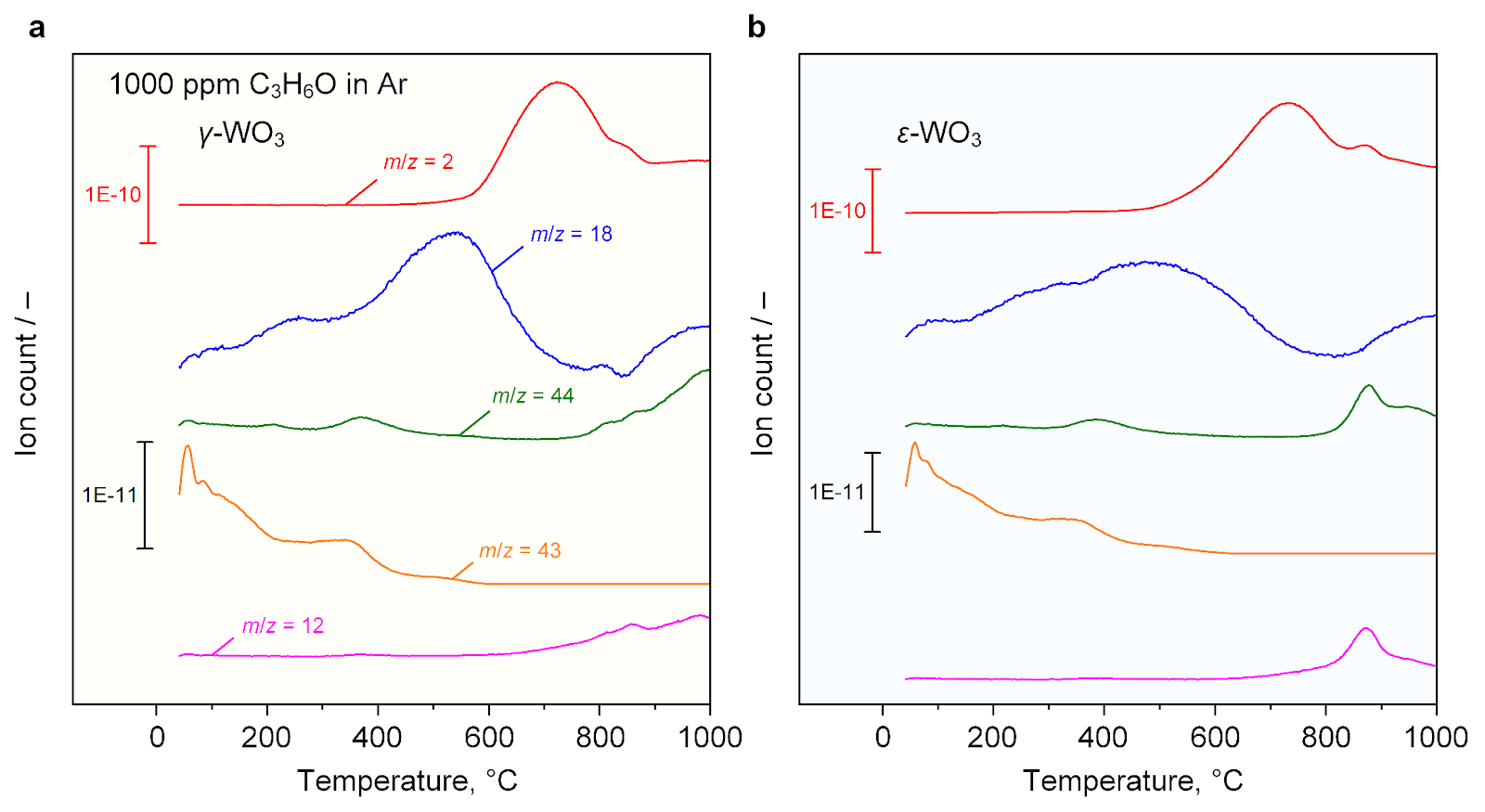
**

**Figure S15:** Evolution of *m*/*z* = 2, 18 and 44 during C_3_H_6_O-TPR performed over (a) *γ*-WO_3_ and (b) *ε*-WO_3_. Note that, in both (a) and (b), the *m*/*z* = 2 signal and *m*/*z* =12, 18, 43 and 44 are referenced to different scale bars of 1e-10 and 1e-11, respectively.

**
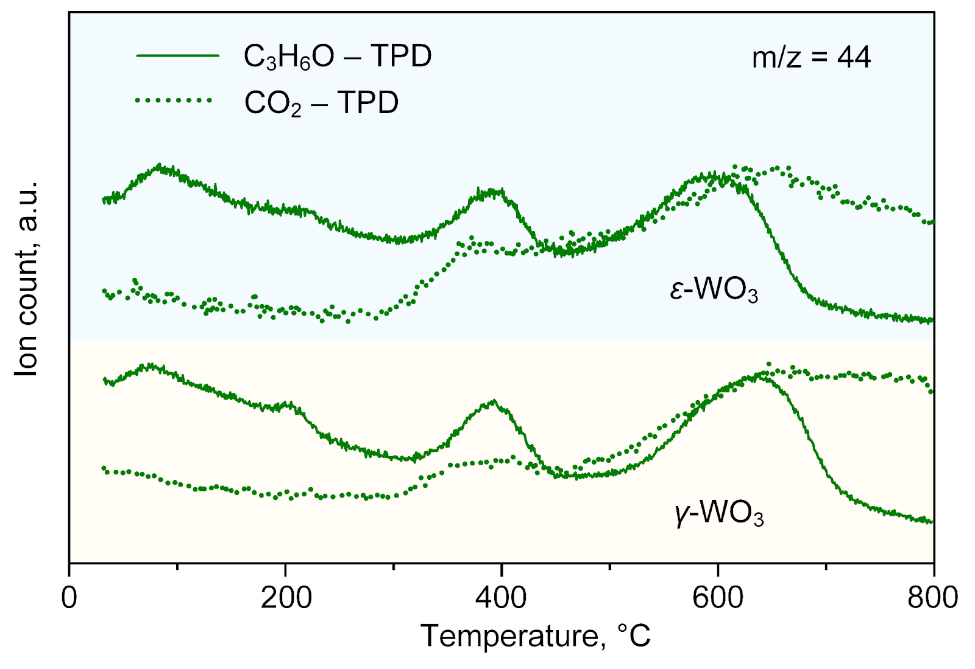
**

**Figure S16:** CO_2_-evolution (*m*/*z* = 44) during C_3_H_6_O-TPD and CO_2_-TPD over *γ*-WO_3_ and *ε*-WO_3_.


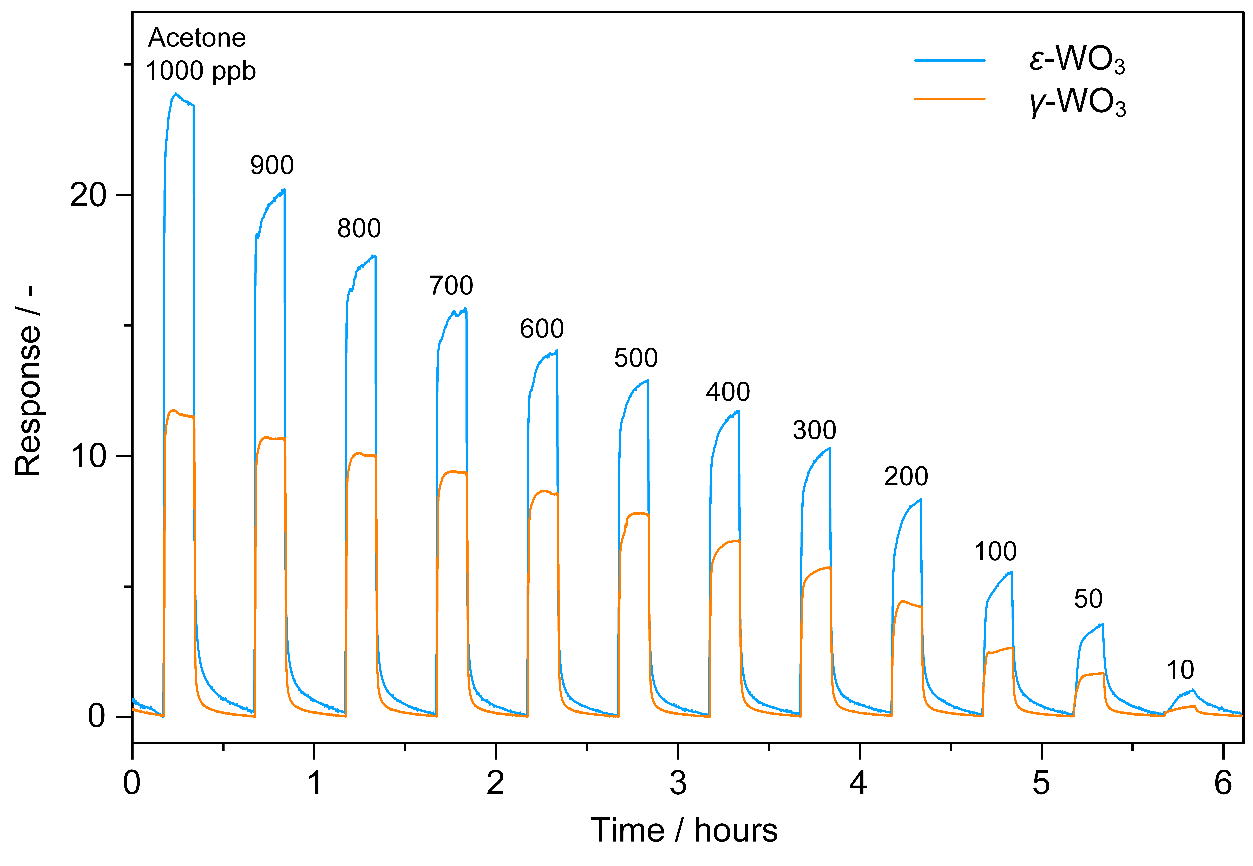


**Figure S17:** Sensor response transients of *γ*-WO_3_ and *ε*-WO_3_ upon exposure to 10 – 1000 ppb acetone at 330 °C in dry air.


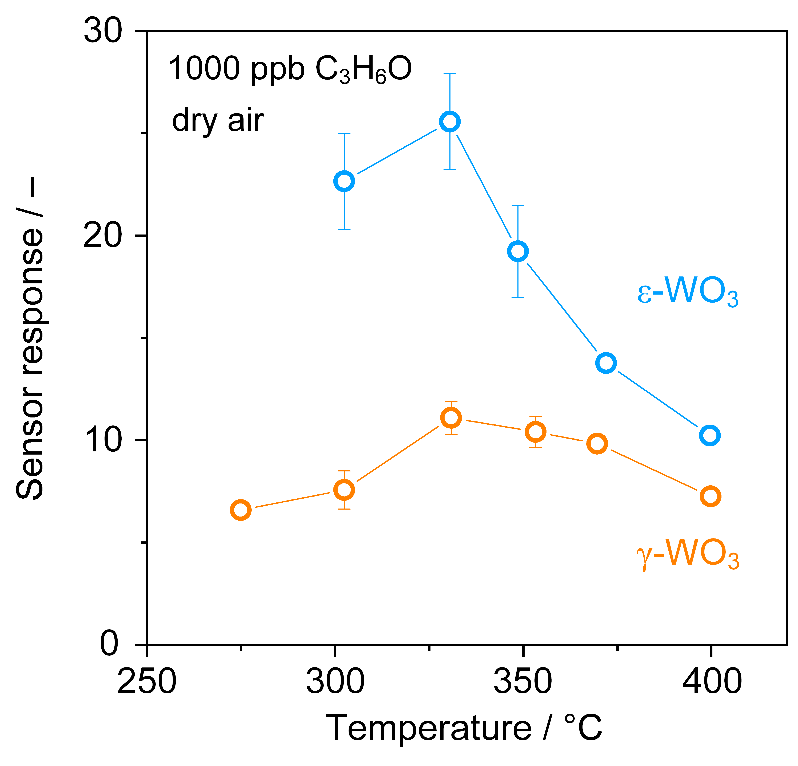


Figure S18: Sensor response of γ-WO_3_ and ε-WO_3_ towards 1000 ppb acetone in dry air as a function of operating temperature. Error bars represent the standard deviation of n = 3 identically prepared sensors.


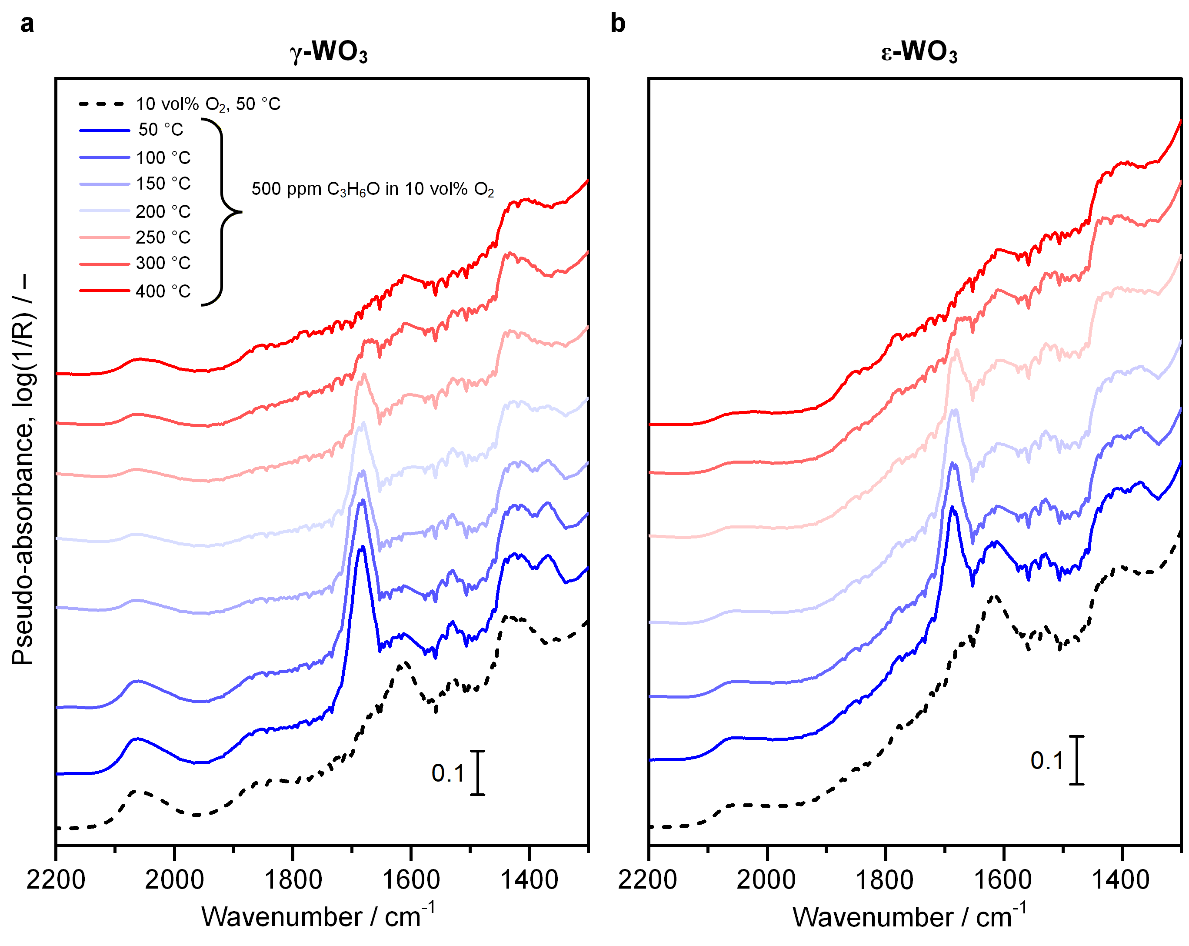


Figure S19: In situ DRIFT spectroscopy during reactive temperature ramps. (a) γ-WO_3_ and (b) ε-WO_3_ with acetone (500 ppm) in 10 vol% O_2_ background.


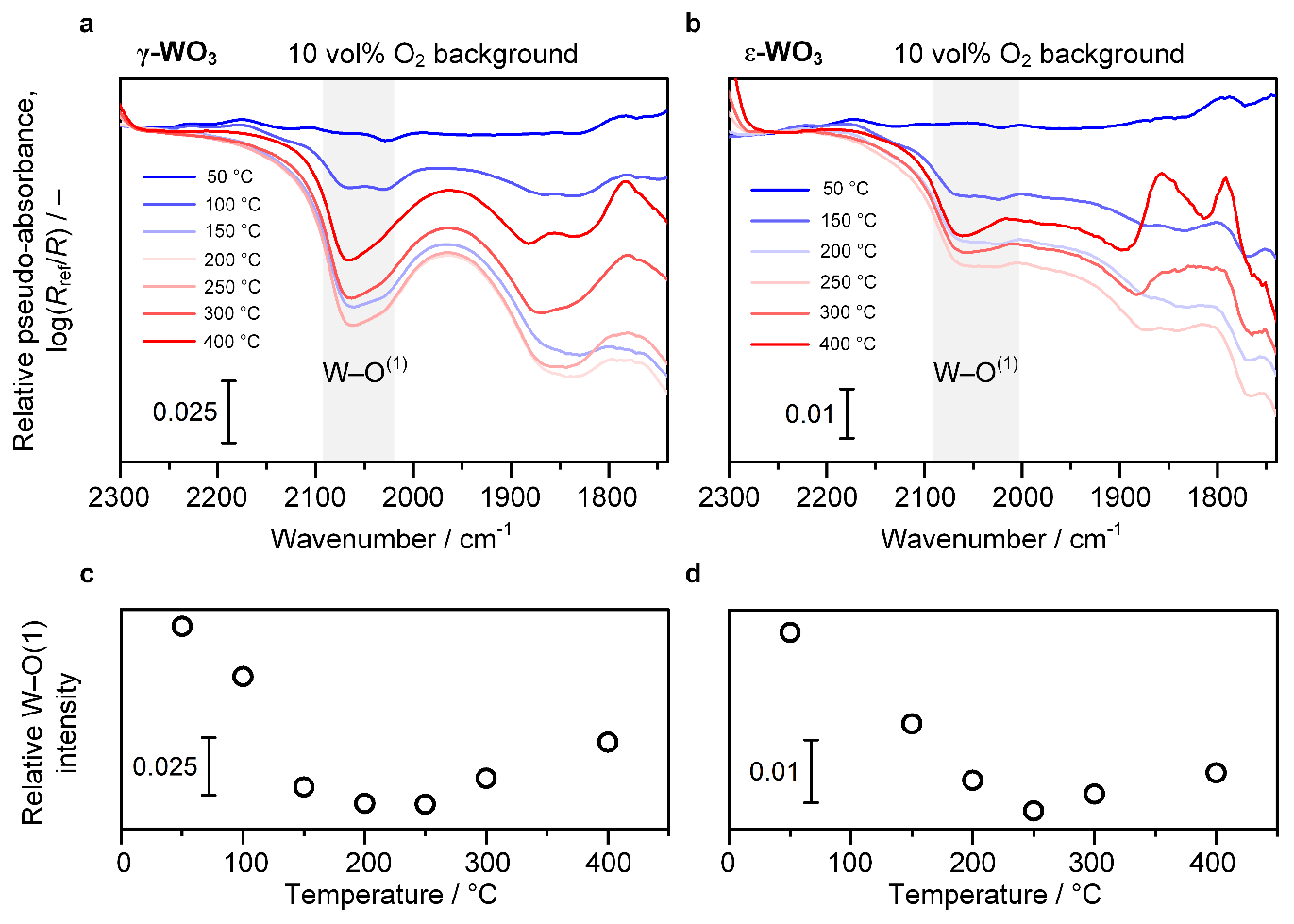


Figure S20: (a,b) Relative pseudo-absorbance derived from Figure R6, referenced to the spectrum before admixing acetone, along (c,d) with average (relative) pseudo-intensity of W–O^(1)^ lattice overtone.


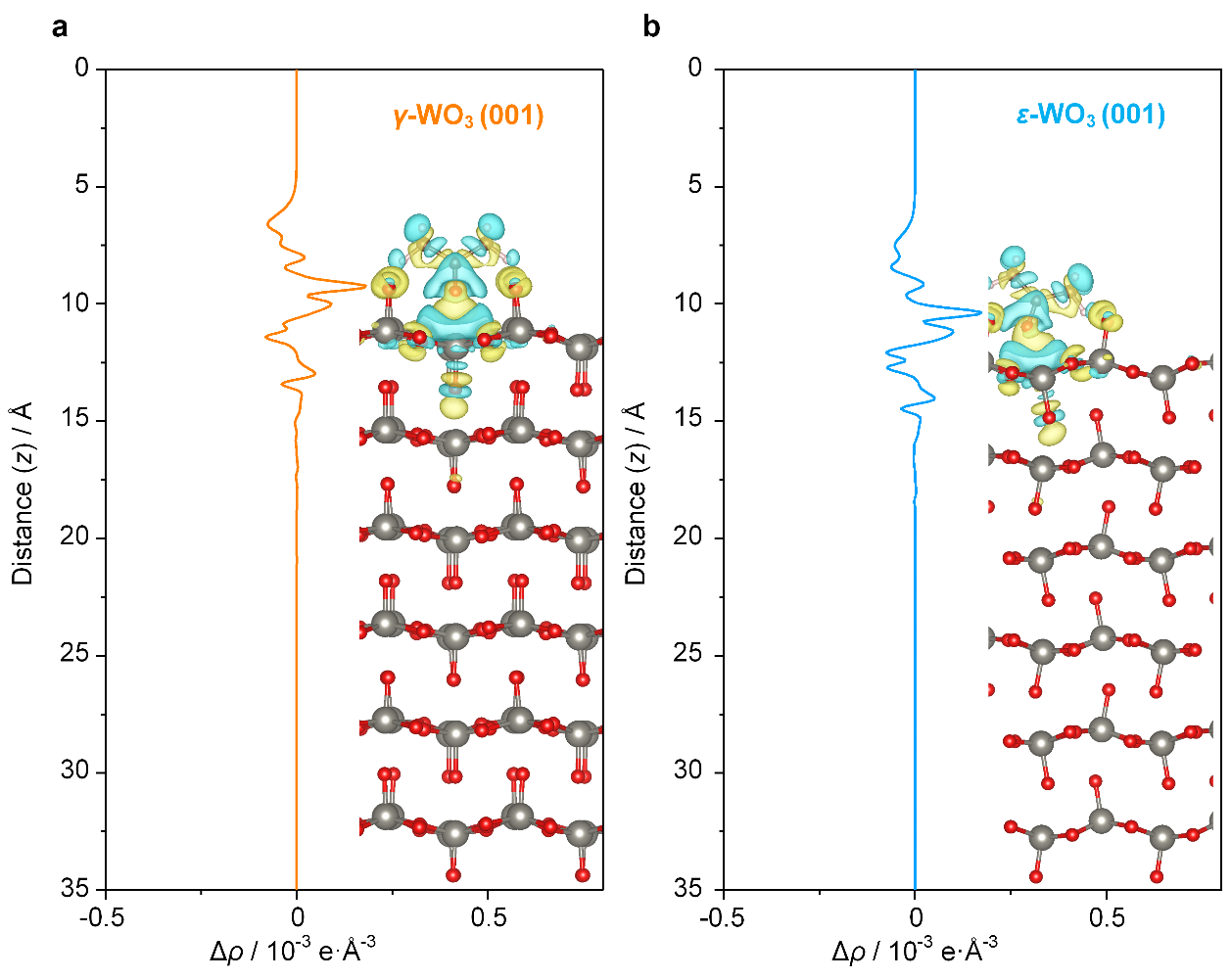


**Figure S21:** Δ*ρ*(*z*) along with CDD map of acetone adsorbed onto stoichiometric (a) *γ*- and (b) *ε*-WO_3_.


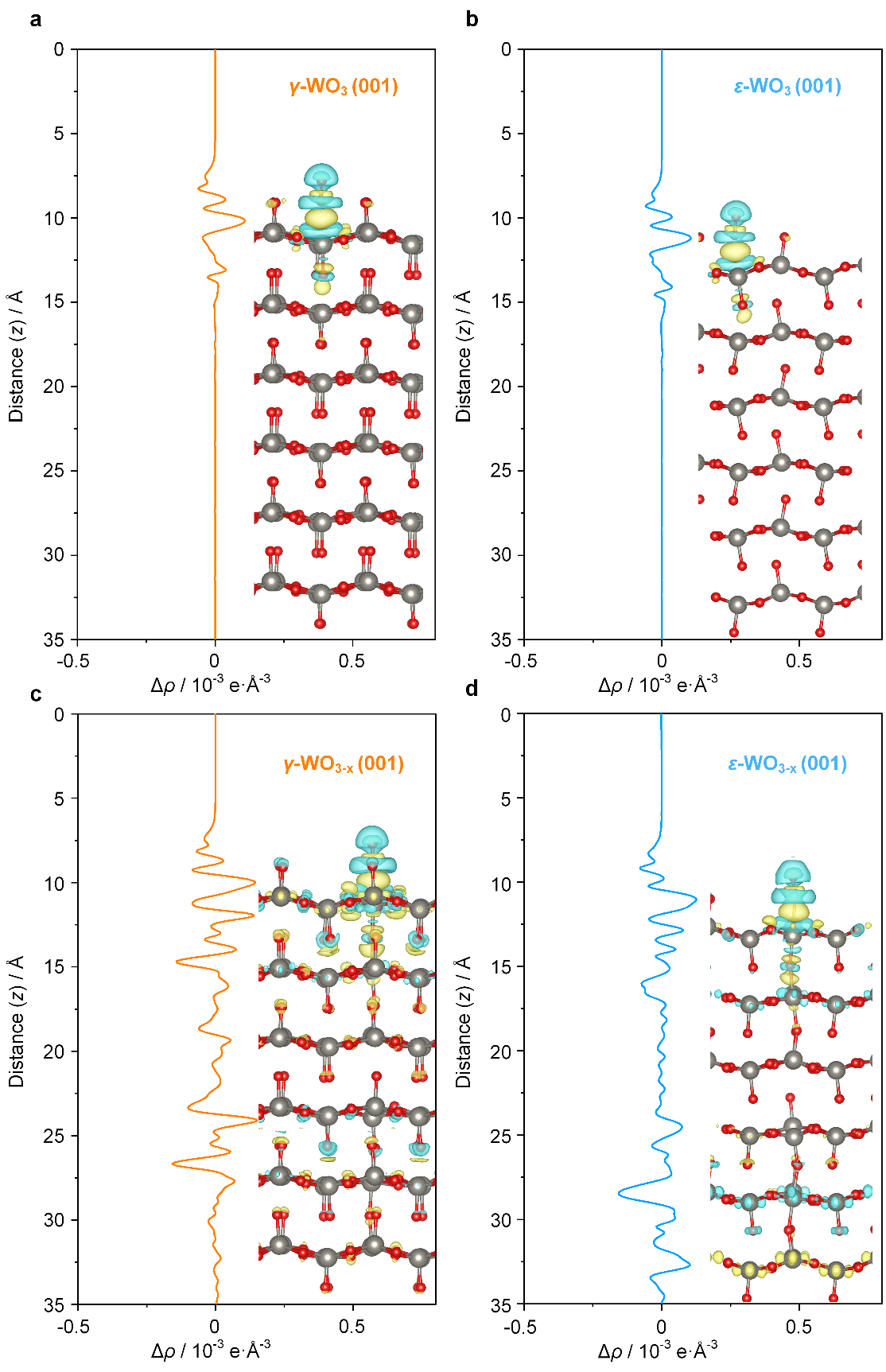


**Figure S22:** CDD maps of CO adsorbed on stoichiometric (a) *γ*– and (b) *ε*–WO_3_, as well as reduced (c) *γ*– and (d) *ε*–WO_3-x_, along with their respective plane-averaged Δ*ρ*(*z*).


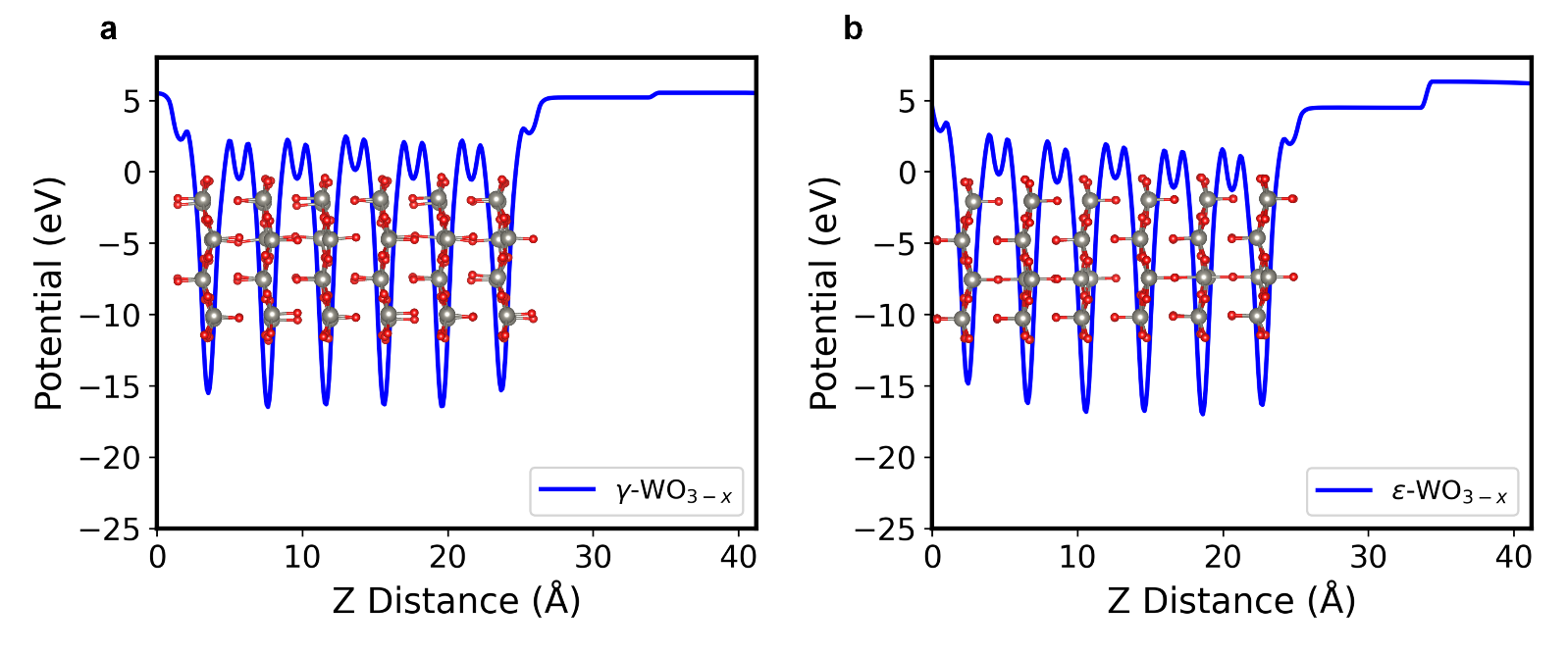


**Figure S23:** Electrostatic potential profiles across (a) *γ*- and (b) *ε*-WO_3-x_ (001) slabs.

**
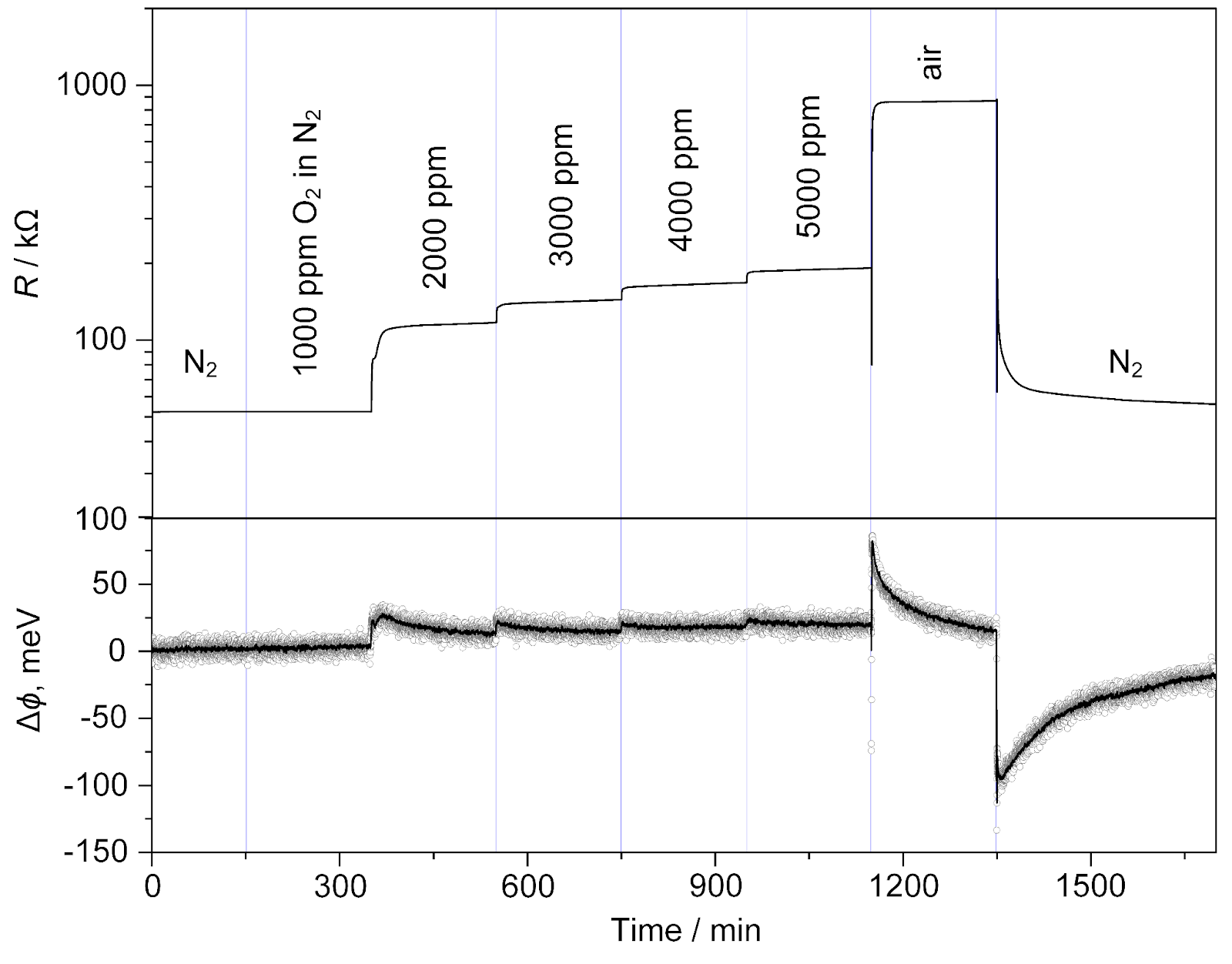
**

**Figure S24:** *Operando* work function measurements upon exposing *γ*-WO_3_ to 0 – 20 vol% O_2_ in N_2_ at 330 °C.

**
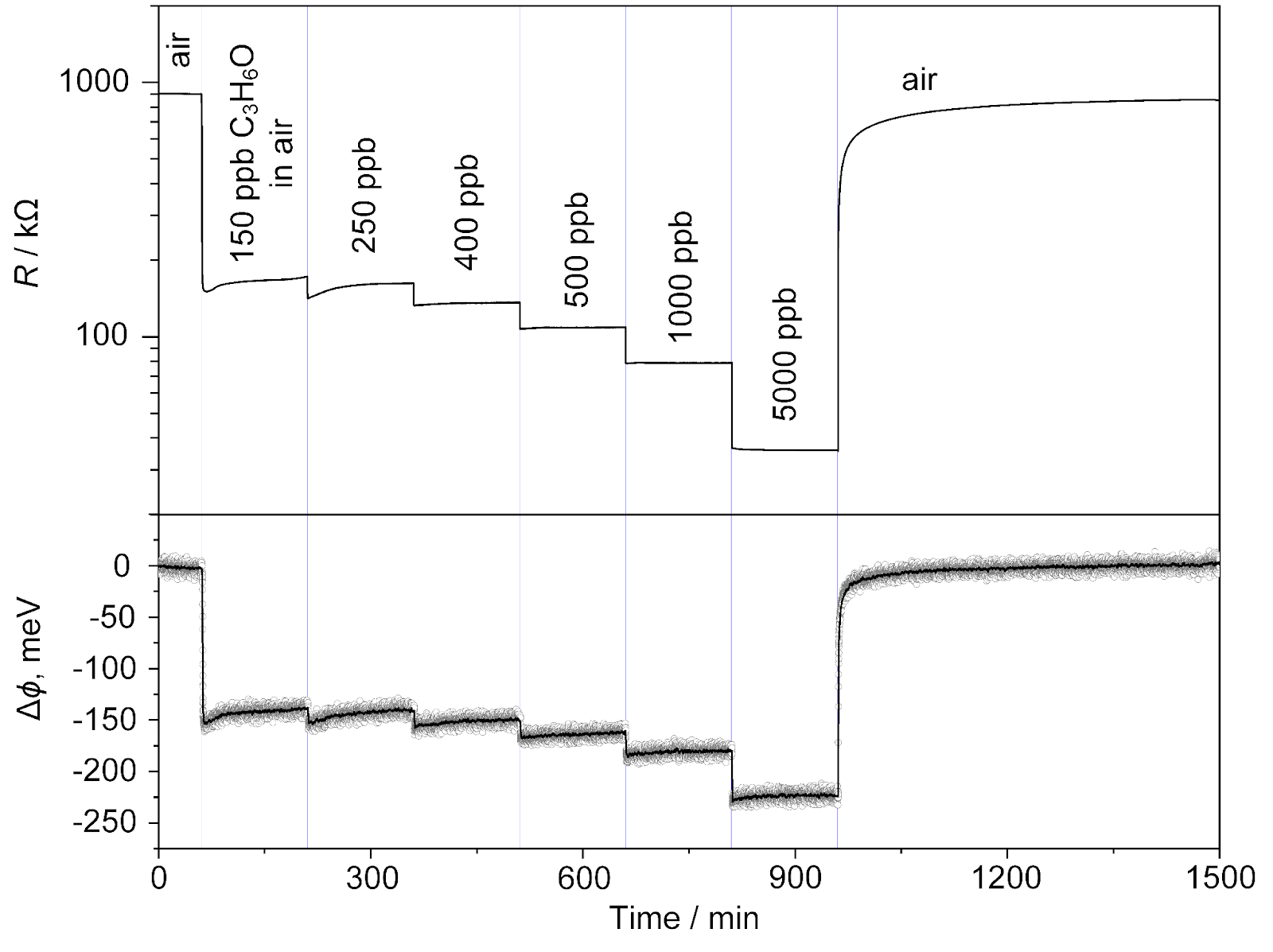
**

**Figure S25:** *Operando* work function measurements upon exposing *γ*-WO_3_ to 150 – 5000 ppb C_3_H_6_O in air at 330 °C.

**
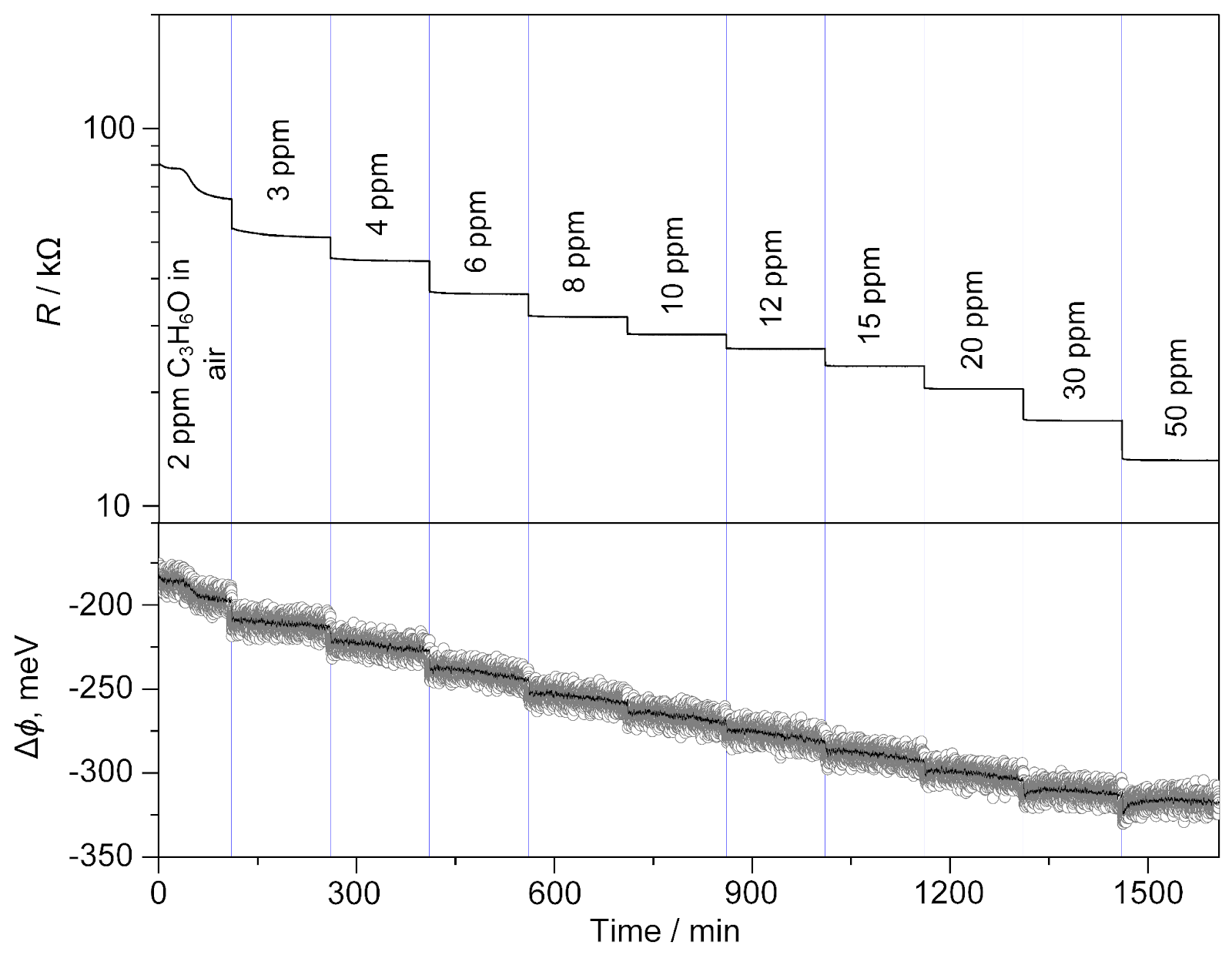
**

**Figure S26:** *Operando* work function measurements upon exposing *γ*-WO_3_ to 2 – 50 ppm C_3_H_6_O in air at 330 °C.

**
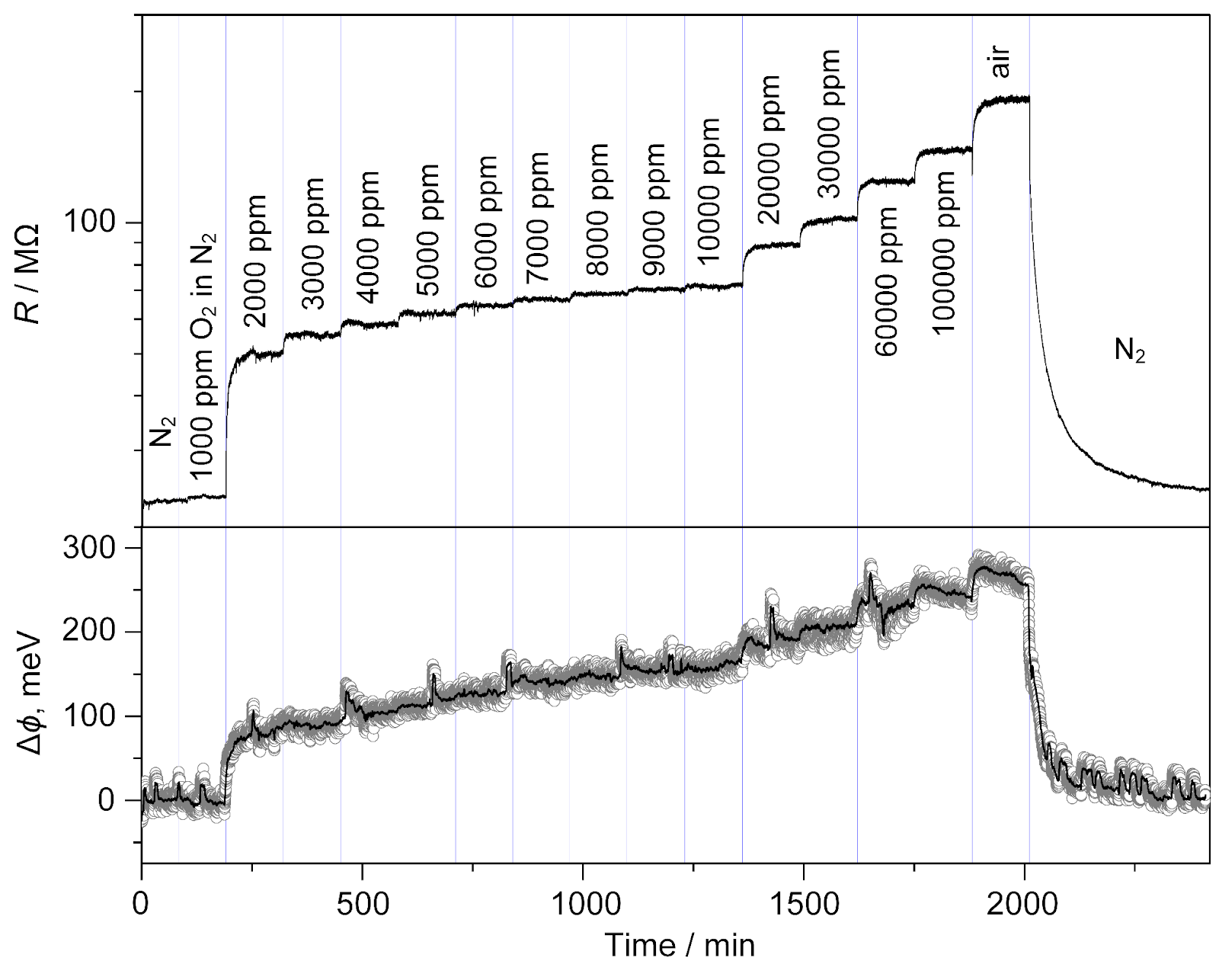
**

**Figure S27:** *Operando* work function measurements upon exposing *ε*-WO_3_ to 0 – 20 vol% O_2_ in N_2_ at 330 °C.

**
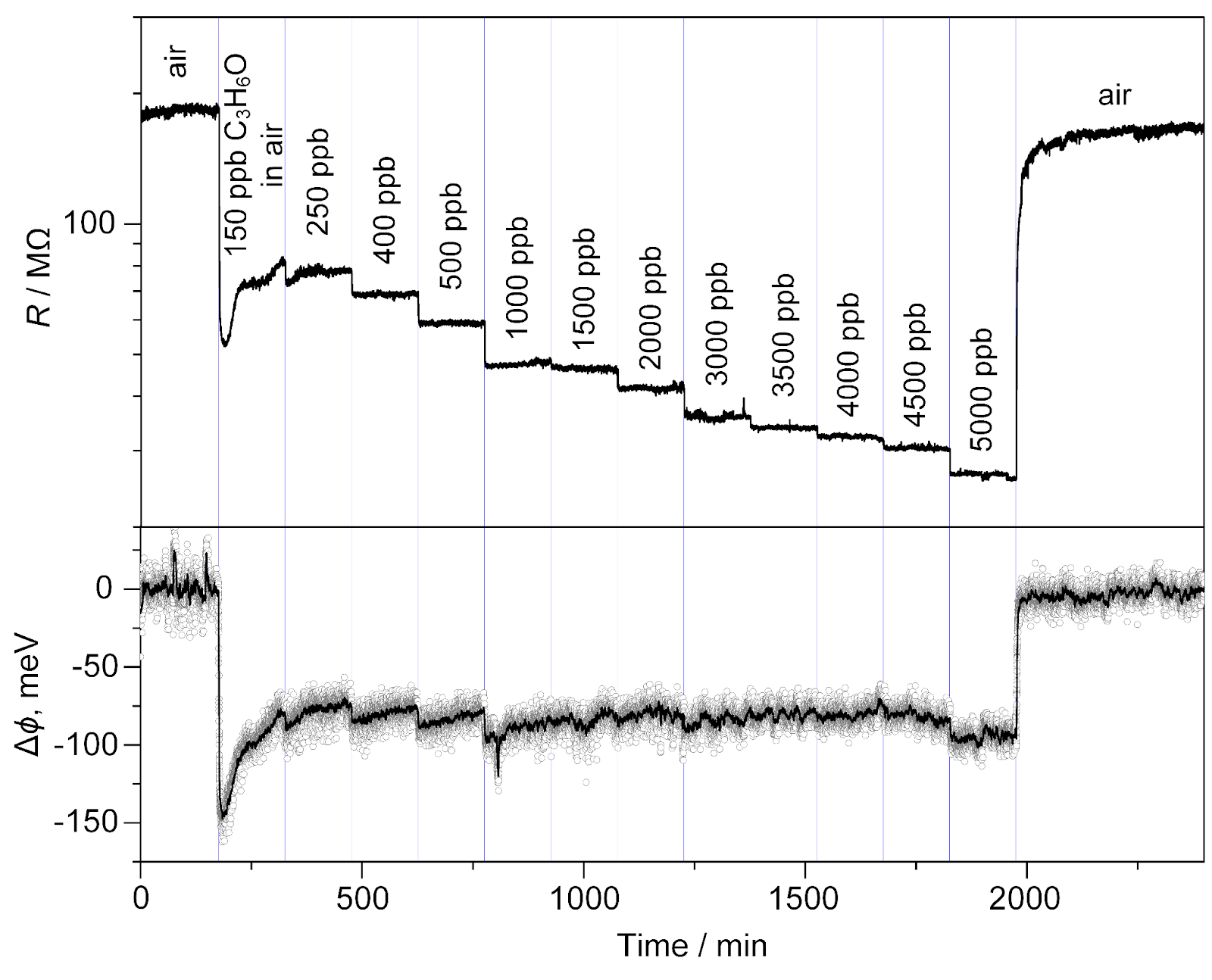
**

**Figure S28:** *Operando* work function measurements upon exposing *ε*-WO_3_ to 150 – 5000 ppb C_3_H_6_O in air at 330 °C.

**
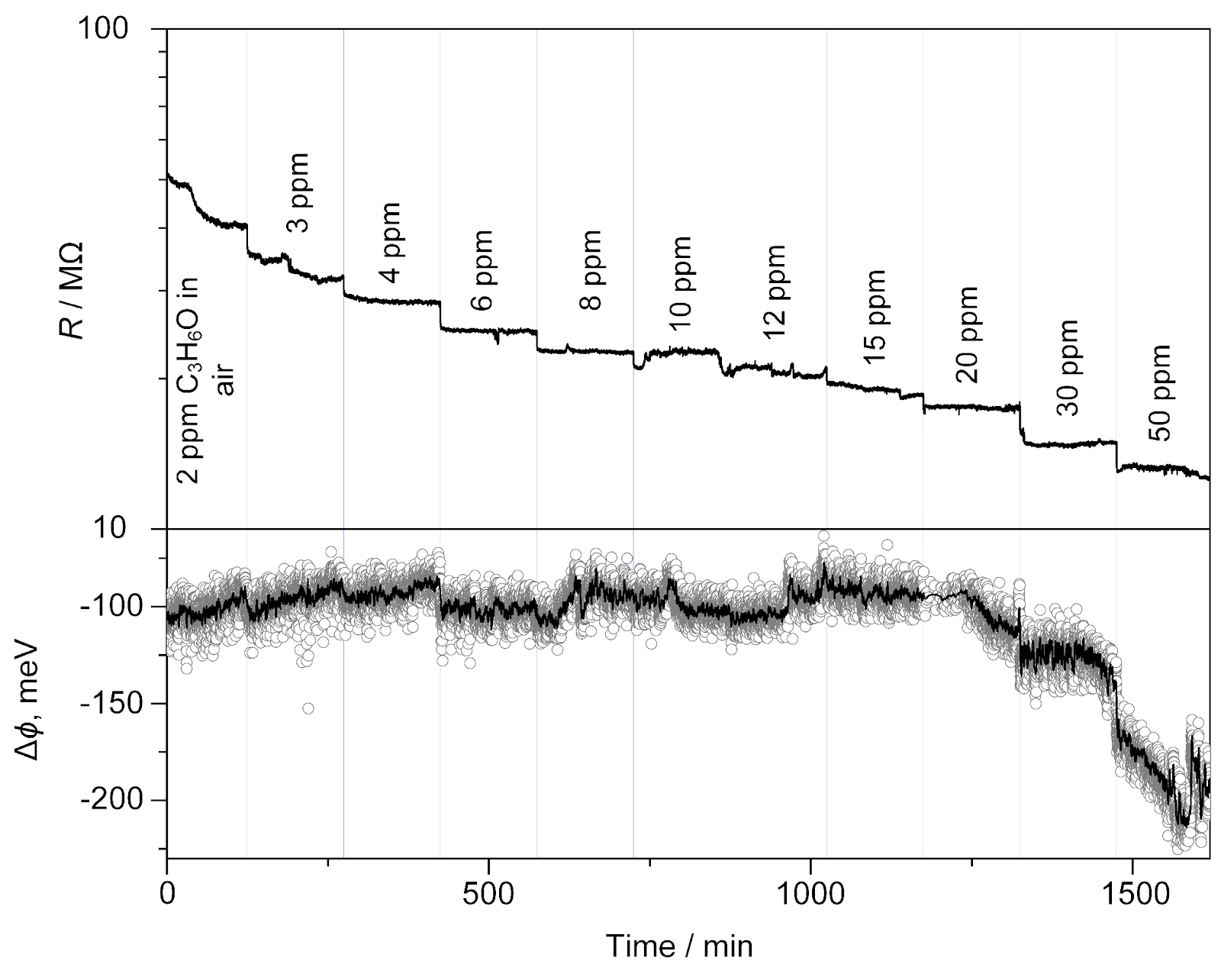
**

**Figure S29:** *Operando* work function measurements upon exposing *ε*-WO_3_ to 2 – 50 ppm C_3_H_6_O in air at 330 °C.


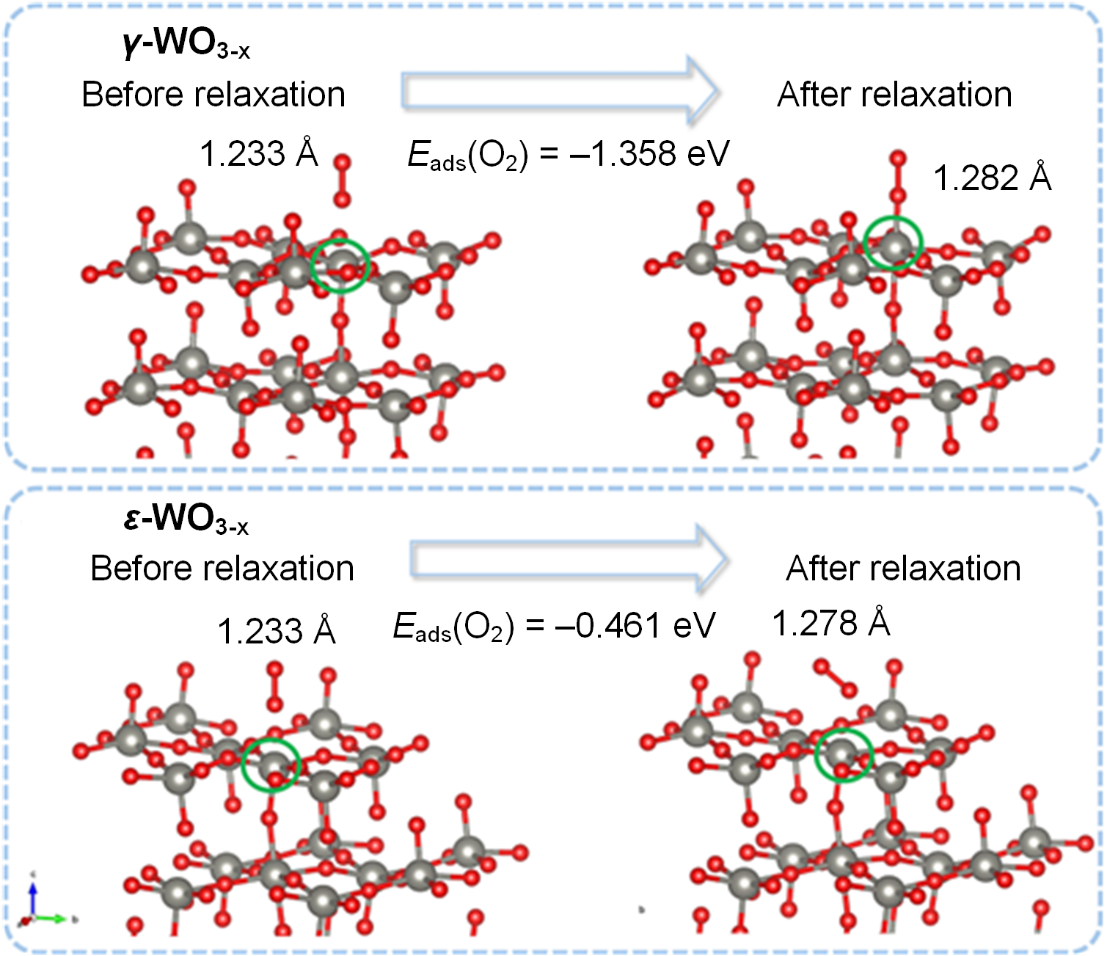


**Figure S30:** Oxygen molecule adsorbed on *γ*-WO_3-x_ (001) and *ε*-WO_3-x_ (001) at the oxygen vacancy site. Indicated are the values of *E*_ads_ as well as O–O bond length before and after adsorption.

**
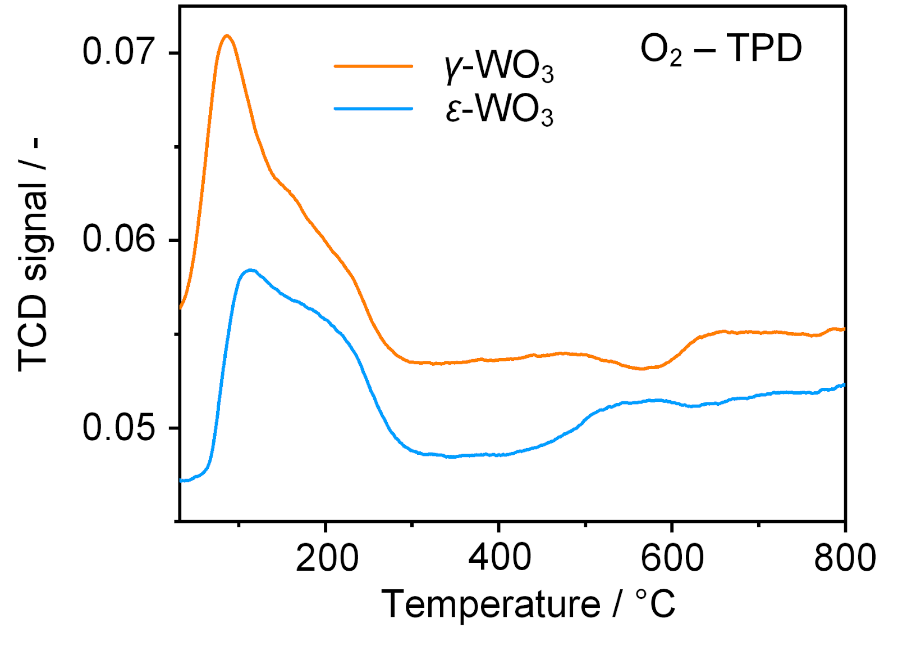
**

**Figure S31:** TCD signal during O_2_-desorption from *γ*-WO_3_ and *ε*-WO_3_.


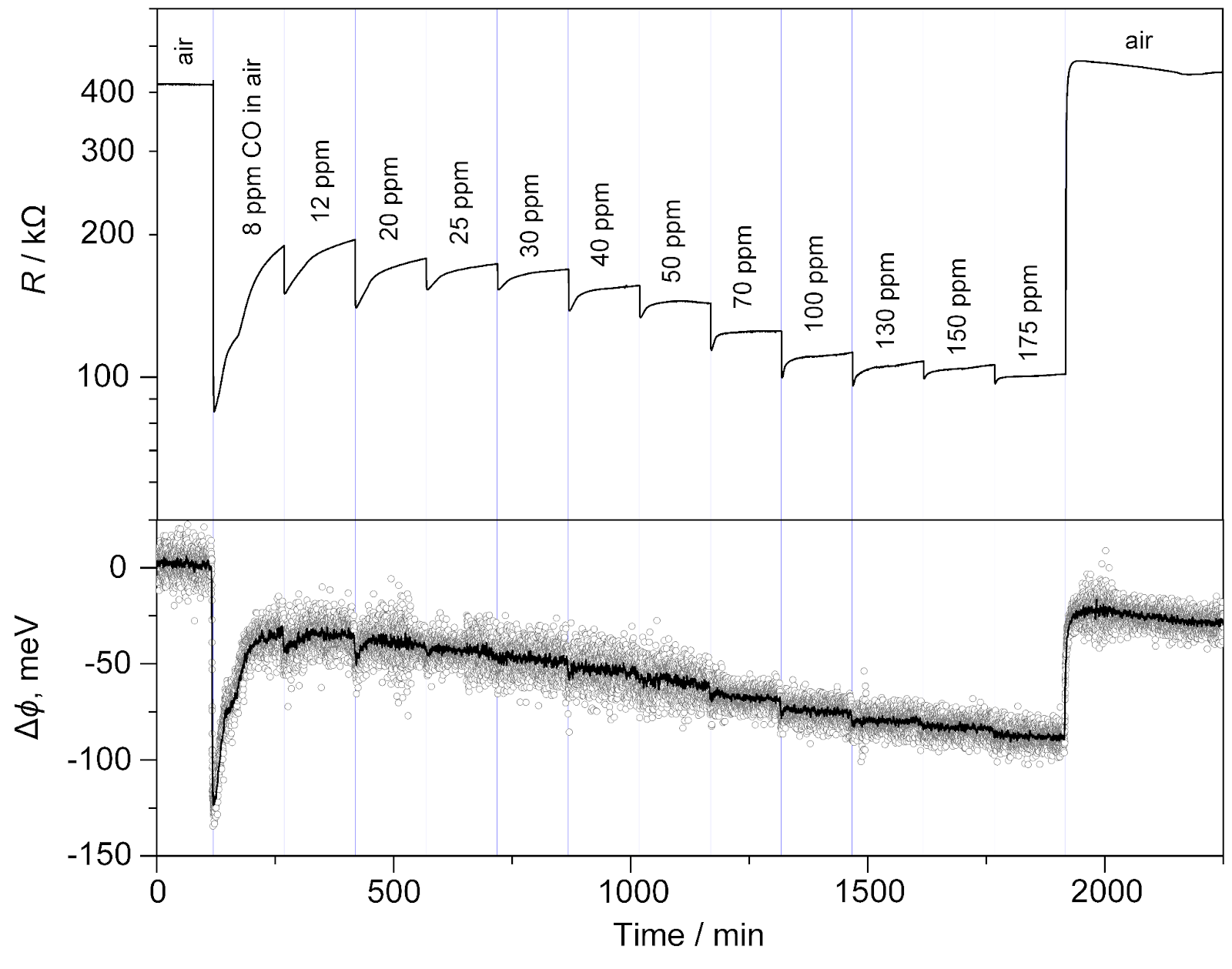


**Figure S32:** *Operando* work function measurements during exposure of SnO_2_ to 8 – 175 ppm CO in air at 400 °C.


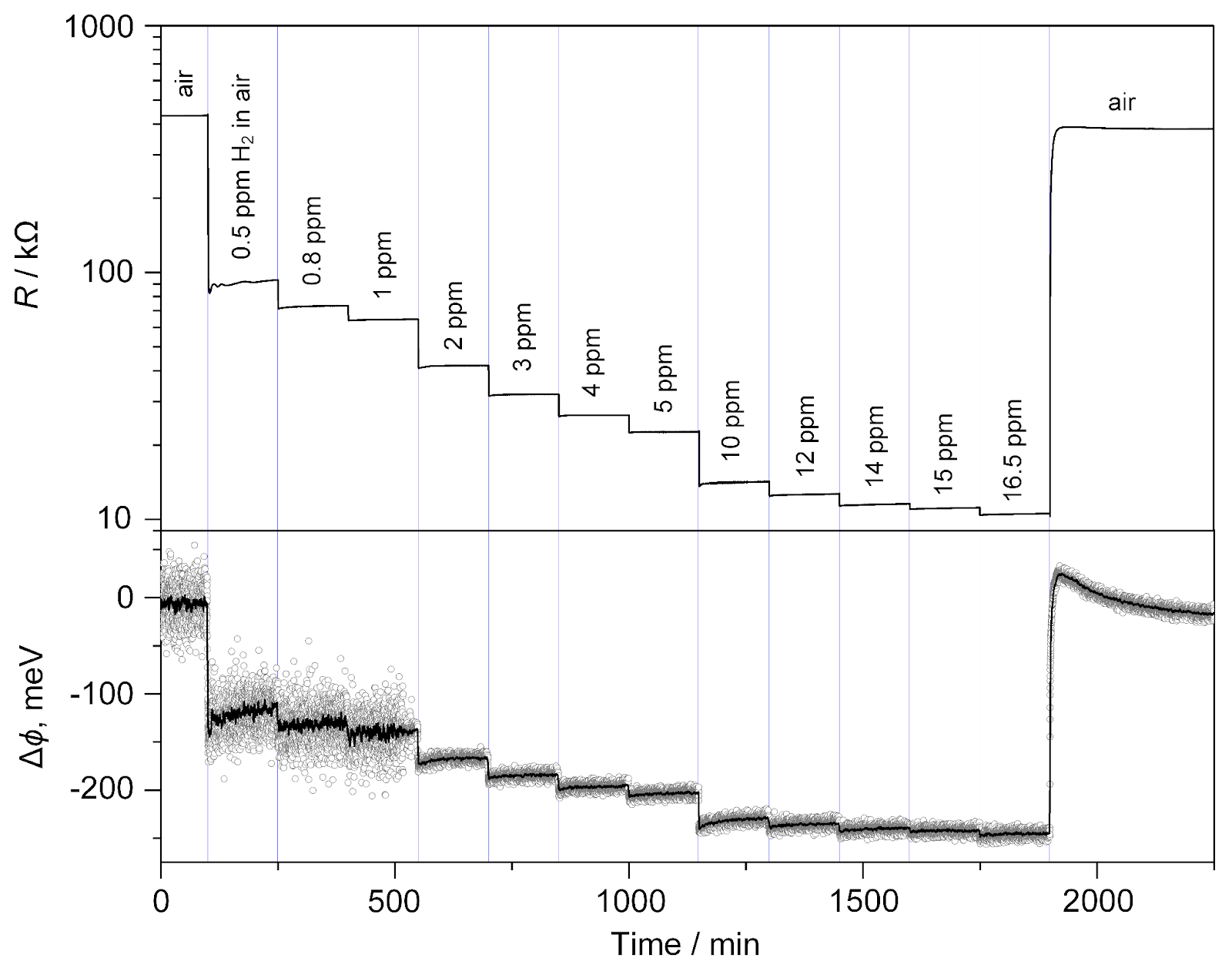


**Figure S33:** *Operando* work function measurements during exposure of SnO_2_ to 0.5 – 16.5 ppm H_2_ in air at 400 °C.


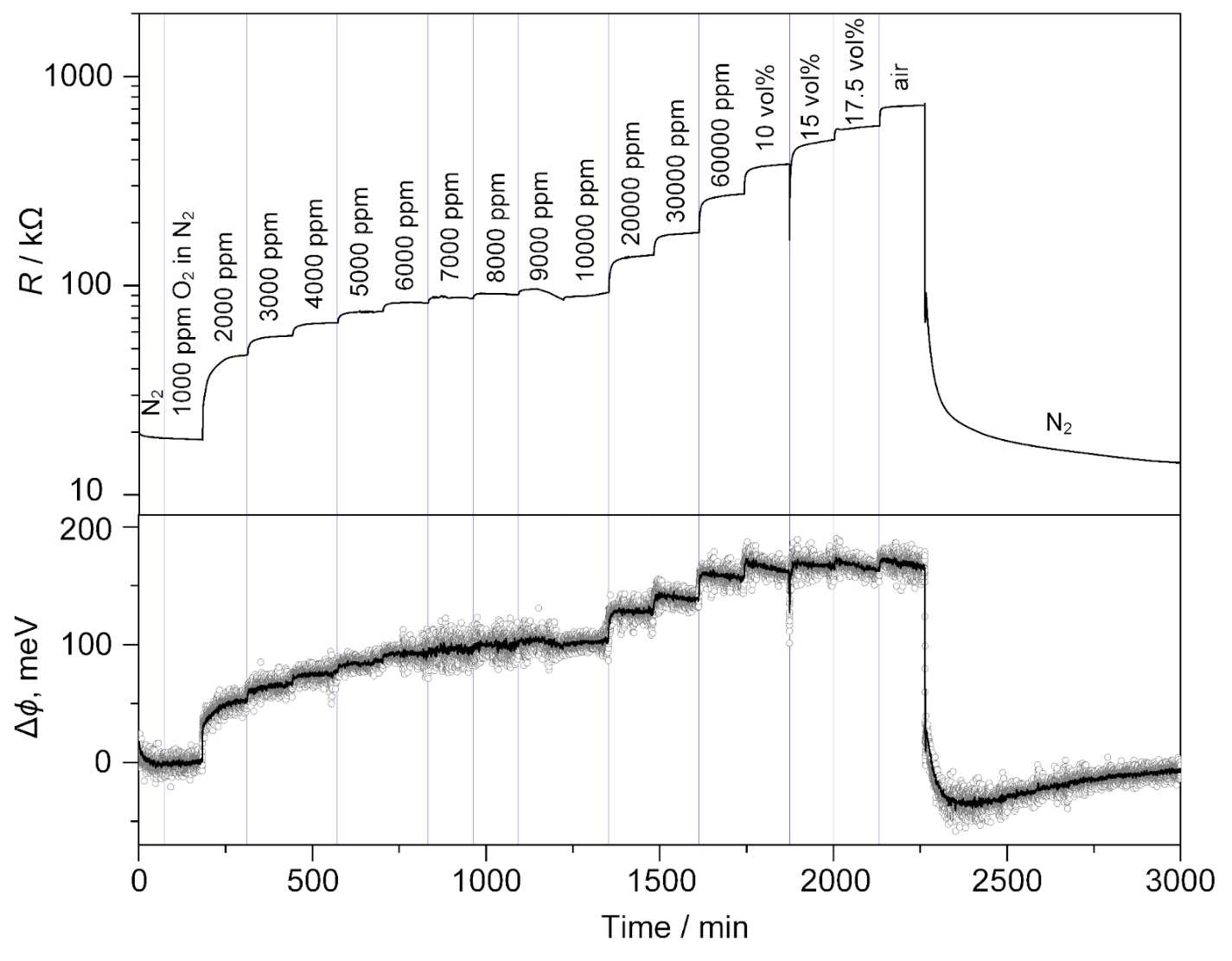


**Figure S34:** *Operando* work function measurements during exposure of SnO_2_ to 0.1 – 20 vol% O_2_ in N_2_ at 300 °C.


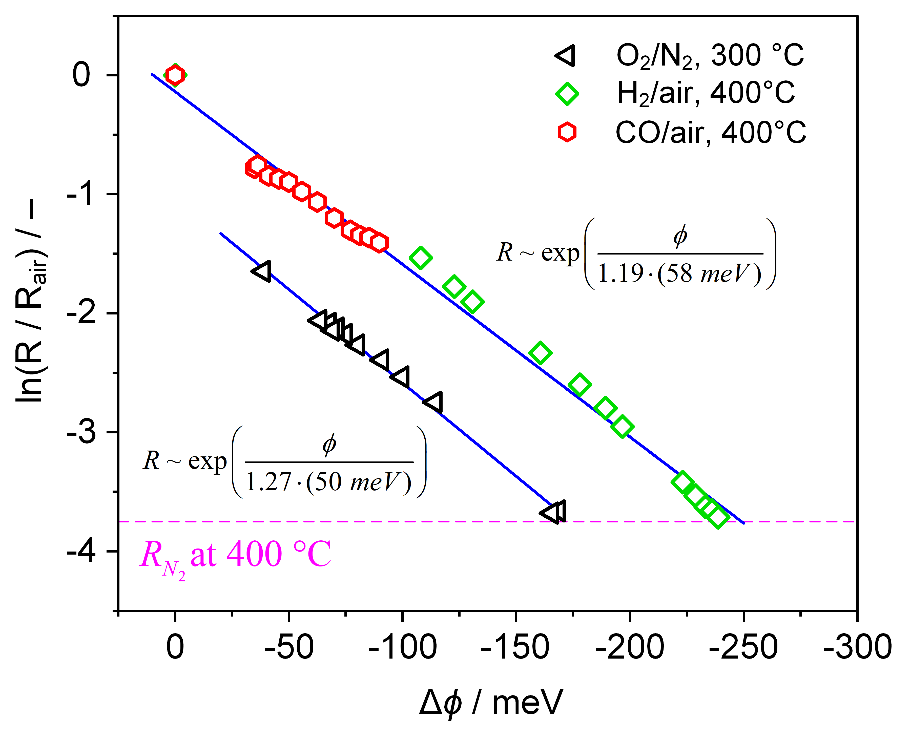


**Figure S35:** *Operando* work function results of SnO_2_. Note that O_2_/N_2_ data are collected at lower temperature than O_2_/air and H_2_/air.


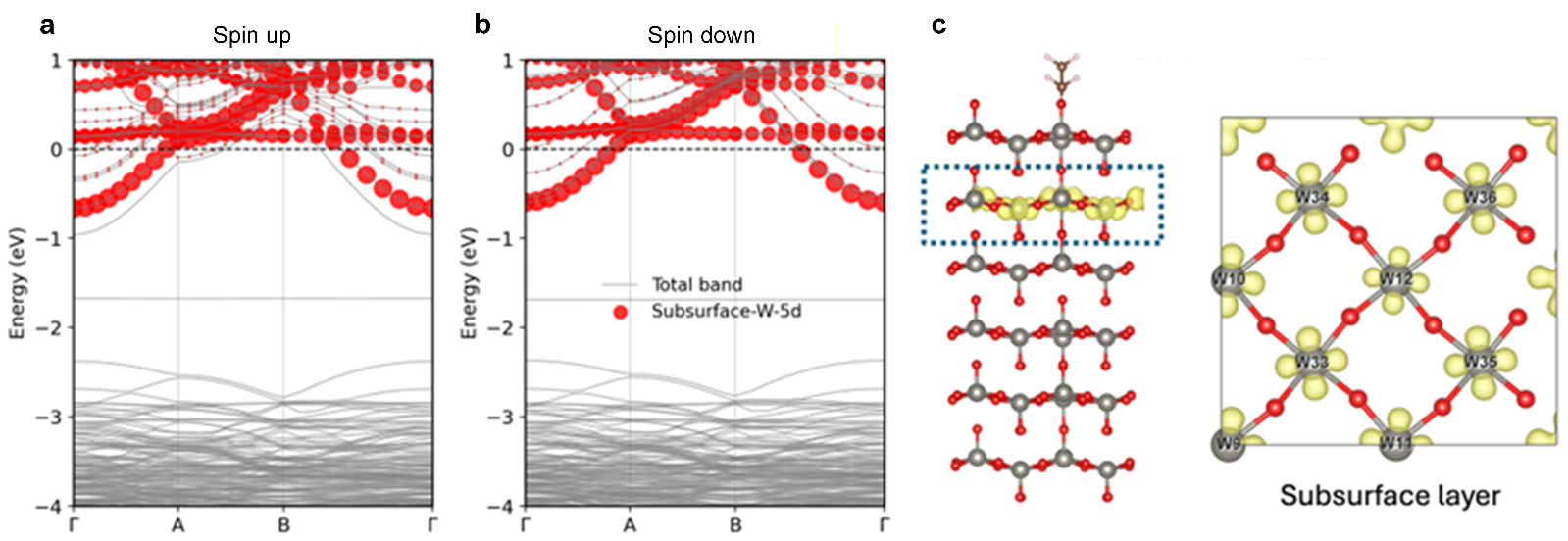


Figure S36: Total band structures (grey) and band structures projected onto the W-5d orbitals of subsurface W atoms (red dots) for acetone adsorbed on oxygen-deficient ε-WO_3-x_: (a) spin-up, (b) spin-down, and (c) spin-density ($\boldsymbol{\rho}_{\boldsymbol{\uparrow}}\boldsymbol{-}\boldsymbol{\rho}_{\boldsymbol{\downarrow}}$) distribution together with the extracted subsurface layers. Oxygen and tungsten are indicated as red and gray spheres, respectively.

**Table S1:** XRD refinement results.

|  | **Monoclinic *γ*-WO_3_ (P2_1_/n, #14)** | | | | | | | **Monoclinic *ε*-WO_3_ (Pc, #7)** | | | | | | | |
| --- | --- | --- | --- | --- | --- | --- | --- | --- | --- | --- | --- | --- | --- | --- | --- |
| **Sample** | ***d*_XRD_ / nm** | ***ε* / %** | ***a* / Å** | ***b* / Å** | ***c* / Å** | ***β* / °** | **R_Bragg_ / %** | ***d*_XRD_ / nm** | ***ε* / %** | ***a* / Å** | ***b* / Å** | ***c* / Å** | ***β* / °** | **R_Bragg_ / %** |  |
| ^a^ Pure WO_3_ (only *γ*) | 23.4 | – | 7.336 | 7.511 | 7.681 | 90.780 | 1.861 | – | – | – | – | – | – | – |  |
| ^b^ Pure WO_3_ (*γ*-rich, Figure S1) | 23.3 | – | 7.319 | 7.521 | 7.675 | 90.715 | 0.727 | 16.3 | – | 5.280 | 5.169 | 7.687 | 91.469 | 0.702 |  |
| Si-stabilized ε-WO_3_ | – | – | – | – | – | – | – | 19.4 | 0.253^c^ | 5.278 | 5.235 | 7.658 | 90.767 | 0.934 |  |

^a^ Diffractogram of pure WO_3_ analyzed with a *γ*-only contribution.
^b^ Diffractogram of pure WO_3_ analyzed with a mixture of *γ*- and *ε*-polymorphs, quantified at ~ 80 and 20 wt%, respectively.
^c^ Refinement of microstrain in the other cases yielded $\varepsilon(\%)\approx{10}^{-5}$, and was therefore not included in the model.

**Table S2:** Analysis of W L_3_-edge EXAFS. The (passive) amplitude reduction factor (*S*_0_^2^) is determined (from W foil) to be 0.868 ± 0.075. The *E*_0_ parameters are kept constant for the different scattering paths of each spectrum. The *α*- and *β*-sites refer to (multi-)scattering calculations performed considering as absorber distinct crystallographic sites of WO_3_ (ICSD #80056), i.e., with fractional coordinates (0.2513, 0.0277, 0.2865) and (0.2481, 0.0342, 0.7815), respectively.

| **W foil** *R*-factor: 0.021 *E*_0_ = 5.42 ± 0.91 eV |  |  |  |  |
| --- | --- | --- | --- | --- |
| Scattering path | $N / -$ | $R / Å$ | $\sigma^{2}/ Å^{2}$ |  |
| W – W(1) | 8^a^ | 2.736 ± 0.004 | 0.0028 ± 0.0004 | First shell |
| W – W(2) | 6^a^ | 3.154 ± 0.007 | 0.0034 ± 0.0006 | Second shell |
| W – W(1) – W(2) | 48^a^ | 4.313 ± 0.005 | 0.0045 ± 0.0005 | Multiple scattering |
| ***γ*-WO_3_** R-factor: 0.019 *E*_0_ = 12.59 ± 1.89 eV |  |  |  |  |
| (*α*) W – O(1) | 2.74 ± 0.25 | 1.788 ± 0.013 | 0.0015 ± 0.0002^b^ | Crystallographic site (*α*) |
| (*β*) W – O(1) | 1.37 ± 0.12 | 1.946 ± 0.013 | 0.0015 ± 0.0002^b^ | Crystallographic site (*β*) |
| (*β*) W – O(2) | 1.37 ± 0.12 | 2.438 ± 0.013 | 0.0015 ± 0.0002^b^ | Crystallographic site (*β*) |
| ***ε*-WO_3_** R-factor: 0.016 *E*_0_ = 12.40 ± 1.93 eV |  |  |  |  |
| (*α*) W – O(1) | 2.62 ± 0.25 | 1.787 ± 0.012 | 0.0010 ± 0.0004^c^ | Crystallographic site (*α*) |
| (*β*) W – O(1) | 1.31 ± 0.13 | 1.945 ± 0.012 | 0.0010 ± 0.0004^c^ | Crystallographic site (*β*) |
| (*β*) W – O(2) | 1.31 ± 0.13 | 2.437 ± 0.012 | 0.0010 ± 0.0004^c^ | Crystallographic site (*β*) |

^a^ Fixed parameters. ^b,c^ A single fitting parameter is used to describe the mean-square displacement (*σ*^2^) of W-O in the 1^st^ coordination shell.

**Table S3:** Comparison of band gaps of *γ*-WO_3_ calculated using DFT-1/2 with experimental values and DFT calculations using different functionals from previous reports.

| **Experiment**  **/ DFT** | **Code /**  **potential** | ***a* / Å** | ***b* / Å** | ***c* / Å** | ***β* / °** | ***E*_g_ / eV** | **Ref** |
| --- | --- | --- | --- | --- | --- | --- | --- |
| Experiment | ––– | 7.33 | 7.56 | 7.72 | 90.5 | 2.6 – 3.2 | ^49,69,72^ |
| Exp & DFT | CASTEP/  LDA | 7.24 | 7.46 | 7.61 | 90.8 | 0.87 | ^70^ |
| Exp & DFT | CASTEP/  PBE | 7.24 | 7.46 | 7.61 | 90.8 | 0.96 | ^70^ |
| DFT | PW91 | 7.56 | 7.80 | 7.84 | 90.1 | 1.36 | ^71^ |
| DFT | HSE06 | 7.39 | 7.64 | 7.75 | 90.3 | 2.80 | ^66^ |
| DFT | Siesta/  DFT-1/2 | 7.46 | 7.68 | 7.88 | 90.4 | 2.48 | ^26,30^ |
| **This work** | VASP/  DFT-1/2 | 7.45 | 7.62 | 7.78 | 90.5 | 2.99 |  |

**Table S4:** DFT-derived structures of *γ*-WO_3_ and *ε*-WO_3_.

|  | ***a* / Å** | ***b* / Å** | ***c* / Å** | ***β* / °** |
| --- | --- | --- | --- | --- |
| ***γ*-WO_3_** | 7.448 | 7.621 | 7.776 | 90.451 |
| ***ε*-WO_3_** | 5.345 | 5.261 | 7.758 | 91.248 |

**Table S5:** V_O_ formation energy for *γ*- and *ε*-polymorphs.

| System | V_O_ formation energy / eV |
| --- | --- |
| *γ*-WO_3-x_ (lattice V_O_) | 3.733 |
| *γ*-WO_3-x_ (surface V_O_) | 3.068 |
| *ε*-WO_3-x_ (lattice V_O_) | 3.655 |
| *ε*-WO_3-x_ (surface V_O_) | 3.048 |

**Table S6:** Local Bader charge and electron transfer values following acetone adsorption.

| ***γ*-WO_3-x_ + acetone** | | | ***ε*-WO_3-x_ + acetone** | | |
| --- | --- | --- | --- | --- | --- |
| Atom | Bader charge (*q*_i_) | $\Delta q_{i}$ / \|*e*\| | Atom | Bader charge (*q*_i_) | $\Delta q_{i}$ / \|*e*\| |
| O | 6.720 | 0.720 | O | 6.680 | 0.680 |
| C_1_ | 3.553 | – 0.447 | C_1_ | 3.499 | – 0.501 |
| C_2_ | 3.480 | – 0.520 | C_2_ | 3.490 | – 0.510 |
| C_3_ | 3.857 | – 0.143 | C_3_ | 3.436 | – 0.564 |
| H_1_ | 1.163 | 0.163 | H_1_ | 1.228 | 0.228 |
| H_2_ | 1.005 | 0.005 | H_2_ | 1.183 | 0.183 |
| H_3_ | 1.029 | 0.029 | H_3_ | 1.146 | 0.147 |
| H_4_ | 1.083 | 0.083 | H_4_ | 1.023 | 0.023 |
| H_5_ | 1.041 | 0.041 | H_5_ | 1.150 | 0.150 |
| H_6_ | 1.014 | 0.014 | H_6_ | 1.123 | 0.123 |
| **Δ*Q*** | | **– 0.055** | **Δ*Q*** | | **– 0.040** |

Negative values of *q*_i_ and/or Δ*Q* mean that the atom/molecule loses electrons.

Note: the numbering scheme of C and H atoms is as shown below, where the molecule is in the relaxed adsorption geometry from Figure 3a.


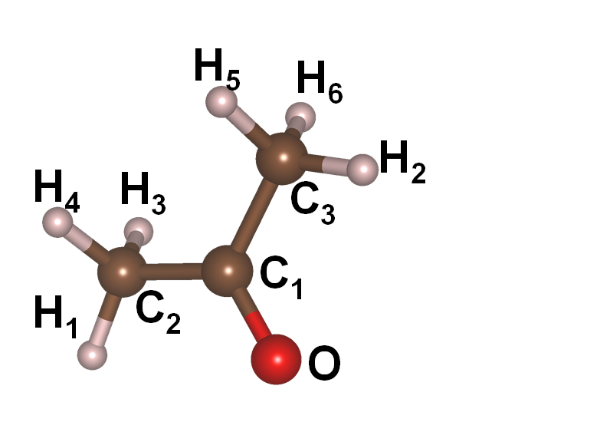


**Table S7:** Electrostatic and dipole moment (*μ*) calculations for *γ*- and *ε*-WO_3_ (001), *γ*- and *ε*-WO_3-x_ with bulk-only V_O_ (i.e., V_O_^bulk^) as well as both surface and bulk V_O_.

| **System** | **^a^** *E*_vacuum_ / eV | **^b^** *E*_Fermi_ / eV | **^c^** *ϕ* / eV | *μ* / Debye |
| --- | --- | --- | --- | --- |
| *ε*-WO_3_ (001) | 5.284 | – 2.212 | 7.496 | 0.007 |
| *ε*-WO_3-x_ (001) V_O_^bulk^ | 5.346 | – 1.098 | 6.444 | 0.218 |
| **^d^** *ε*-WO_3-x_ (001) top layer | 4.419 | – 1.473 | 5.892 | – 1.226 |
| **^e^** *ε*-WO_3-x_ (001) bottom layer | 6.228 |  | 7.701 |  |
| *γ*-WO_3_ (001) | 5.425 | – 2.043 | 7.468 | – 0.002 |
| *γ* -WO_3-x_ (001) V_O_^bulk^ | 5.495 | – 0.873 | 6.368 | 0.119 |
| *γ* -WO_3-x_ (001) | 5.526 | – 0.804 | 6.330 | – 0.993 |

^a^ *E*_vacuum_ is determined from the electrostatic potential profiles.
^b^ *E*_Fermi_ is directly determined from the static self consistency iteration.
^c^ *ϕ* = *E*_vacuum_ – *E*_Fermi_

^d,e^ There is asymmetry between top and bottom facets (see Figure S23).
